# Transmission of Radio‐Frequency Waves and Nuclear Magnetic Resonance in Lanthanum Superhydrides

**DOI:** 10.1002/advs.202520701

**Published:** 2026-02-08

**Authors:** Dmitrii V. Semenok, Florian Bärtl, Di Zhou, Toni Helm, Sven Luther, Joachim Wosnitza, Ivan A. Troyan, Viktor V. Struzhkin, Hannes Kühne

**Affiliations:** ^1^ Center For High Pressure Science & Technology Advanced Research Beijing China; ^2^ Hochfeld‐Magnetlabor Dresden (HLD‐EMFL) and Würzburg‐Dresden Cluster of Excellence ctd.qmat Helmholtz‐Zentrum Dresden‐Rossendorf Dresden Germany; ^3^ Institut für Festkörper‐ und Materialphysik Technische Universität Dresden Dresden Germany; ^4^ Max Planck Institute for Chemical Physics of Solids Dresden Germany; ^5^ A.V. Shubnikov Institute of Crystallography of the Kurchatov Complex of Crystallography and Photonics Moscow Russia; ^6^ Shanghai Key Laboratory of Material Frontiers Research in Extreme Environments (MFree) Shanghai Advanced Research in Physical Sciences (SHARPS) Shanghai China; ^7^ Center For High Pressure Science & Technology Advanced Research Shanghai China

**Keywords:** high pressure, NMR, radio‐frequency methods, superconductivity, superhydrides

## Abstract

The discovery of near‐room‐temperature superconductivity in the lanthanum hydride LaH_10_ has revolutionized this research field. However, the need to use diamond anvils for the synthesis of hydride superconductors severely limits the experimental techniques to study these materials. Nuclear magnetic resonance (NMR) is one of the key methods for probing spin systems of superconductors. Here we show how ^1^H NMR can be realized in diamond anvil cells to study lanthanum polyhydrides at pressures up to 165 GPa. In the newly discovered superhydride LaH_12_, we observed a pronounced suppression of the ^1^H NMR signal intensity below *T*_c_^onset^ = 260 K in a magnetic field of 7 T, corresponding to the screening of the radio‐frequency pulses. Below the critical temperature, all ^1^H NMR characteristics, including the nuclear spin‐lattice relaxation rate 1/*T_1_T*, exhibit pronounced features that may be associated with superconductivity. In zero field, the radio‐frequency signal transmission shows a pronounced drop below *T*_c_^onset^ ≈ 267‒279 K, indicating the very beginning of the transition in the most ideal microcrystals. A description of the 1/*T_1_T* data with an exponential form allows the estimation of the superconducting gap Δ(0) = 427‒671 K (corresponding to 36.8‒57.8 meV), and the ratio *R*_Δ_ = 2Δ*(0)*/*k*_B_ *T*_c_ between 3.76 and 5.16 in the synthesized polyhydride.

## Graphical abstract

^1^H NMR and radio‐frequency transmission measurements of lanthanum superhydrides at 165 GPa reveals a transition at 260–280 K by a strong suppression of the signal intensity, significant changes in NMR spectra, including a dramatic decrease of the relaxation rate 1/*T_1_T*, and pronounced shielding effect, which may have a superconducting nature.

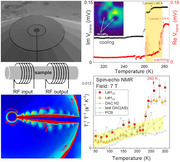

## 1 Introduction

**FIGURE 1:**
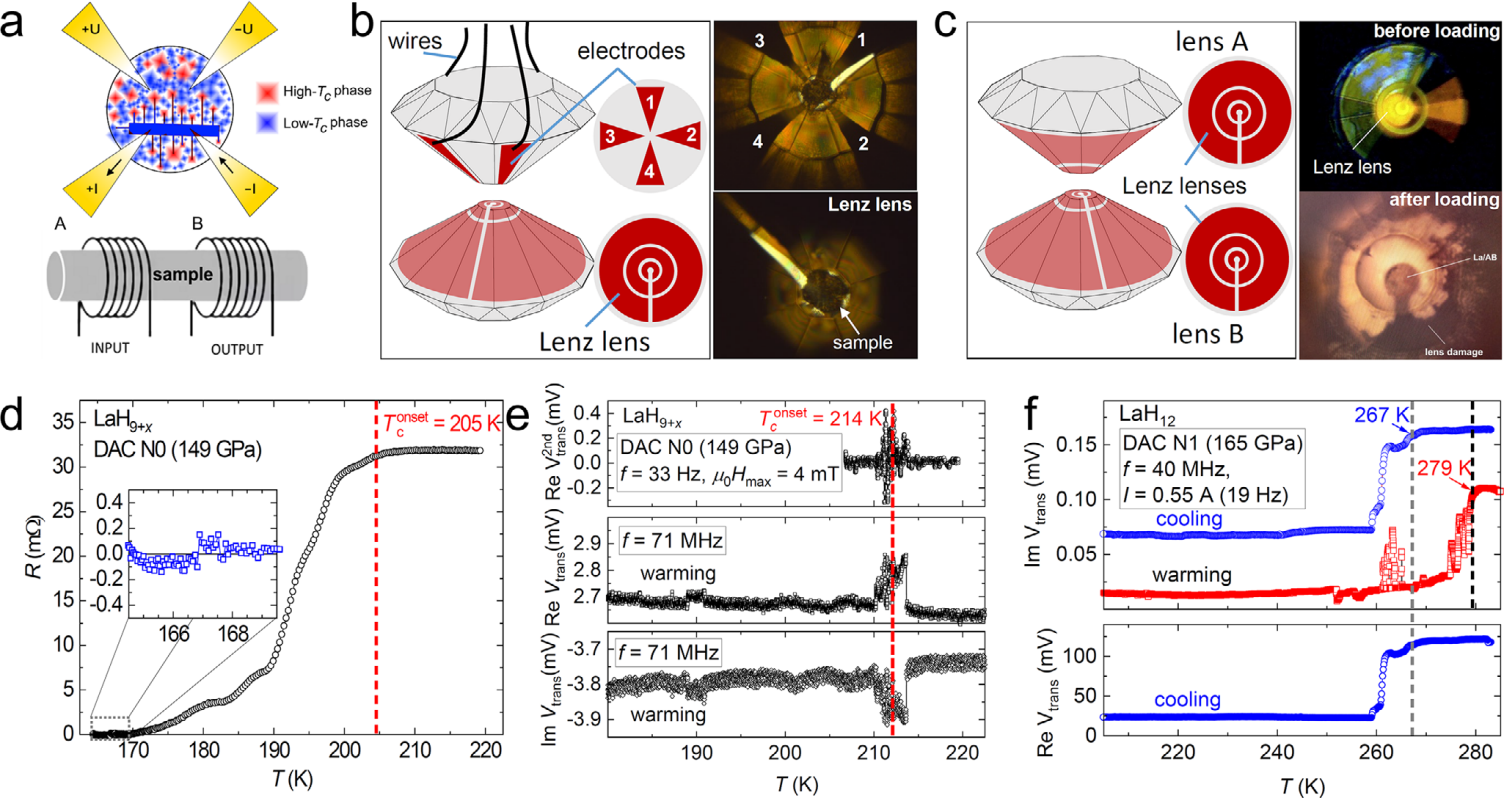
Electrical resistance and RF transmission of lanthanum superhydrides, synthesized from La and NH_3_BH_3_ in the DACs N0 and N1. (a) Sketch of a possible distribution of high‐*T*_c_ and low‐*T*_c_ superconducting phases in a hydride sample, which complicates the detection of high‐*T*_c_ SC using a four‐electrode van der Pauw circuit. At the same time, only the low‐*T_c_* transition may be detected due to the presence of a homogeneous segment of the corresponding phase (marked in blue) between the electrodes. Also shown is a scheme of RF diagnostics, employing a transformer circuit (coil A is input, coil B is output). (b) Schematic diagram of the DAC N0, containing a four‐electrode circuit for transport measurements and a Lenz lens for RF transmission measurements. Right: Images of the DAC culet, loaded sample, electrode system and Lenz lens. (c) Schematic diagram of the DAC N1, containing two Lenz lenses for RF transmission measurements and NMR studies. Right: Images of the anvil culet with sputtered Lenz lenses before and after loading and compression to 165 GPa. (d) Electrical resistance (current is 1 mA, DC) as a function of temperature for a sample of lanthanum hydride in DAC N0 at 120 GPa. Inset: residual resistance of the sample below 170 K. (e) Real (Re) and imaginary (Im) components of the RF signal passing through the DAC N0 sample in transformer mode. The carrier frequency is 71 MHz. Top panel: Directly demodulated RF signal of the second harmonic of the low‐frequency modulating field (33 Hz, *B*_max_ ≈ 4 mT). (f) High‐frequency (40 MHz) study of the DAC N1 at 165 GPa. Real and imaginary components of the receiving coil signal in the cooling cycle. The superconducting transition of the sample in DAC N1 starts around 267 K. A thermal hysteresis (about 10 to 12 K) in the system as exemplified by the imaginary (Im) components of the receiving coil signal. The thermometer was glued to the outside of DAC N1.

Superhydrides are a rapidly developing class of hydrogen‐rich compounds that have attracted considerable attention in the field of condensed‐matter physics due to their remarkable superconducting properties. The experimental discovery of high‐temperature superconductivity (SC) in H_3_S ($T_{c}^{\max}\;$= 203 K [1]), LaH_10_ ($T_{c}^{\max}$ = 250 K [2, 3]), ThH_10_ ($T_{c}^{\max}\;$= 161 K [4]), YH_6_ ($T_{c}^{\max}$ = 224‐226 K [5, 6]), CeH_10_ ($T_{c}^{\max}$ = 115 K [7]), and many other polyhydrides [8, 9], was preceded by numerous theoretical predictions [10, 11, 12, 13], intensive development of computer programs (for example, Quantum Espresso [14, 15], VASP [16], USPEX [17, 18], CALYPSO [19], AIRSS [20]), and ab initio methods for calculating superconducting properties (for instance, SCDFT [21, 22] and SSCHA [23]). Eventually, it has opened new avenues for fundamental and applied studies. Particularly, the search for room‐temperature superconductors and the development of magnetic field sensors, diodes, and memory elements based on superhydrides gave a new impetus to the research efforts [24, 25, 26]. Unfortunately, polyhydride samples are often inhomogeneous [27, 28]. A comprehensive study of SC in such inhomogeneous samples is problematic due to the presence of several crystallographic phases, including metastable ones [29], with very different critical temperatures (Figure 1a). Indeed, impurity phases or grains, separated from the probing current trajectories by non‐superconducting lower hydrides or insulating shells of higher polyhydrides, cannot be identified by standard four‐probe transport measurements (Figure 1a). Such grains with varying superconducting properties may be responsible for broadened or steplike transitions in the temperature dependence of the resistance, hardly distinguishable inhomogeneous sample quality, or stress distributions. Detection methods based on volume‐penetrating electromagnetic fields can be suitable probing techniques in this situation. Radio‐frequency (RF) contactless measurements [30, 31] and nuclear magnetic resonance (NMR) spectroscopy [32] allow to overcome this problem and detect SC in inhomogeneous samples under pressure.

NMR spectroscopy is based on the coherent manipulation of the nuclear spin system in a material [33, 34]. It can be employed to probe the magnetic hyperfine fields, originating from the electronic subsystem. Thus, it is very sensitive to the static electronic spin susceptibility and low‐energy excitations of the electronic system. The shape of an NMR spectrum, its broadening and frequency shift depend on the magnetic interactions of the nuclear moments with their environment [35]. ^27^Al‐NMR spectroscopy played a major role in confirming the Bardeen–Cooper–Schrieffer (BCS) theory of SC [36]. Moreover, Redfield, Anderson, Hebel, and Slichter discovered that at temperatures below the superconducting critical temperature (*T*_c_), there is a maximum of the ^27^Al spin‐lattice relaxation rate 1/*T*_1_ (the Hebel‐Slichter peak) in aluminum [37, 38, 39]. Measurements of 1/*T*_1_ at temperatures well below *T*_c_ yield an exponential decrease of 1/*T*_1_ with temperature in the case of a full superconducting gap. In metals, the NMR frequency shift (also called Knight shift [40]) and the spin‐spin relaxation rate 1/*T*_2_ are independent of temperature [41], whereas 1/*T*_1_ is proportional to temperature (known as Korringa relation [42]). A distinctive feature of a superconducting transition is a sharp decrease of the relaxation rate 1/*T_1_T* due to the opening of the superconducting gap.

Since the beginning of research on superhydrides, it was suggested that they were the most prominent representatives of BCS superconductors [1]. NMR spectroscopy is a powerful tool for studying the nature of the normal and superconducting ground states. Therefore, it can help to obtain information on the superconducting gap in the polyhydrides. However, NMR studies on these materials face a major experimental challenge—the tiny sample space of a diamond anvil cell (DAC), which is required to create the necessary pressures of over 1 million atmospheres (100 GPa). Here, the comparably high sensitivity of NMR spectroscopy to the ^1^H isotope, the sublattice of which plays a major role in the SC of hydrides [8], is very beneficial. Fortunately, an approach has recently been developed that allows the detection of ultra‐small samples via conventional solid‐state NMR techniques, using focusing Lenz lenses deposited on diamond anvils [32, 43, 44]. Lower hydrides of FeH [45, 46], Cu_2_H [47], YH_3_ [32], LaH_3_ [44], and molecular hydrogen [48, 49], have previously been studied at pressures up to 220 GPa. Below, we will demonstrate that the same Lenz lenses also prove to be a very efficient tool for studying the surface impedance of superhydride samples. Unlike NMR, within this RF transmission method, two Lenz lenses are used separately as excitation (A) and detection (B) microcoils of an RF transformer (Figure 1a).

In this work, we applied RF transmission and ^1^H NMR techniques to study lanthanum superhydrides, synthesized from La or LaH_∼3_ and ammonia borane (NH_3_BH_3_) in five DACs (labeled as “N0‐N4”) at different pressures up to 165 GPa. We were able to study the ^1^H NMR signal of various lanthanum polyhydrides and observed a transition that was probably of a superconducting nature, stemming from the newly discovered LaH_12_, yielding a *T*_c_^onset^ above 267 K. This is confirmed by an abrupt change in the RF surface impedance and a sharp drop of the ^1^H NMR spin‐lattice relaxation rate. In our interpretation, superconductivity is the primary hypothesis explaining the phenomena observed in the NMR and RF experiments. The high density of high‐*T_c_* superconducting hydrides in the La‐H system under pressure, as well as previous transport [3, 50] and magnetic measurements [51], make this hypothesis highly plausible.

## 2 Results and Discussion

### 2.1 Combined DAC with Electrodes and Lenz Lens

Although the RF method of measuring the surface impedance has been known since 1989 [30], and has been widely used to probe superconducting transitions [31, 52], it has not been applied in combination with Lenz lenses [53] for high‐pressure studies. To bridge this gap, in the first (calibration) experiment utilizing the DAC N0 (Figure 1a–e), we used two detection systems simultaneously: a classical four‐probe van der Pauw resistance measurement circuit was deposited on one diamond anvil, while a simple single‐stage Ta/Au Lenz lens (Figure 1b) was sputtered onto the opposite anvil. After laser heating of La microparticles loaded with ammonia borane (NH_3_BH_3_) at 149 GPa, the pressure in the DAC dropped to 120 GPa, and the four‐contact measurements showed a pronounced resistance drop starting below *T*_c_^onset^ = 205 K (Figure 1d). This *T*_c_ is in good agreement with previous studies of the La superhydrides [54, 55]. The result of our synthesis may correspond to the hexagonal polyhydride *P*6_3_/*mmc*‐LaH_9‐10_ [56, 57] or low‐symmetry (e.g., *C*2/*m* [54, 58]) LaH_9+x_ (x = ± 1) with a partially molecular hydrogen sublattice [59]. A detailed description of the preparation and characteristics of the sample in DAC N0 is given in the Table S1.

Using any electrode pair of the DAC N0 as an RF antenna and the Lenz lens with a surrounding, macroscopic single‐turn coil as a receiver (Figures S1 and S2) allowed us to detect a sharp change in the surface impedance of the sample in the real and imaginary components of the RF signal. This corresponds to a SC transition with an onset at 212 to 214 K (Figure 1e). We detected such steps or jumps of the transmitted RF signal using various frequencies between 0.2 and 71 MHz (Figure 1e; Figure S3), confirming the reproducibility. For additional verification, we used the double‐modulation technique, similar to that proposed by Timofeev in 2002 [31]. As shown in the top panel of Figure 1e, placing the sample in a weak (*B*_max_ ≈ 4 mT) oscillating magnetic field with a frequency of 33 Hz leads to a pronounced feature in the second harmonic (66 Hz) of the directly demodulated RF signal. This feature emerges simultaneously with the jump in the transmitted RF signal, because the magnetic field suppresses SC, regardless of the sign of the magnetic induction *B_z_*.

The *T*_c_ observed by the RF method is somewhat higher than *T*_c_^onset^, detected my means of the resistance measurements. This is because the high‐frequency field penetrates the entire volume occupied by the LaH_9+x_ sample, and not only the space between electrodes, as in the case of the four‐electrode circuit. That is why it is possible to detect superconducting phases with the highest *T_c_* regardless of how far they are from the electrodes. Note that reducing the carrier frequency to 0.2–1 MHz also allows for reliable detection of the signal of only the “main” superconducting phases, located near the electrodes, with *T*_c_ at about 180 to 190 K (Figure S3). Further radio‐frequency transmission tests using MgB_2_, NbTi, Bi2212, YBCO, REBCO, La_3‐x_Nd_x_Ni_2_O_7_ (La,Ce)H_10‐12_, and (La,Sc)H_x_ can be found in the Refs. [60, 61, 62, 63].

### 2.2 RF Detection of Superconductivity with Two Lenz Lenses

After successful testing of the contactless RF detection method using Lenz lenses (DAC N0), we prepared DACs containing two Lenz lenses as indicated in Figure 1c (see Supporting Information) for combined RF transmission and ^1^H NMR studies. We performed experiments with four high‐pressure DACs (pressure is given in brackets): DAC N1 (165 GPa), N2 (147 GPa), N3 (87 GPa), and N4 (19 GPa). The samples in the DACs N1 and N2 were obtained by infrared laser heating of metallic La in an NH_3_BH_3_ environment, while the samples N3 and N4 were obtained by heating of LaH_3‐_ *_x_* with NH_3_BH_3_ under pressure. We performed the laser heating at the ID27 beamline of the European Synchrotron Radiation Facility (ESRF). The most interesting results were obtained for the samples N1 and N3, synthesized in symmetric BX‐90 mini cells [64], equipped with diamond anvils with a culet diameter of 75 µm. One to two micrometers thick copper or silver films, sputtered onto the diamond anvils, were used for the Lenz lenses [53] (Figure 1c; Figure S1). As we will demonstrate in the next paragraph, the sample in DAC N1 is best described by the formula LaH_12_. Detailed information on the DAC designs and preparation is given in the Table S1.

First, we performed RF transmission measurements of the sample in DAC N1 at 165 GPa, employing a high‐frequency transformer formed by the two Lenz lenses. As can be seen in Figure 1f (as well as in Figure S4), the RF signal transmission exhibits main features at 267 to 279 K, which appear in both the amplitude and phase of the output signal of the RF transformer for various frequencies (40, 150, and 200 MHz). This behavior is accompanied by the appearance of a second harmonic at the frequency of the external modulation field (Figure S4). Another small feature in the RF transmission occurs as well at 248 to 252 K, which probably corresponds to a LaH_10_ impurity (Figure S5). Previous works on lanthanum [3, 50, 51, 65] and lanthanum‐scandium [26] hydrides indeed evidenced an additional step‐like drop in the electrical resistance, setting in at temperatures between 265 and 279 K or even above. However, such a high *T*_c_ could not be attributed to any known La hydride until now. In the present work, we demonstrate that this high‐*T*_c_ hydride phase can be detected by contactless methods, in particular by changes in the surface RF impedance. The RF method reported here, indicates that SC in the La‐H system under pressure may occur already at temperatures as high as 267 to 279 K [26, 51].

### 2.3 X‐Ray Diffraction of the Synthesized Lanthanum Superhydrides

**FIGURE 2:**
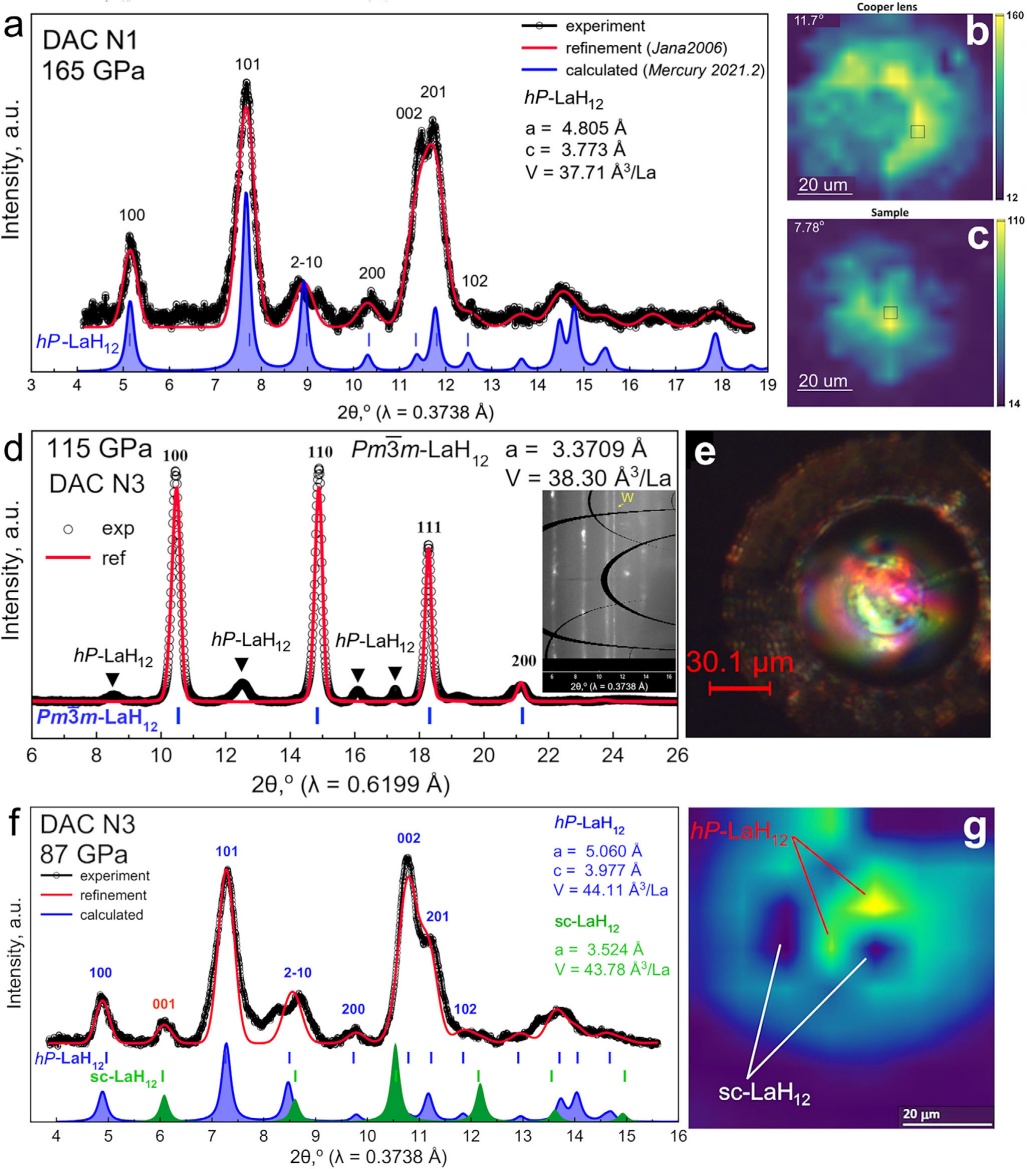
X‐ray diffraction studies of the samples in DAC N1 at 165 GPa and in DAC N3 at 87‐115 GPa. (a) X‐ray diffraction pattern of the sample in DAC N1 and Le Bail refinement of the *hP*‐LaH_12_ unit‐cell parameters. Black circles are the experimental data, the red line is the Le Bail refinement, and the blue line is the XRD pattern calculated using the Mercury 2021.2 software [67]. For the refinement, we used the *P*6_3_/*mmc*‐LaH_9‐10_ structure [56, 57], which reproduces almost all diffraction peaks, but has an unusually small *c/a* ratio of 0.78. The unit‐cell volume corresponds to a hydrogen content of about 12 H atoms per La atom. (b) X‐ray diffraction mapping of a copper Lenz lens, and (c) the *hP*‐LaH_12_ distribution, obtained using the XDI software [68]. The color scale corresponds to the intensity of the strongest XRD reflection for each compound. (d) X‐ray diffraction pattern of the sample in DAC N3 and Le Bail refinement of the *Pm* $\bar{3}$ *m*‐LaH_12_ unit‐cell parameters. The sample also contains an impurity fraction of hexagonal *hP*‐LaH_12_. Black circles are the experimental data, the red line is the Le Bail refinement. (e) Picture of the DAC N3 culet with the sample. (f) XRD pattern of the sample in DAC N3 after decompression to 87 GPa (see also Figure S17). Black circles are the experimental data, the red line is the Le Bail refinement, and the blue and green filled curves are the XRD patterns of both LaH_12_ modifications, calculated using the Mercury 2021.2 software [67]. (g) X‐ray diffraction map of the *hP* (yellow‐green) and *Pm* $\bar{3}$ *m*‐LaH_12_ (dark blue) spatial distributions (XDI software [68]).

Diffraction of synchrotron radiation is an effective technique for elucidating the structure of polyhydrides under high pressure. The structures of the lanthanum superhydrides in the DACs N1, N2, and N3 were studied using powder X‐ray diffraction (XRD), presented in Figure 2). In the case of DAC N1, our XRD analysis shows the formation of a new crystal modification of LaH_12_ at 165 GPa, most likely with a hexagonal symmetry (e.g., *P*6_3_/*mmc*, Figure 2a). In general, an interpretation of the XRD data of the sample in DAC N1 at 165 GPa is challenging. The resulting pattern differs from all the previously experimentally studied ones for the La‐H system, but has many similarities with the recently discovered (La,Ce)H_12_ [24]. In terms of volume, the obtained compound approaches the one of LaH_12_ [2]. Although this powder pattern also allows for rhombohedral solutions (e.g., *R* $\bar{3}$ *m*), we will use the *hP* notation due to the analogy with the theoretically proposed *P*6_4_‐ScH_12_ [66].

Although the *P*6_3_/*mmc* structure, with the lattice parameters *a* = 4.805 Å and *c* = 3.773 Å, and a unit‐cell volume of *V* = 37.71 Å^3^/La, describes the observed diffraction pattern quite well (Figure 2a–c), it should be noted that a *c/a* ratio of 0.78 is unusually small for the *P*6_3_/*mmc* space group. Moreover, the reflections 002 and 201 have unexpectedly high intensities, and the 2‐10 reflection is actually a split peak due to superstructural features of LaH_12_ or lattice distortion. Hence, another (lower) space group may also describe the structure of the obtained compound. We set the amount of hydrogen in *hP*‐LaH_12_ by analogy with the previously reported simple cubic (sc) *Pm* $\bar{3}$ *m*‐LaH_12_ [2, 69, 70, 71] with an accuracy of ± 1 H atom. As observed for many known polyhydrides ((La,Y)H_10_ [72, 73], CeH_9‐10_ [7], ThH_9‐10_ [4], PrH_9_ [74], etc.), the hexagonal and cubic crystalline modifications form simultaneously, accompany each other, and in many situations cannot be separated or obtained selectively. The same is observed in the case of LaH_12_. Taking into account subsequent radio‐frequency and NMR measurements, this means that we cannot unambiguously attribute the observed transitions to either the hexagonal or cubic phase of LaH_12_.

The sample in DAC N3, consisting of two phases, was initially synthesized at a pressure of about 115 GPa from LaH_3‐_ *_x_* and NH_3_BH_3_. The first phase detected after the pressure synthesis (115 GPa) was simple cubic (*Pm* $\bar{3}$ *m*) sc‐LaH_12_ (Figure 2d,e), as reported previously [2, 69, 70, 71]. Structural models for LaH_12_ were proposed by theoretical groups of Defang Duan [66] as *Pm* $\bar{3}$‐ScH_12_, *Pm* $\bar{3}$‐MgH_12_, and rare hexagonal *P*6_4_‐ScH_12_, and Feng Peng as *Pm* $\bar{3}$ *m*‐LuH_12_ [75]. According to their DFT calculations, which included anharmonic effects, these compounds are high‐temperature and even room‐temperature superconductors (*T_c_* > 300 K). In these structures, hydrogen exists in an intermediate state (d_HH_ = 1.1–1.2 Å) between molecular (d_HH_ = 0.7–0.8 Å) and atomic hydrogen (d_HH_ = 1.4–1.6 Å). This intermediate state may have a shallow energy minimum. However, in the harmonic approximation *Pm* $\bar{3}$ *m*‐LaH_12_ is dynamically unstable [66]. The anharmonic case will be the subject of future works.

The second phase, distributed in close proximity to LaH_12_, is the hexagonal modification *hP*‐LaH_12_, which is the main product of the synthesis in DAC N1. This *hP* phase has almost the same volume as the cubic one (Figure 2f,g). Given that both modifications of LaH_12_ have been virtually unknown previously, we decided to focus on their equation of state and stability at low pressures. Successfully, in both DACs N1 and N3, we were able to reduce the pressure to 20‐40 GPa without cracking the diamond anvils, and determine the volume and unit cell parameters (Figure 4c,d). Both synthesized modifications of LaH_12_ generally retain the structure of their La sublattices upon decompression to 40 GPa and below (Figures 2f and 4c,d; Figure S17). We expect that as the pressure decreases, the hydrogen sublattice of these compounds changes significantly and becomes more molecular, which is also reflected in the width of the XRD peaks, especially below 82 GPa (see Figure 4c; Figure S17).

DAC N2, made from BeCu alloy, was loaded with metallic La and NH_3_BH_3_, and heated via a series of IR laser pulses at 147 GPa at the ID27 station of the ESRF. After the laser heating, the pressure increased to 150 GPa. For this sample, we used diamond anvils with a culet diameter of 100 µm. As will be discussed below, the ^1^H NMR signal of the sample in DAC N2 is several times smaller compared to the spectral intensity observed for the samples in DACs N1 and N3. This likely is caused by a partial destruction of one of the Lenz lenses, as well as a smaller amount of synthesized superhydrides.

According to the XRD analysis (Figure S13), there are two small regions of ∼10 µm inside of DAC N2, containing mainly *I*4/*mmm*‐LaH_4_ and *Cmcm*‐LaH_3_. The first of these compounds is a superconductor with a *T*_c_ of about 80 to 90 K [76, 77], while the second one is an analogue of the previously found *Cmcm*‐LaH_∼4_ [28] with a 2 Å^3^/La smaller unit‐cell volume. This corresponds to a LaH_3_ composition. The *T*_c_ of tetragonal LaH_4_ was reported to decrease rapidly in an applied magnetic field (*dT*_c_/*dB* ≈ – 2 K/T [76]). Therefore, *T_c_* should be 66 to 76 K at 7 T. Due to the low content of the superconducting phase and the large spurious background signal with features below 100 K, we cannot confidently state that we have detected the anticipated superconducting transition in LaH_4_. However, the disappearance of a series of ^1^H NMR signals, and a step in the 1/*T_1_* vs *T* dependence around 60 to 70 K may indicate the emergence of SC in LaH_4_ (Figure S22).

DAC N4 was loaded with LaH_3‐_ *_x_* and NH_3_BH_3_. The material was heated by an IR laser at around 150 GPa, but it broke down in the following days with a pressure drop to below 50 GPa. Due to this, DAC N4 was used only for a narrow set of experiments (Figure S23).

### 2.4 Preliminary NMR Experiments

^1^H NMR experiments on micron‐sized samples compressed between diamond anvils are inevitably associated with parasitic hydrogen signals from different parts of the high‐pressure DACs, such as the organic adhesive used to glue the diamond anvils to the seats, wire insulation, capacitors, soldering paste, and the printed circuit board (PCB) supporting the excitation coils. Since the laser heating of the starting material in the DACs never leads to a complete decomposition of NH_3_BH_3_, the study of the residual signal of this compound is important as well. Therefore, before starting the experiments with the polyhydrides in the DACs N1‐N4, we investigated several calibration samples at temperatures from 10 to 300 K. We studied the following test samples:

- An empty DAC with a Lenz‐lens system (Figure S8);
- The same DAC, loaded with a piece of ^27^Al foil and NH_3_BH_3_ (Figures S6 and S7);
- A bulk sample of NH_3_BH_3_ in a conventional NMR resonator (Figure S9);
- A piece of PCB, used as support for the Lenz‐lens excitation coils (Figure S10).

The examination of the empty DAC with Lenz lenses (test “A”) indicates the presence of some volatile H‐containing impurities in the DAC: The NMR signal intensity significantly decreases with time. The low intensity of the ^1^H NMR signal of the empty DAC prevents a *T*_1_ measurement (Figure S8).

As a result of the preparatory experiment “B”, we found that the Lenz‐lens system does indeed allow a reliable detection of the ^27^Al NMR signal from a small piece of Al foil from the DAC sample volume. The spin‐lattice relaxation curve *I(τ)* and the stretching exponent indicate a non‐uniform distribution of the RF field in the sample (Figure S6), which in this case is due to a non‐ideal geometry of the Lenz lenses in the non‐pressurized DAC.

The ^1^H signal intensity was found to sufficiently strong when we placed a micron‐sized sample of NH_3_BH_3_ together with the ^27^Al foil in the chamber of the test DAC “B” (Figures S6 and S7). Besides the presence of a kink in the temperature dependence of the integral intensity *I*(*T*) around 250 K, the nuclear spin‐lattice relaxation rate 1/*T*_1_ *T* yields no particular features between 50 and 300 K, which is important to note for the subsequent experiments on the lanthanum hydrides.

The ^1^H NMR experiments on a bulk sample of NH_3_BH_3_ (test “C”) yield a rather complex, two‐component *T*_1_ relaxation. For instance, 1/*T*_1_ *T* shows a rise below 210 K, with a maximum at 100 to 150 K (Figure S9). This is possibly related to low‐temperature phase transitions in NH_3_BH_3_, as reported previously (see also Ref [78]).

Finally, the teflon PCB (test “D”) gives an insignificant ^1^H NMR signal without pronounced features above 170 K (Figure S10). Thus, by performing these preliminary experiments, we have verified that the ^1^H NMR signal discussed below indeed dominantly stems from the samples of compressed lanthanum polyhydrides.

### 2.5 ^1^H NMR of Lanthanum Superhydrides at Pressures up to 165 GPa

**FIGURE 3:**
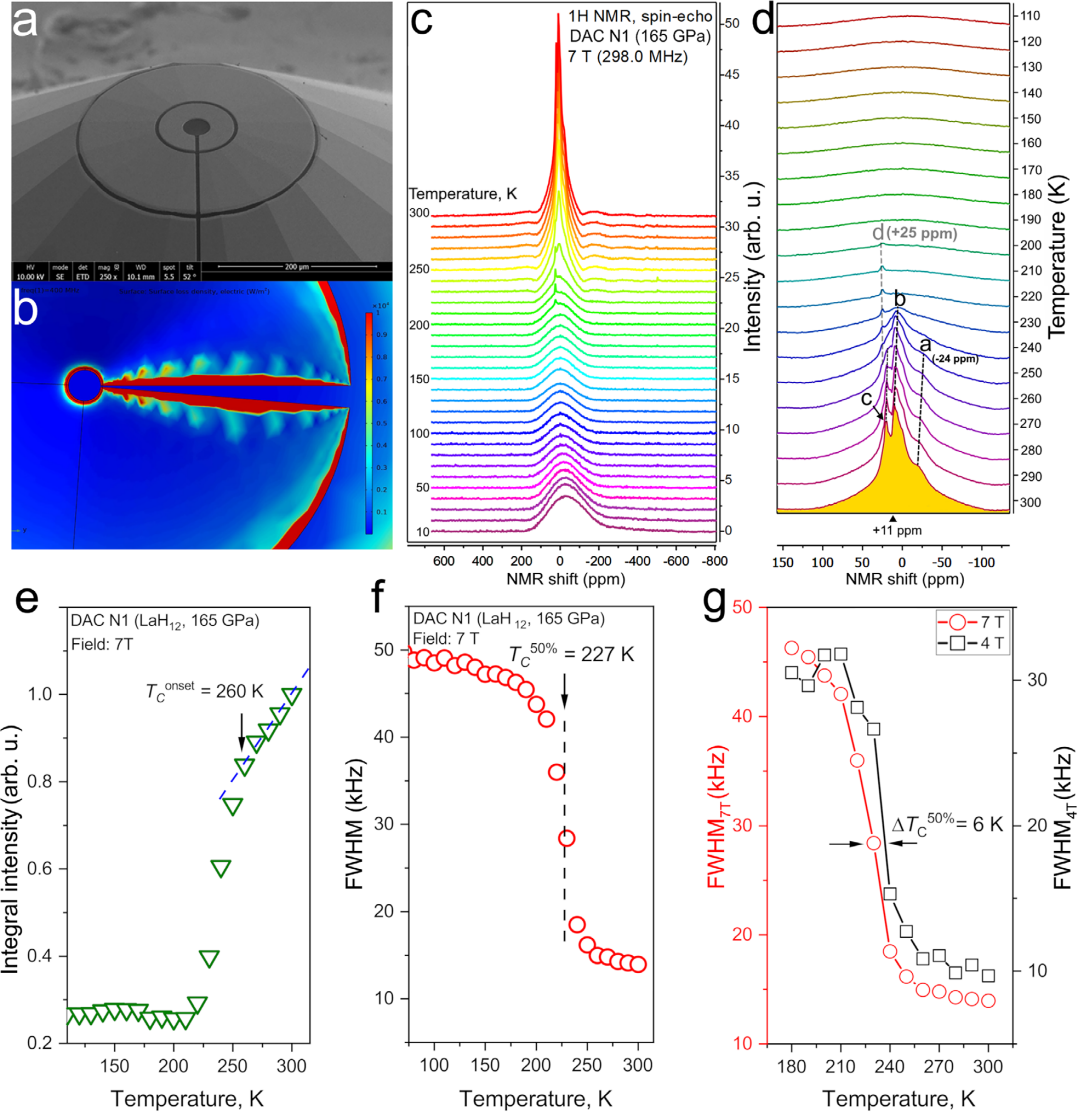
^1^H Nuclear magnetic resonance experiments using DAC N1. (a) Scanning electron microscopy of the copper Lenz lens of DAC N1, prepared by using a focused Ga‐ion beam. (b) Modeling of the distribution of surface current density losses (in W/m^2^) in the Lenz lens at 400 MHz (using COMSOL Multiphysics [79]). (c) Stack of ^1^H NMR spectra, recorded during cooling from 300 to 10 K in steps of 10 K. (d) ^1^H NMR spectra in the temperature regime of the superconducting transition. The structure of the signal above 240 K is composed of at least four different peaks (“a–d”). (e) Temperature dependence of the ^1^H NMR signal area (“intensity”) between 115 and 300 K. *T*_c_^onset^ = 260 K denotes the onset of the pronounced intensity decrease. (f) Temperature dependence of the ^1^H NMR linewidth (FWHM = full width at half maximum) between 75 and 300 K, yielding *T*_с_(50 %) = (227 ± 5) K. (g) Temperature dependence of FWHM for applied magnetic fields of 4 and 7 T. At 4 T, *T*_c_ is approximately 6 K higher.

We used a system of Lenz lenses (Figure 3a,b), deposited on diamond anvils, to perform NMR measurements of micron‐sized samples under pressure. Our ^1^H NMR study, using DAC N1 in a magnetic field of 7 T (ν_0_ = 298 MHz), employing a conventional spin‐echo pulse sequence, was carried out during cooling between 300 and 10 K (Figure 3c). At high temperatures, the spectrum can be described as a composition of at least four peaks (“a–d”, Figure 3d), three of which (“a–c”) disappear below 230 K and yield a negative frequency shift with decreasing temperature (*dν/dT* > 0). These peaks are attributed to different lanthanum superhydrides that form at 165 GPa, when the La/ NH_3_BH_3_ mixture is heated [2, 28, 59]. As the XRD analysis demonstrated (Figure 2), the main phase synthesized in DAC N1 is hexagonal *hP*‐LaH_12_. The very narrow peak “d” disappears below 200 K with *dν/dT* < 0.

Between 300 K and *T*_c_^onset^ = 260 K, we find a linear decrease of the integral intensity of the ^1^H NMR spectrum, as well as a pronounced slope change at *T*_c_^onset^ (Figure 3e). Below *T*_c_^onset^, our data show a strong suppression of the NMR intensity, which saturates at 211 K. Between *T*_c_^onset^ and 220 K, we observe pronounced changes in all spectral characteristics, i.e., the frequency shift, spectral width, and area (“intensity”) of the ^1^H NMR spectrum (Figure 3d–f). The residual, broad low‐temperature ^1^H NMR spectrum stems from the background of non‐superconducting hydrogen‐containing compounds, residual NH_3_BH_3_, products of its thermal decomposition, and lower La hydrides (Figure 3c,d). We note that, although these background signals may partially contribute to the change in spectral characteristics in the superconducting transition region, this does not affect the qualitative behavior in the detection of the superconducting transition by means of NMR. At 165 GPa and 7 T, the observed *T*_c_^50%^ is (227 ± 5) K (Figure 3f). This temperature corresponds to the SC transition in the best‐known lanthanum superhydrides in a field of 7 T, determined via electrical‐transport measurements at the middle of the resistance drop (*R*_50%_ criterion, see Ref [2].). At 4 T, the superconducting transition of the lanthanum superhydride in DAC N1 is shifted higher in temperature, to *T*_c_ *^50%^* = (235 ± 5) K (Figure 3g). The offset *T*_c_ also increases from 211 K (7 T) to 220 K (4 T), see Figure S19.

**FIGURE 4:**
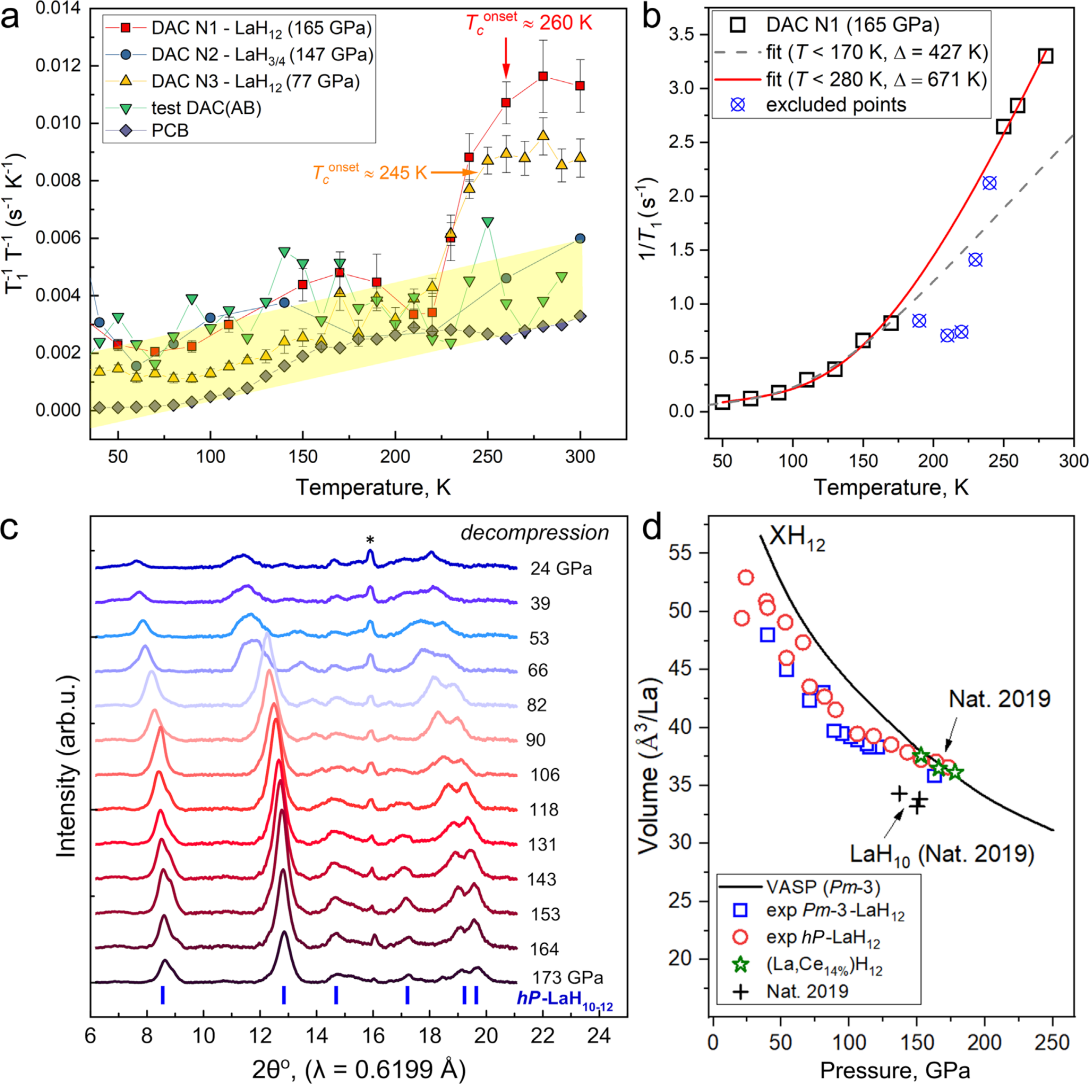
^1^H nuclear spin‐lattice relaxation rate (*T_1_T*)^−1^ and the equation of state of LaH_12_. (a) Temperature dependence of the relaxation rate 1/*T_1_T*. There is a pronounced drop in the relaxation rate of the samples in DACs N1 and N3 below 260 and 245 K, respectively. This is associated with the presence of the LaH_12_ phase, which is absent in the other DACs and test samples. (b) Fits using 1/*T*_1_ = *A*e^−Δ/^ *^T^* + *CT* (here we set *k*_B_ = 1) to describe the temperature‐dependent 1/*T*_1_ of the sample in DAC N1 at 165 GPa and 7 T. Here, *A* and *C* are fit parameters, as well as the superconducting gap Δ = Δ(0). We performed fits for temperatures below 170 and 280 K, indicated by a gray dashed line and a red solid line, respectively. (c) Stack of integrated XRD patterns obtained during decompression of the DAC N1 with hexagonal LaH_12_ from 173 GPa to 24 GPa at room temperature. (d) Equation of state *V(P)* of cubic (*Pm* $\bar{3}$ *m*) and hexagonal (*hP*) LaH_12_ compared with the theoretical calculations (black line) and data from Ref. [2] (“Nat. 2019”). [Correction added on 12 February 2026, after first online publication: Figure 4d is updated.]

To confirm the onset of SC as an origin of the pronounced change in the spectral features of the sample in DAC N1 and to probe the electronic low‐energy excitations, we also measured the nuclear spin‐lattice relaxation time *T*_1_. The relaxation rate 1/*T*_1_ *T* of the samples in DACs N1 and N3 begins to decrease at temperatures above 260 K at 7 T (Figure 4a). A sharp drop in 1/*T*_1_ *T* by 3‐6 times in the temperature range between 220 and 260 K, not observed in the calibration samples and other DACs, indicates the superconducting nature of the transition in the lanthanum‐hydride samples in DACs N1 and N3. This confirms our findings based on the RF transmission studies discussed above (Figure 1).

For the DACs N1 and N3, the overall behavior of the ^1^H NMR spectra and the spin‐lattice relaxation time is very similar. This can be understood by the presence of the same *hP*‐LaH_12_ phase in both DACs (Figure 2).

Decompression of DACs N1 and N3 revealed that La‐sublattices in both phase modifications of LaH_12_ are very stable and decompose only at pressures about 20–40 GPa (Figure 4d; Figure S17). The hexagonal modification of LaH_12_ undergoes a sharp structural distortion with broadening of the diffraction peaks between 82 and 66 GPa. A comparison of the calculated equation of state *V(P)* and the experimental data (Figure 4d) also suggests that, when the pressure decreases below 140–150 GPa, the stoichiometry of the compounds may be lower than in XH_12_ and should be written as LaH_12‐x_ (x = 0…1).

## 3 Discussion

The complete volume displacement of an applied external magnetic field in the superconducting state is a key characteristic of type‐I superconductors. By contrast, in type‐II superconductors, in particular for granulated ones [25], the field penetrates the sample volume [80, 81, 82]. As the NMR signal intensity is proportional to the penetrated sample volume, this facilitates NMR measurements even at temperatures below *T*_c_ (Figure 4a,b). The measured spin‐lattice relaxation time of the sample in DAC N1 at 300 K is about 300 ms, and the product is *TT*_1_ ≈ 88 sK. Comparing the low‐temperature behavior of the spin‐lattice relaxation of the samples in DACs N1 and N2, we note that the differences in *TT*_1_ are small: *TT*_1_ = 573 sK at 50 K for the sample in DAC N1, and 340 sK at 40 K for the sample in DAC N2. This is not surprising, given the fact that superconducting phases yield only a very small contribution to the NMR signal at *T* << *T*_c_. The ^1^H signal at low temperatures is mainly due to the residual non‐superconducting hydrogen‐containing compounds.

As Figures 4a,b, and S20 show, there is a region of increased spin‐lattice relaxation rate between about 100 and 227 K for the sample in DAC N1, which can be associated with either LaH_12_ or residual NH_3_BH_3_. Phenomenologically, this feature in 1/*T*_1_ vs *T* resembles the relaxation behavior of ^27^Al NMR measurements, studied in the classical Hebel‐Slichter [38, 39] and Redfield‐Anderson [37] experiments (Figure S21). In this scenario, the hump below *T*_c_^50%^ for the sample in DAC N1 may be caused by the contribution of a Hebel‐Slichter‐type coherence peak, indicating the opening of the SC gap and the appearance of narrow maxima in the density of quasiparticle states *N*(*E*). Since the dependence 1/*T*_1_∝*N*(*E*)^2^ is quadratic, the presence of a maximum in *N*(*E*) leads to a peak in 1/*T*_1_ (the Hebel‐Slichter peak). If this increase of the relaxation rate is indeed related to such a coherence peak and not to a residual amount of NH_3_BH_3_ (Figure S9), it would be a strong argument in favor of conventional electron‐phonon pairing in lanthanum superhydrides [83, 84].

However, we would like to emphasize another observation, based on our *T_1_* data. In many superconductors, antiferromagnetic fluctuations [85], as well as a strong electron‐phonon interaction [86], may suppress a possible Hebel‐Slichter peak. LaH_12_ is expected to be a superconductor in the strong‐coupling limit. Indeed, describing the temperature dependence of 1/*T*_1_ below *T_c_* using an exponential fit allows to determine the superconducting gap of LaH_12_ at 165 GPa. As shown in Figure 4b (and Figure S20), such fits to the 1/*T*_1_ data for different temperature regimes provide an estimate of the superconducting gap Δ(0). We obtain values between about 427 and 671 K (36.8 and 57.8 meV), yielding *R*_Δ_ = 2Δ(0)/*k*_B_ *T*_c_ of about 3.76 to 5.16, in qualitative agreement with the strong electron‐phonon coupling extension of the BCS theory proposed by Migdal and Eliashberg [87, 88].

The onset temperature of the probable superconducting transition in LaH_12_ deserves a separate discussion. Although even in pure *Fm* $\bar{3}$ *m*‐LaH_10_ in zero field, the critical temperature *T*_c_^onset^ does not exceed about 250 K, in our experiment, we observe an unusual behavior of the NMR spectra and 1/*T*_1_ *T* right above 260 K at 7 T. Considering a typical value of the derivative *dB*_c2_ */dT* ≈ ‒ 1 T/K for superhydrides [89, 90], we estimate that at zero field, SC in the sample in DAC N1 sets in already around 267 K, which is significantly higher than the *T*_c_^onset^ observed in electrical‐transport measurements of LaH_10_ [2]. Thus, the results of our NMR study in magnetic fields of 4 and 7 T agree well with the RF transmission measurements in zero field (Figure 1).

Measurements of the electrical transport are limited to the current path of least resistance. By contrast, RF methods sense the full bulk of the sample. Hence, differences in *T*_c_^onset^ detected by these techniques are a natural consequence. Once SC sets in, the resistance drops to zero and any information below that transition is hidden. In inhomogeneous granular superconductors consisting of multiple phases with varying *T*_c_, a series‐connected normal‐metallic and superconducting resistance may be sensed. Hence, step‐like transitions with non‐zero resistances may be detected. Indeed, such features have been noted previously in La‐H [3, 50, 65] and La‐Sc‐H systems [26, 91] at temperatures much higher than 250 K. It is interesting to note that similar anomalies were also observed in the high‐frequency AC‐susceptibility measurements on LaH_10±_ *_x_* samples [51]. Although the aim of this work is not to provide a definitive proof of a near‐room temperature superconductivity in LaH_12_, but rather to demonstrate the feasibility of contactless research methods, ^1^H NMR and RF transmission, the data we obtained indicate the prospects for continuing the study of this exotic lanthanum polyhydride.

## 4 Conclusions

Employing ^1^H nuclear magnetic resonance spectroscopy and radio‐frequency transmission measurements, we studied five lanthanum‐hydride samples, including two crystal modifications of LaH_12_, LaH_9+_ *_x_*, tetragonal LaH_4_, and LaH_3_, at various pressures from 19 to 165 GPa in magnetic fields of 0, 4, and 7 T. The transition in the newly discovered LaH_12_ is characterized by a strong suppression of the ^1^H‐NMR signal intensity below about 260 K in a magnetic field of 7 T and may have a superconducting nature. Furthermore, it is accompanied by a pronounced change in all NMR characteristics, including the spin‐lattice relaxation rate 1/*T*_1_ *T*, which exponentially decreases at temperatures below *T*_c_. Our estimate of the superconducting gap in LaH_12_ gives Δ(0) of about 427 to 671 K (corresponding to 36.8 to 57.8 meV) and *R*_Δ_ = 2Δ(0)/*k*_B_ *T*_c_ of about 3.76 to 5.16, in accordance with the strong‐coupling scenario in lanthanum superhydrides.

We confirmed these observations by additional radio‐frequency transmission experiments on the same samples using a transformer configuration, where Lenz lenses act as primary and secondary coils. The transmission amplitude exhibits abrupt changes, already setting in at 267 ± 5 K (in zero field). Remarkably, the La‐H system contains phases with significantly higher *T*_c_ than what was previously reported based on electrical‐transport measurements. In the two samples in DACs N1 and N3, the detected changes in the ^1^H‐NMR spectral characteristics, and, most importantly, the spin‐lattice relaxation rate 1/*T*_1_ *T*, start about 15‐20 K above the zero‐field onset *T*_c_ of the canonical LaH_10_, determined by means of electrical‐transport measurements.

## Author Contributions

D.V.S., D.Z., and I.A.T. designed the experiments, prepared the DACs including sputtering of Lenz lenses and synthesis of the lanthanum hydride samples, did the COMSOL modelling, performed the RF transmission measurements, transport, powder X‐ray diffraction studies, and analyzed the XRD data. F.B., D.V.S., D.Z., and S.L. performed the NMR measurements and analyzed the NMR data. J.W., H.K., and T.H. supervised the work at HZDR. V.V.S. supervised the work at HPSTAR. All authors contributed to the discussion of the results and the preparation of the manuscript.

## Ethical Statement

There are no human subjects in this article and informed consent is not applicable.

## Conflicts of Interest

The authors declare no conflicts of interest.

## Supporting information




**Supporting File**: advs74245‐sup‐0001‐SuppMat.docx.

## Data Availability

The data that support the findings of this study are available from the corresponding author upon reasonable request.

## References

[1] A. P. Drozdov , M. I. Eremets , I. A. Troyan , V. Ksenofontov , and S. I. Shylin , “Conventional Superconductivity at 203 Kelvin at High Pressures in the Sulfur Hydride System,” Nature 525 (2015): 73–76, 10.1038/nature14964. 26280333

[2] A. P. Drozdov , P. P. Kong , V. S. Minkov , et al., “Superconductivity at 250 K in Lanthanum Hydride Under High Pressures,” Nature 569 (2019): 528–531, 10.1038/s41586-019-1201-8. 31118520

[3] M. Somayazulu , M. Ahart , A. K. Mishra , et al., “Evidence for Superconductivity Above 260 K in Lanthanum Superhydride at Megabar Pressures,” Physical Review Letters 122 (2019): 027001, 10.1103/PhysRevLett.122.027001. 30720326

[4] D. V. Semenok , A. G. Kvashnin , A. G. Ivanova , et al., “Superconductivity at 161 K in Thorium Hydride ThH_10_: Synthesis and Properties,” Materials Today 33 (2020): 36–44.

[5] P. Kong , V. S. Minkov , M. A. Kuzovnikov , et al., “Superconductivity up to 243 K in the Yttrium‐Hydrogen System Under High Pressure,” Nature Communications 12 (2021): 5075, 10.1038/s41467-021-25372-2. PMC8379216 34417471

[6] I. A. Troyan , D. V. Semenok , A. G. Kvashnin , et al., “Anomalous High‐Temperature Superconductivity in YH 6,” Advanced Materials 33 (2021): 2006832, 10.1002/adma.202006832. 33751670

[7] W. Chen , D. V. Semenok , X. Huang , et al., “High‐Temperature Superconducting Phases in Cerium Superhydride with a *T_c_ * up to 115 K Below a Pressure of 1 Megabar,” Physical Review Letters 127 (2021): 117001, 10.1103/PhysRevLett.127.117001. 34558917

[8] I. A. Troyan , D. V. Semenok , A. G. Ivanova , et al., “High‐Temperature Superconductivity in Hydrides,” Physics‐Uspekhi 65 (2022): 748–761, 10.3367/UFNe.2021.05.039187.

[9] D. Semenok , Computational Design of New Superconducting Materials and Their Targeted Experimental Synthesis (PhD Thesis, Skolkovo Institute of Science and Technology, 2022).

[10] N. W. Ashcroft , “Hydrogen Dominant Metallic Alloys: High Temperature Superconductors?,” Physical Review Letters 92 (2004): 187002, 10.1103/PhysRevLett.92.187002. 15169525

[11] F. Peng , Y. Sun , C. J. Pickard , R. J. Needs , Q. Wu , and Y. Ma , “Hydrogen Clathrate Structures in Rare Earth Hydrides at High Pressures: Possible Route to Room‐Temperature Superconductivity,” Physical Review Letters 119 (2017): 107001, 10.1103/PhysRevLett.119.107001. 28949166

[12] H. Liu , I. I. Naumov , R. Hoffmann , N. W. Ashcroft , and R. J. Hemley , “Potential High‐*T_c_ * Superconducting Lanthanum and Yttrium Hydrides at High Pressure,” Proceeding of the National Academy of Sciences 114 (2017): 6990–6995. 10.1073/pnas.1704505114 PMC5502634 28630301

[13] D. Duan , Y. Liu , F. Tian , et al., “Pressure‐Induced Metallization of Dense (H_2_S)_2_H_2_ with High‐*T_c_ * Superconductivity,” Scientific Reports 4 (2014): 6968, 10.1038/srep06968. 25382349 PMC4225546

[14] P. Giannozzi , S. Baroni , N. Bonini , et al., “QUANTUM ESPRESSO: A Modular and Open‐Source Software Project for Quantum Simulations of Materials,” Journal of Physics‐Condensed Matter 21 (2009): 395502. 10.1088/0953-8984/21/39/395502 21832390

[15] S. Baroni , S. D. Gironcoli , A. D. Corso , and P. Giannozzi , “Phonons and Related Crystal Properties from Density‐Functional Perturbation Theory,” Reviews of Modern Physics 73 (2001): 515–562, 10.1103/RevModPhys.73.515.

[16] G. Kresse and J. Furthmüller , “Efficient Iterative Schemes for Ab Initio Total‐Energy Calculations Using a Plane‐Wave Basis Set,” Physical Review B 54 (1996): 11169–11186, 10.1103/PhysRevB.54.11169. 9984901

[17] C. W. Glass , A. R. Oganov , and N. Hansen , “USPEX—Evolutionary Crystal Structure Prediction,” Computer Physics Communications 175 (2006): 713–720, 10.1016/j.cpc.2006.07.020.

[18] A. R. Oganov and C. W. Glass , “Crystal Structure Prediction Using *Ab Initio* Evolutionary Techniques: Principles and Applications,” Journal of Chemical Physics 124 (2006): 244704, 10.1063/1.2210932. 16821993

[19] Y. Wang , J. Lv , L. Zhu , and Y. Ma , “Crystal Structure Prediction via Particle‐Swarm Optimization,” Physical Review B 82 (2010): 094116, 10.1103/PhysRevB.82.094116.

[20] C. J. Pickard and R. J. Needs , “Ab Initio Random Structure Searching,” Journal of Physics‐Condensed Matter 23 (2011): 053201. 10.1088/0953-8984/23/5/053201 21406903

[21] M. Lüders , M. A. L. Marques , N. N. Lathiotakis , et al., “Ab Initio Theory of Superconductivity. I. Density Functional Formalism and Approximate Functionals,” Physical Review B 72 (2005): 024545.

[22] M. A. L. Marques , M. Lüders , N. N. Lathiotakis , et al., “ *Ab Initio* Theory of Superconductivity. II. Application to Elemental Metals,” Physical Review B 72 (2005): 024546, 10.1103/PhysRevB.72.024546.

[23] I. Errea , M. Calandra , and F. Mauri , “Anharmonic Free Energies and Phonon Dispersions from the Stochastic Self‐Consistent Harmonic Approximation: Application to Platinum and Palladium Hydrides,” Physical Review B 89 (2014): 064302, 10.1103/PhysRevB.89.064302.

[24] D. V. Semenok , I. A. Troyan , D. Zhou , W. Chen , H.‐K. Mao , and V. V. Struzhkin , “Observation of the Josephson Effect in Superhydrides: DC SQUID Based on (La,Ce)H_10+x_ with Operating Temperature of 179 K,” The Innovation Materials 3 (2025): 100115, 10.59717/j.xinn-mater.2024.100115.

[25] D. V. Semenok , A. V. Sadakov , D. Zhou , et al., “Superconducting Memory and Trapped Magnetic Flux in Ternary Lanthanum Polyhydrides,” Materials Today Physics 49 (2024): 101595, 10.1016/j.mtphys.2024.101595.

[26] D. V. Semenok , A. V. Sadakov , D. Zhou , O. A. Sobolevskiy , S. Luther , T. Helm , V. M. Pudalov , I. A. Troyan , and V. V. Struzhkin , Superconducting memory and trapped magnetic flux in ternary lanthanum polyhydrides. Materials Today Physics (2024). 49, 101595, 10.1016/j.mtphys.2024.101595.

[27] A. Aslandukova , A. Aslandukov , D. Laniel , et al., “Diverse High‐Pressure Chemistry in Y‐NH_3_BH_3_ and Y–Paraffin Oil Systems,” Science Advances 10 (2024): adl5416, 10.1126/sciadv.adl5416. PMC10936948 38478619

[28] D. Laniel , F. Trybel , B. Winkler , et al., “High‐Pressure Synthesis of Seven Lanthanum Hydrides with a Significant Variability of Hydrogen Content,” Nature Communications 13 (2022): 6987, 10.1038/s41467-022-34755-y. PMC9669027 36385117

[29] D. V. Semenok , B. L. Altshuler , and E. A. Yuzbashyan , “Fundamental Limits on the Electron‐Phonon Coupling and Superconducting T_c_ ,” Adv. Mater . 37, no. 40 (2025): 2507013, 10.1002/adma.202507013. 40685985

[30] T. Sakakibara , T. Goto , and N. Miura , “Contactless Transport Measurement of Metals in Pulsed High Magnetic Fields,” Review of Scientific Instruments 60 (1989): 444–449, 10.1063/1.1140398.

[31] Y. A. Timofeev , V. V. Struzhkin , R. J. Hemley , H.‐K. Mao , and E. A. Gregoryanz , “Improved Techniques for Measurement of Superconductivity in Diamond Anvil Cells by Magnetic Susceptibility,” Review of Scientific Instruments 73 (2002): 371–377, 10.1063/1.1431257.

[32] T. Meier , A. Aslandukova , F. Trybel , et al., “In Situ High‐Pressure Nuclear Magnetic Resonance Crystallography in One and Two Dimensions,” Matter and Radiation at Extremes 6 (2021): 068402.

[33] H. Günther , NMR Spectroscopy: Basic Principles, Concepts and Applications in Chemistry, 3rd ed. (John Wiley & Sons, 2013).

[34] I. I. Rabi , J. R. Zacharias , S. Millman , and P. Kusch , “A New Method of Measuring Nuclear Magnetic Moment,” Physical Review 53 (1938): 318, 10.1103/PhysRev.53.318. 1562763

[35] K. Müller and M. Geppi , Solid State NMR: Principles, Methods, and Applications (John Wiley & Sons, 2021).

[36] L. N. Cooper , “Microscopic Quantum Interference Effects in the Theory of Superconductivity,” Science 181, no. 4103 (1972): 908–916. 10.1126/science.181.4103.908 17835832

[37] A. G. Anderson and A. G. Redfield , “Nuclear Spin‐Lattice Relaxation in Metals,” Physical Review 116 (1959): 583–591, 10.1103/PhysRev.116.583.

[38] L. C. Hebel and C. P. Slichter , “Nuclear Relaxation in Superconducting Aluminum,” Physical Review 107 (1957): 901–902, 10.1103/PhysRev.107.901.

[39] L. C. Hebel and C. P. Slichter , “Nuclear Spin Relaxation in Normal and Superconducting Aluminum,” Physical Review 113 (1959): 1504–1519, 10.1103/PhysRev.113.1504.

[40] W. D. Knight , “Nuclear Magnetic Resonance Shift in Metals,” Physical Review 76 (1949): 1259–1260, 10.1103/PhysRev.76.1259.2.

[41] D. F. Smith and C. P. Slichter , “The Study of Mechanisms of Superconductivity by NMR Relaxation,” Lecture Notes in Physics 684 (2006): 243–295.

[42] J. Korringa , “Nuclear Magnetic Relaxation and Resonnance Line Shift in Metals,” Physica 16 (1950): 601–610, 10.1016/0031-8914(50)90105-4.

[43] T. Meier , N. Wang , D. Mager , J. G. Korvink , S. Petitgirard , and L. Dubrovinsky , “Magnetic Flux Tailoring Through Lenz Lenses for Ultrasmall Samples: A New Pathway to High‐Pressure Nuclear Magnetic Resonance,” Science Advances 3 (2017): aao5242, 10.1126/sciadv.aao5242. PMC5724354 29230436

[44] Y. Fu , R. Tao , L. Zhang , et al., “Trace Element Detection in Anhydrous Minerals by Micro‐Scale Quantitative Nuclear Magnetic Resonance Spectroscopy,” Nature Communications 15 (2024): 7293, 10.1038/s41467-024-51131-0. PMC11344839 39181900

[45] T. Meier , F. Trybel , S. Khandarkhaeva , et al., “Pressure‐Induced Hydrogen‐Hydrogen Interaction in Metallic FeH Revealed by NMR,” Physical Review X 9 (2019): 031008.

[46] T. Meier , D. Laniel , and F. Trybel , “Direct Hydrogen Quantification in High‐Pressure Metal Hydrides Matter and Radiation at Extremes Direct Hydrogen Quantification in High‐Pressure Metal Hydrides,” Matter and Radiation at Extremes 8 (2023): 18401.

[47] T. Meier , F. Trybel , G. Criniti , et al., “Proton Mobility in Metallic Copper Hydride from High‐Pressure Nuclear Magnetic Resonance,” Physical Review B 102 (2020): 165109, 10.1103/PhysRevB.102.165109.

[48] T. Meier , D. Laniel , M. Pena‐Alvarez , et al., “Nuclear Spin Coupling Crossover in Dense Molecular Hydrogen,” Nature Communications 11 (2020): 6334, 10.1038/s41467-020-19927-y. PMC7728769 33303751

[49] M. Yang , Y. Zhou , R. Jana , T. Nakagawa , Y. Fu , and T. Meier , “Hexagonal to Monoclinic Phase Transition in Dense Hydrogen Phase III Detected by High‐Pressure NMR,” arXiv (2024): 240719368.

[50] T. Shitaokoshi , S. Kawachi , T. Nomura , F. F. Balakirev , and Y. Kohama , “Radio Frequency Electrical Resistance Measurement Under Destructive Pulsed Magnetic Fields,” Review of Scientific Instruments 94 (2023): 094706. 37737700 10.1063/5.0165680

[51] N. Spengler , P. T. While , M. V. Meissner , U. Wallrabe , and J. G. Korvink , “Magnetic Lenz Lenses Improve the Limit‐of‐Detection in Nuclear Magnetic Resonance,” PLoS ONE 12 (2017): 0182779, 10.1371/journal.pone.0182779. PMC5557590 28813485

[52] D. Sun , V. S. Minkov , S. Mozaffari , et al., “High‐Temperature Superconductivity on the Verge of a Structural Instability in Lanthanum Superhydride,” Nature Communications 12 (2021): 6863, 10.1038/s41467-021-26706-w. PMC8617267 34824193

[53] A. P. Drozdov , V. S. Minkov , S. P. Besedin , et al., “Superconductivity at 215K in Lanthanum Hydride at High Pressures,” arXiv (2018): 180807039.

[54] Y. Chen , J. Wen , Z.‐X. He , et al., “Imaging Magnetic Flux Trapping in Lanthanum Hydride Using Diamond Quantum Sensors,” arXiv (2025): 251021877.

[55] P. Dalladay‐Simpson , G. Marchese , Z.‐Y. Cao , et al., “Evidence for a New Phase of Dense Hydrogen Above 325 Gigapascals,” Nature 529, no. 7584 (2025): 63–77. 10.1038/nature16164 26738591

[56] I. Errea , F. Belli , L. Monacelli , et al., “Quantum Crystal Structure in the 250‐Kelvin Superconducting Lanthanum Hydride,” Nature 578 (2020): 66–69, 10.1038/s41586-020-1955-z. 32025016

[57] I. A. Kruglov , D. V. Semenok , H. Song , et al., “Superconductivity of LaH_10_ and LaH_16_ Polyhydrides,” Physical Review B 101 (2020): 024508, 10.1103/PhysRevB.101.024508.

[58] D. V. Semenok , D. Zhou , J. Zhang , et al., “Radio‐Frequency Method for Detecting Superconductivity Under High Pressure,” arXiv (2025): 250900563.

[59] D. Semenok , I. Troyan , D. Zhou , et al., “Ternary Superhydrides Under Pressure of Anderson's Theorem: Near‐Record Superconductivity in (La,Sc)H_12_ ,” Advanced Functional Materials 35 (2025): 2504748, 10.1002/adfm.202504748.

[60] D. V. Semenok , D. Zhou , W. Chen , et al., “Stability and Superconductivity of Ternary Polyhydrides,” Annalen der Physik 538 (2025): 00467, 10.1002/andp.202500467.

[61] Z. Qiu , J. Chen , D. V. Semenok , et al., “Interlayer coupling enhanced superconductivity near 100K in La_3‐_ * _x_ *Nd* _x_ *Ni_2_O_7_ ,” arXiv (2025): 251012359.

[62] I. Kantor , V. Prakapenka , A. Kantor , et al., “BX90: A New Diamond Anvil Cell Design for X‐Ray Diffraction and Optical Measurements,” Review of Scientific Instruments 83 (2012): 125102, 10.1063/1.4768541. 23278021

[63] A. D. Grockowiak , M. Ahart , T. Helm , et al., “Hot Hydride Superconductivity Above 550 K,” Frontiers in Electronic Materials 2 (2022): 837651.

[64] V. Struzhkin , B. Li , C. Ji , et al., “Superconductivity in La and Y Hydrides: Remaining Questions to Experiment and Theory,” Matter and Radiation at Extremes 5 (2020): 028201, 10.1063/1.5128736.

[65] Y. L. Wu , X. H. Yu , J. Z. L. Hasaien , et al., “Ultrafast Dynamics Evidence of Strong Coupling Superconductivity in LaH_10±δ_ ,” Nature Communications 15 (2024): 9683, 10.1038/s41467-024-53103-w. PMC11549371 39516225

[66] Q. Jiang , D. Duan , H. Song , et al., “Prediction of Room‐Temperature Superconductivity in Quasi‐Atomic H_2_‐Type Hydrides at High Pressure,” Advanced Science 11 (2024): 2405561, 10.1002/advs.202405561. 39033541 PMC11425200

[67] C. F. Macrae , I. Sovago , S. J. Cottrell , et al., “Mercury 4.0: From Visualization to Analysis, Design and Prediction,” Journal of Applied Crystallography 53 (2020): 226–235, 10.1107/S1600576719014092. 32047413 PMC6998782

[68] R. Hrubiak , J. S. Smith , and G. Shen , “Multimode Scanning X‐Ray Diffraction Microscopy for Diamond Anvil Cell Experiments,” Review of Scientific Instruments 90 (2019): 025109, 10.1063/1.5057518. 30831723

[69] M. A. Kuzovnikov , “Composition Estimation of Novel Lanthanum Superhydrides,” a report presented at the 27th AIRAPT International Conference on High Pressure and Technology, Rio de Janeiro (Brazil), August 4–9, 2019.

[70] M. A. Kuzovnikov , A. P. Drozdov , and P. Kong , “Crystal Structures of Novel Lanthanum Superhydrides,” a report presented at 57th EHPRG Meeting on High Pressure Science and Technology, Prague, Czech Republic (2019).

[71] M. A. Kuzovnikov , “V(P) Equations of state of Novel Lanthanum and Yttrium Superhydrides,” presented at XXXVI Fortov International Conference on Interaction of Intense Energy Fluxes with Matter (ELBRUS 2021), V(P) Equations of state of Novel Lanthanum and Yttrium Superhydrides, ELBRUS (2021).

[72] D. V. Semenok , I. A. Troyan , А. G. Kvashnin , et al., “Superconductivity at 253 K in Lanthanum–Yttrium Ternary Hydrides,” Materials Today 48 (2021): 18–28, 10.1016/j.mattod.2021.03.025.

[73] A. H. Manayil Marathamkottil , K. Wang , N. P. Salke , et al., “X‐Ray‐Diffraction and Electrical‐Transport Imaging of Superconducting Superhydride (La,Y)H_10_ ,” Nature Communications 16 (2025): 11222, 10.1038/s41467-025-66262-1. PMC12714734 41413021

[74] D. Zhou , D. V. Semenok , D. Duan , et al., “Superconducting Praseodymium Superhydrides,” Science Advances 6 (2020): aax6849, 10.1126/sciadv.aax6849. PMC7048426 32158937

[75] J. Du , W. Sun , X. Li , and F. Peng , “Pressure‐Induced Stability and Superconductivity in LuH_12_ Polyhydrides,” Physical Chemistry Chemical Physics 25 (2023): 13320–13324, 10.1039/D3CP00604B. 37133917

[76] J. Bi , Y. Nakamoto , P. Zhang , et al., “Stabilization of Superconductive La–Y Alloy Superhydride with *T_c_ * Above 90 K at Megabar Pressure,” Materials Today Physics 28 (2022): 100840.

[77] W. Chen , X. Huang , D. V. Semenok , et al., “Enhancement of Superconducting Properties in the La–Ce–H System at Moderate Pressures,” Nature Communications 14 (2023): 2660, 10.1038/s41467-023-38254-6. PMC10170082 37160883

[78] O. Gunaydin‐Sen , R. Achey , N. S. Dalal , A. Stowe , and T. Autrey , “High Resolution ^15^N NMR of the 225 K Phase Transition of Ammonia Borane (NH_3_BH_3_): Mixed Order‐Disorder and Displacive Behavior,” Journal of Physical Chemistry B 111 (2007): 677–681, 10.1021/jp0649347. 17249810

[79] COMSOL Multiphysics v. 6.0 ., wwwcomsol.com. COMSOL AB, Stockholm, Sweden.

[80] A. A. Abrikosov , “The Magnetic Properties of Superconducting Alloys,” Journal of Physics and Chemistry of Solids 2 (1957): 199–208, 10.1016/0022-3697(57)90083-5.

[81] A. V. Sadakov , V. A. Vlasenko , I. A. Troyan , et al., “Vortex Phase Dynamics in Yttrium Superhydride YH_6_ at Megabar Pressures,” Journal of Physical Chemistry Letters 14 (2023): 6666–6671, 10.1021/acs.jpclett.3c01577. 37463103

[82] A. V. Sadakov , V. A. Vlasenko , D. V. Semenok , et al., “Quasi‐Two‐Dimensional Vortex Matter in the ThH_10_ Superhydride,” Physical Review B 109 (2024): 224515, 10.1103/PhysRevB.109.224515.

[83] V. S. Minkov , V. Ksenofontov , S. L. Bud'ko , E. F. Talantsev , and M. I. Eremets , “Magnetic Flux Trapping in Hydrogen‐Rich High‐Temperature Superconductors,” Nature Physics 19 (2023): 1293–1300, 10.1038/s41567-023-02089-1.

[84] M. I. Eremets , V. S. Minkov , A. P. Drozdov , and P. P. Kong , “The Characterization of Superconductivity Under High Pressure,” Nature Materials 23 (2024): 26–27, 10.1038/s41563-023-01769-w. 38172551

[85] D. C. Cavanagh and B. J. Powell , “Fate of the Hebel‐Slichter Peak in Superconductors with Strong Antiferromagnetic Fluctuations,” Physical Review Research 3 (2021): 013241, 10.1103/PhysRevResearch.3.013241.

[86] R. Akis and J. P. Carbotte , “Damping Effects on NMR in Superconductors,” Solid State Communications 78 (1991): 393–396, 10.1016/0038-1098(91)90691-N.

[87] A. B. Migdal , “Interaction Between Electrons and Lattice Vibrations in a Normal Metal,” Soviet Physics—Jetp 7 (1958): 996–1001.

[88] G. M. Eliashberg , “Interactions between Electrons and Lattice Vibrations in a Superconductor,” Soviet Physics—Jetp 11 (1960): 696–709.

[89] D. Semenok , J. Guo , D. Zhou , et al., “Evidence for Pseudogap Phase in Cerium Superhydrides: CeH_10_ and CeH9,” arXiv (2023): 230711742v2.

[90] D. V. Semenok , I. A. Troyan , A. V. Sadakov , et al., “Effect of Magnetic Impurities on Superconductivity in LaH_10_ ,” Advanced Materials 34 (2022): 2204038, 10.1002/adma.202204038. 35829689

[91] Y. Song , C. Ma , H. Wang , et al., “Room‐Temperature Superconductivity at 298 K in Ternary La‐Sc‐H System at High‐Pressure Conditions,” arXiv (2025): 251001273.

