# Supplementary material for: Transmission of Radio‐Frequency Waves and Nuclear Magnetic Resonance in Lanthanum Superhydrides
Source: Adv Sci (Weinh). 2026 Feb 8;13(23):e20701. doi: 10.1002/advs.202520701 (PMC13104150; doi:10.1002/advs.202520701)
Supplement: Supplementary file 1 — Supporting File: advs74245‐sup‐0001‐SuppMat.docx. [file ADVS-13-e20701-s001.docx]

**Supplementary Information**

**Transmission of radio-frequency waves and nuclear magnetic resonance in lanthanum superhydrides**

*Dmitrii V. Semenok ^†,*^, Florian Bärtl ^†^, Di Zhou ^†^, Toni Helm, Sven Luther_,_ J. Wosnitza, Ivan A. Troyan, Viktor V. Struzhkin ^*^_,_ and Hannes Kühne ^*^*

* Corresponding authors, emails: dmitrii.semenok@hpstar.ac.cn (Dmitrii V. Semenok), viktor.struzhkin@hpstar.ac.cn (Viktor V. Struzhkin), h.kuehne@hzdr.de (Hannes Kühne).

^†^These authors contributed equally.

**Content**

[1. Materials and Methods S2](file:///E:\MINE\Own%20papers\NMR_La-H\Draft\Science\supplementary_materials_HF_and_NMR_in_LaH12.docx#_Toc192336166)

[2. Preparation of Lenz lenses S5](file:///E:\MINE\Own%20papers\NMR_La-H\Draft\Science\supplementary_materials_HF_and_NMR_in_LaH12.docx#_Toc192336167)

[3. Radio-frequency transmission measurements S6](file:///E:\MINE\Own%20papers\NMR_La-H\Draft\Science\supplementary_materials_HF_and_NMR_in_LaH12.docx#_Toc192336168)

[4. NMR experiments S10](file:///E:\MINE\Own%20papers\NMR_La-H\Draft\Science\supplementary_materials_HF_and_NMR_in_LaH12.docx#_Toc192336169)

[5. COMSOL modeling S15](file:///E:\MINE\Own%20papers\NMR_La-H\Draft\Science\supplementary_materials_HF_and_NMR_in_LaH12.docx#_Toc192336170)

[6. X-ray diffraction data S17](file:///E:\MINE\Own%20papers\NMR_La-H\Draft\Science\supplementary_materials_HF_and_NMR_in_LaH12.docx#_Toc192336171)

[7. Raman spectroscopy S21](file:///E:\MINE\Own%20papers\NMR_La-H\Draft\Science\supplementary_materials_HF_and_NMR_in_LaH12.docx#_Toc192336172)

[8. Additional NMR data S22](file:///E:\MINE\Own%20papers\NMR_La-H\Draft\Science\supplementary_materials_HF_and_NMR_in_LaH12.docx#_Toc192336173)

**1. Materials and Methods**

*1. Diamond-anvil cells*

To prepare the DACs N1 and N2, 300 μm thick tungsten gaskets were used. These were compressed to a pressure of 20 GPa before drilling holes that were 50-100 % of the diameter of the diamond anvil culet. After drilling the holes, the surface of the tungsten gaskets was coated on both sides with a 1-2 μm thick layer of tantalum, using magnetron. We then heated the gaskets in air to 1000 °C to oxidize Ta and form Ta_2_O_5_. The resulting W/Ta/Ta_2_O_5_ insulating gasket with a layer of Ta_2_O_5_ (contact resistance is above 6 MΩ) is reliably insulated tungsten gasket from Lenz lenses deposited on the diamond anvils. DACs N3 and N4 were prepared using rhenium (Re, 300 μm thick) gaskets with a sputtered insulating layer of ~50 nm thick aluminum oxide. The aluminum oxide was prepared by DC magnetron sputtering of aluminum in an oxygen atmosphere.

For the DACs N1-N4, Lenz lenses made of deposited Cu/Mo and Cu layers with a thickness of 0.6-2 μm were fabricated. Magnetron sputtering was carried out in DC mode. The total thickness of the metal layer was 600-755 nm (Fig. S1). The width of the ion etching zones separating the conducting rings of the Lenz lenses was about 5 μm. Three-stage Lenz lenses, with a diameter of the annular etching zones of 60 μm, 300 μm and 3 mm (Fig. S1c) were used.

**Table S1.** Parameters of samples, high-pressure DACs, Lenz lenses, gaskets and diamond anvils used in this study. “sc” means simple cubic space group (*Pm*$\bar{3}$*m*), “h” means “hexagonal”. WC – is the nonmagnetic tungsten carbide, and c-BN is the cubic boron nitride.

|  | **DAC N0** | **DAC N1** | **DAC N2** | **DAC N3** | **DAC N4** |
| --- | --- | --- | --- | --- | --- |
| **Starting material** | La/AB | La/AB | La/AB | LaH_3-x_/AB | LaH_3-x_/AB |
| **DAC’s material** | BeCu | 40HNU | BeCu | BeCu | BeCu |
| **PCB material** | teflon | teflon | teflon | textolite | textolite |
| **Gasket** | nonmagnetic steel/MgO | W/Ta/Ta_2_O_5_ | W/Ta/Ta_2_O_5_ | Re/Al_2_O_3_ | Re/Al_2_O_3_ |
| **Culet diameter, μm** | 80 | 75 | 100 | 100 | 100 |
| **Glue** | Epoxy | Na_2_SiO_3_×D_2_O | Na_2_SiO_3_×D_2_O | Superglue | Superglue |
| **Lenz lens** | Ta/Au, physical mask | Cu / Ga FIB | Cu/ lithography | Cu/Mo, Ga FIB | Cu, Ga FIB |
| **Seat material** | Non-magnetic WC | Non-magnetic WC | Non-magnetic  c-BN | Non-magnetic WC | Non-magnetic WC |
| **Pressure, GPa** | 149-120 | 165 | 147-150 | 80-90 | 19 |
| **Composition according to XRD** | N/A | h-LaH_12_ | LaH_3_ + LaH_4_ | sc-LaH_12_ + h-LaH_12_ | LaH_2_+LaH_3_ |
| **Signal/Noise ratio in non-SC state (number of scans)** | N/A | 270 (10^4^) | 144 (2‧10^3^) | 560 (2‧10^3^) | 15 (1.3‧10^5^) |

The electrical circuit of all DACs included an RF emitter in the form of a single-turn coil on a 0.3 mm thick printed circuit board (PCB) made out of textolite or Teflon, 0.1 mm thick Cu or Au conductive patterns with a diameter of 3-4 mm, depending on the diameter of the diamond anvil. The RF coil was equipped with a 5 nF ceramic matching capacitor. The RF emitters were connected in series to form the contour of a two-turn coil. Organic glue was used in minimal quantities and as far away from electrical contacts as possible.

*2. LaH_x_ samples*

The samples in DAC N0, N1 and N2 were synthesized using laser heating of metallic La microparticles and NH_3_BH_3_, while the samples in N3 and N4 were obtained from LaH_3-x_ and NH_3_BH_3_. The infrared (1.04 µm) laser heating under high pressure consisted of a series of 10-20 pulses with a duration of 0.1-0.3 seconds. Temperature was not measured during laser heating.

*3. Nuclear magnetic resonance*

The ^1^H NMR experiments were conducted in a superconducting magnet, which reaches magnetic fields up to 8 T. The radial homogeneity of the field is better than 2.5 ppm over 10 mm, whereas the longitudinal field varies less than 4 ppm within ± 5 mm from the center of the field. The magnet is equipped with a ^4^He flow cryostat, which can reach temperatures from 1.6 to 300 K, with temperature stabilization of much better than 1 %. All NMR data were recorded using a commercial, phase-coherent spectrometer with a maximum frequency of 500 MHz, and an RF power amplifier, achieving a pulse power of up to 250 W. Standard Hahn-spin-echo sequences were used to measure the ^1^H NMR spectra. The pulse sequences were typically spaced apart by delay times of typically 500 ms and we usually accumulated 2000 scans for a ^1^H spectral signal-to-noise ratio between 80 and 300. To process the NMR data, we used a home-built LabView software and the MestReNova program ^[92]^.

In order to obtain a very stable resonance frequency of the NMR-resonant circuit, we chose a top-tuned configuration, where we placed the capacitors of the resonant circuit outside of the cryostat and operated them with stepper motors for automated tuning and matching. Additionally, we used a phase shifter to achieve a wide tuning range of the resonance frequency. This setup enables a broadband frequency tuning of the resonant circuit and guarantees stable resonance conditions for the whole temperature range.

The spin-lattice-relaxation rate was measured using a saturation-recovery sequence (π/2 🡪 τ’ 🡪 π/2 🡪 τ 🡪 π). For short values of τ’, the NMR signal intensity (= Mz) is close to zero, whereas for long values of τ’, the intensity saturates. We evaluated the resulting relaxation curve by fitting a single-exponential function for spin-1/2 nuclei to our data according to

$$M_{z}\left( \tau\right)=M_{z0}\left[ 1-e^{-\left( \frac{\tau^{'}}{T_{1}} \right)^{\beta}} \right]+offset, (S1)$$

where $M_{z0}$ is the equilibrium nuclear magnetization, $offset$ is a parameter that accounts for non-perfect nutation conditions during the NMR pulse sequence (and is very small compared to the contribution of the exponential term in our experiments). The stretching exponent 0 < *β* ≤ 1 is a parameter that reflects the distribution width of *T*_1_. If *β* = 1, every excited nuclear spin relaxes with the same *T*_1_. For smaller *β*, the underlying distribution function of *T*_1_ broadens.

*4. Radio-frequency transmission*

Measurements of the radio-frequency transmission were performed through the Lenz-lens system in the DACs in a circuit with two lock-in amplifiers (Fig. S2). An SR844 lock-in amplifier (Stanford Research) was used as high-frequency signal generator, as well as a receiver to measure the transmitted signal at the same frequency. The sample was placed in a low-frequency magnetic field (19 Hz or 33 Hz, maximum induction between 20 and 40 Gauss), generated by a solenoid. The current feeding the solenoid was created by an SR830 lock-in amplifier, working in tandem with a Yamaha PX3 power amplifier. The current amplitude in the solenoid circuit was typically 0.5‒5 A. The envelope of the high-frequency signal near the superconducting transition temperature contains even harmonics, of which the strongest, the 2^nd^ harmonic, was detected.

The warming and cooling rates in our experiments with DAC N1 were about ‒5 K/min for cooling, and +0.8-0.9 K/min for the warming cycle near 270 K. This difference in the scanning rate may lead to a certain temperature hysteresis in the heating and cooling cycles, which can be seen in Figure 1f. Overall, the warming cycle was much slower and the temperature indicated by the thermometer (attached to the high-pressure DAC) was much closer to the sample temperature.

*5. X-ray diffraction*

The ambient temperature X-ray diffraction measurements were performed using the synchrotron X-ray diffraction on the ID27 beamline with a beam of wavelength of 0.3738 Å at the European Synchrotron Research Facility (ESRF, proposal MA-5924).

Mapping of the sample center was carried out on a 10×10 and 4×4 grids with a step of 5 microns, accumulating time was about 10 seconds per image. The experimental XRD images were integrated and analyzed using the Dioptas 0.6 software package ^[93]^. To fit the diffraction patterns and obtain the cell parameter, we analyzed the data using Mercury 2021.2.0 ^[67]^ and Jana2006 software ^[94]^, employing the Le Bail method ^[95]^. Analysis of the spatial distribution of hydride phases in the sample was performed using the XDI ^[68]^ and Dioptas 0.6 programs.

*6. SEM/FIB*

SEM images and FIB assisted lithography for the fabrication of Lenz lenses were done with the help of Helios DualBeam systems (Xenon plasma and Gallium ions) from ThermoFisher Scientific.

**2. Preparation of Lenz lenses**


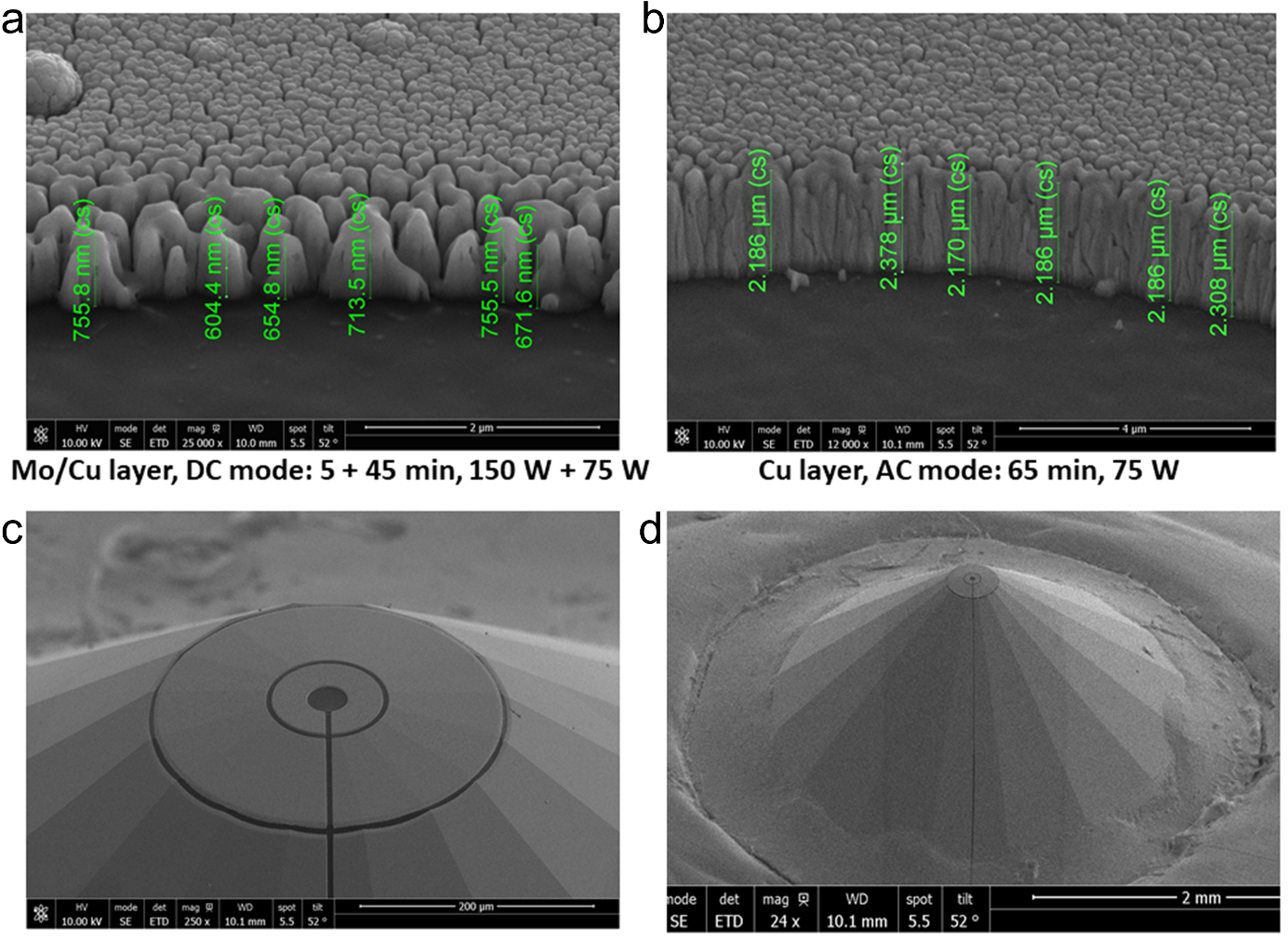


**Fig. S1.** Scanning electron microscopy of a typical result of magnetron sputtering followed by ion etching of Lenz lenses on a diamond anvil. (a) Determination of the thickness of the two-layer Mo/Cu coating. (b) Determination of the thickness of the sputtered pure copper film. Sputtering pure copper was carried out in alternating-current mode for 65 minutes at a power source of 75 W. The layer thickness was more than 2 microns. (c) Electron microscopy of a diamond anvil with a sputtered Lenz lens. (d) Continuation of the radial cut to the boundaries of the sprayed metal layer.

**3. Radio-frequency transmission measurements**

We performed measurements of the transmission of high-frequency signals through the Lenz-lens system in the DACs in a circuit with two lock-in amplifiers (Fig. S2). We used a SR844 lock-in amplifier (Stanford Research) as high-frequency signal generator, as well as receiver to measure the transmitted signal at the same frequency. We placed the sample in a low-frequency magnetic field (19 Hz or 33 Hz, maximum induction between 20 and 40 Gauss), generated by a special solenoid. The current feeding the solenoid was created by an SR830 lock-in amplifier, working in tandem with a Yamaha PX3 power amplifier. The current amplitude in the solenoid circuit is typically 0.5‒5 A. The envelope of the high-frequency signal near the superconducting transition temperature contains even harmonics, of which the strongest, the 2^nd^ harmonic, was detected, thus serving as an additional marker of the superconducting transition. In high-frequency measurements, the target signal can have two basic forms: (1) A peak, hump, or trough of various shapes; (2) a step. Typical measurement parameters are given in Table S2.

**
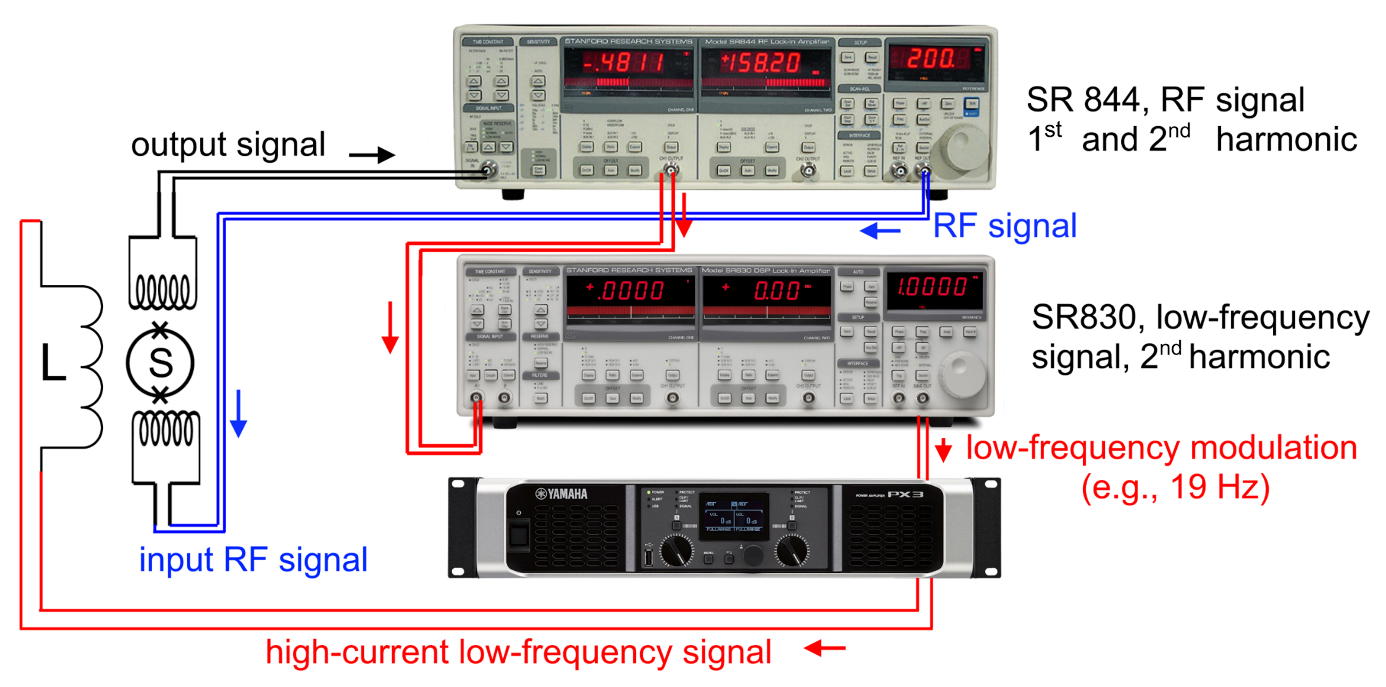
**

**Fig. S2.** Scheme of the setup for radio-frequency transmission studies of the samples in the DACs. “L” represents an external solenoid creating low-frequency modulating magnetic field (usually 19 or 33 Hz). “S” stands for sample.

**Table S2.** Parameter settings for the SR844 and SR830 lock-in amplifiers used in our RF measurements.

|  | **SR844** | **SR830** |
| --- | --- | --- |
| **Time constant** | 100 µs | 300 ms |
| **Filter** | 18 or 24 dB | 18 or 24 dB |
| **Signal input** | 50 Ω (without capacitors)  30 pF (with capacitors) | AC |
| **Wide Reserve** | Low noise or Normal | Low noise or Normal |
| **Harm #** | 1 or 2 | 2, 4, 6 … |


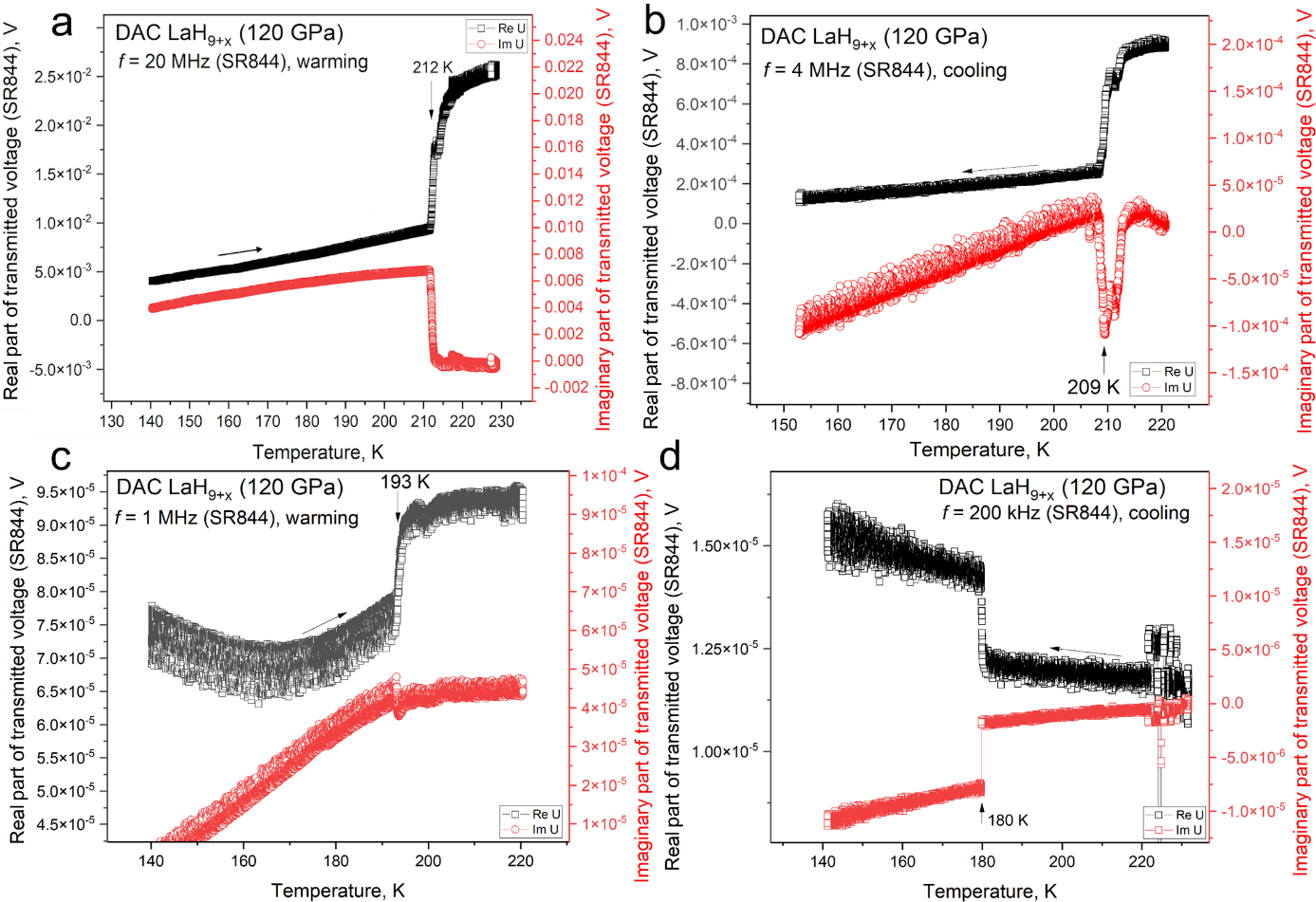


**Fig. S3.** Radio-frequency transmission through the sample in DAC N0 (LaH_9+x_) at 120 GPa at different carrier frequencies *f* in cooling and warming cycles. One of the van der Pauw electrode pairs (see Fig. 1 in the main test) was used as an emitting antenna. Real and imaginary components of the transmitted signal at: (a) *f* = 20 MHz. The superconducting transition begins at about 212 K; (b) *f* = 4 MHz; (c) *f* = 1 MHz; (d) *f* = 200 kHz. As the frequency decreases, the hydride phase that is most abundant in the sample has the greatest influence on the RF transmittance.

Features of the passage of radio-frequency electromagnetic waves through a sample can serve as a marker of superconductivity. At the moment of superconducting transition, undamped eddy currents arise on the sample surface, which change the amplitude and phase of the passing RF signal, screening and dissipating it. In addition, the movement of the Abrikosov vortices at the stage of vortex liquid leads to noticeable dissipation peak of the RF waves.

As can be seen in Fig. S4 and Fig. S5, the features of the radio-frequency transmission and of the second harmonic of the low-frequency magnetic field passing through the sample in DAC N1 are concentrated in two temperature intervals: (1) 248-252 K (Fig. S5), which corresponds well to the superconducting properties of cubic LaH_10_ at 165 GPa^[2]^; (2) the region of 267-286 K (Fig. S4), where the signal anomalies are more pronounced. This temperature region corresponds to the onset of the superconducting transition in a previously unknown lanthanide-hydride phase, which dominates the sample, probably *hP*-LaH_12_. It is important to note that resistive transitions at such high temperatures (265-280 К) have been observed in the La-H system by various authors since 2019 ^[3,50,51]^.

The superconducting state in the vicinity of the critical temperature is sensitive to the application of even a weak oscillating (ν) magnetic field. Periodic suppression of the superconducting state leads to modulation of the radio-frequency transmission and the appearance of a second low-frequency harmonic (2ν), which can be observed in our experiments in Fig. 4b,c.


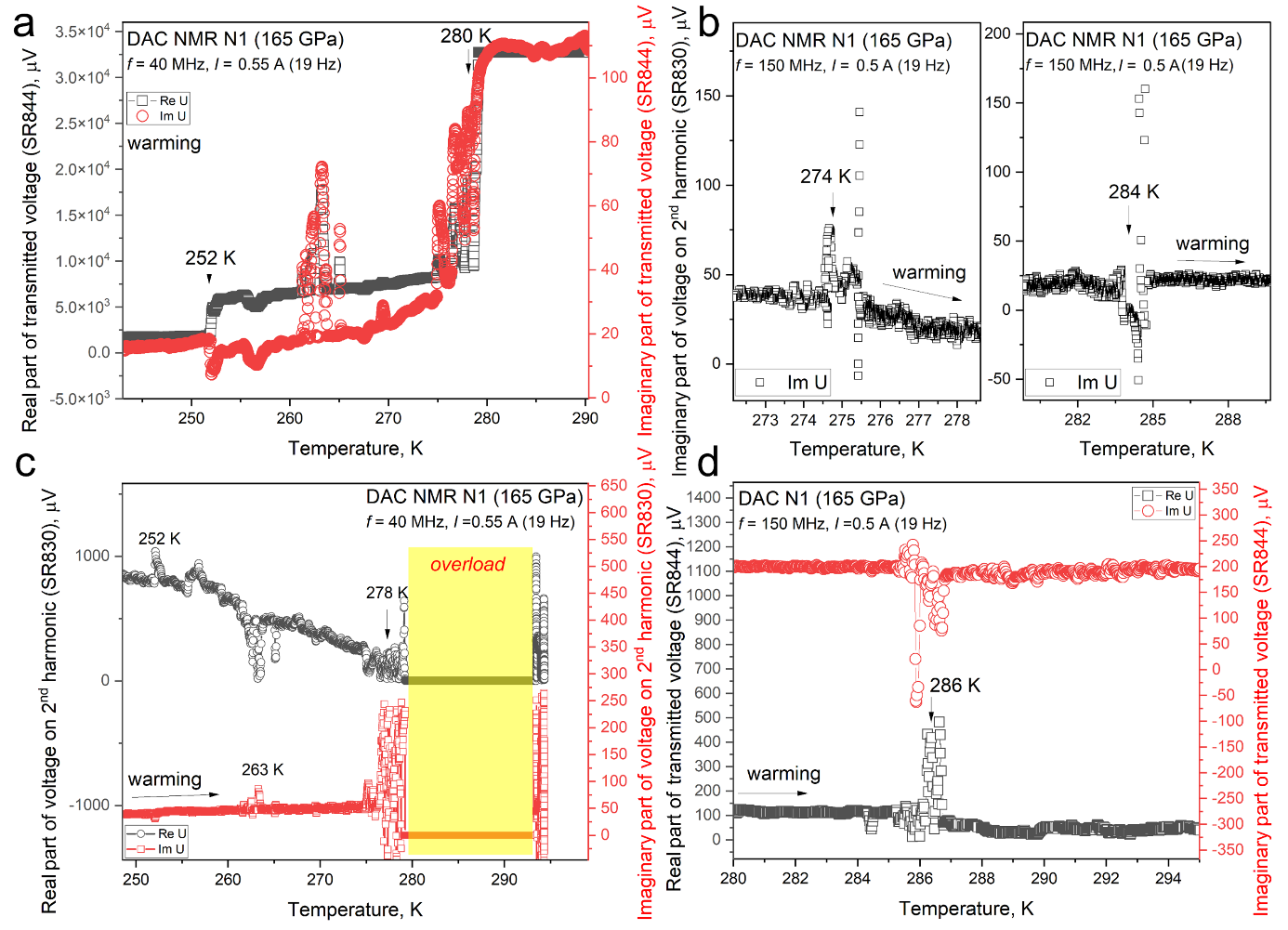


**Fig. S4.** Radio-frequency transmission through the sample in DAC N1 (NMR) at 165 GPa at different carrier frequencies *f* and currents *I* = 0.5-0.55 A through the external solenoid. (a) Real and imaginary components of the transmitted signal at 40 MHz with a main transition at *T_c_* ≈ 275-280 K. The small step at 252 K is likely related to superconductivity of *Fm*$\bar{3}$*m*-LaH_10_. (b) Imaginary part of the second-harmonic signal of the low-frequency modulating field (19 Hz) with features around 274 (run 1) and 284 K (run 2). Carrier frequency is 150 MHz. (c) Real and imaginary components of the second-harmonic signal of the low-frequency modulating field (19 Hz). Carrier frequency is 40 MHz. (d) Real and imaginary components of the transmitted signal at 150 MHz (run 2).

**
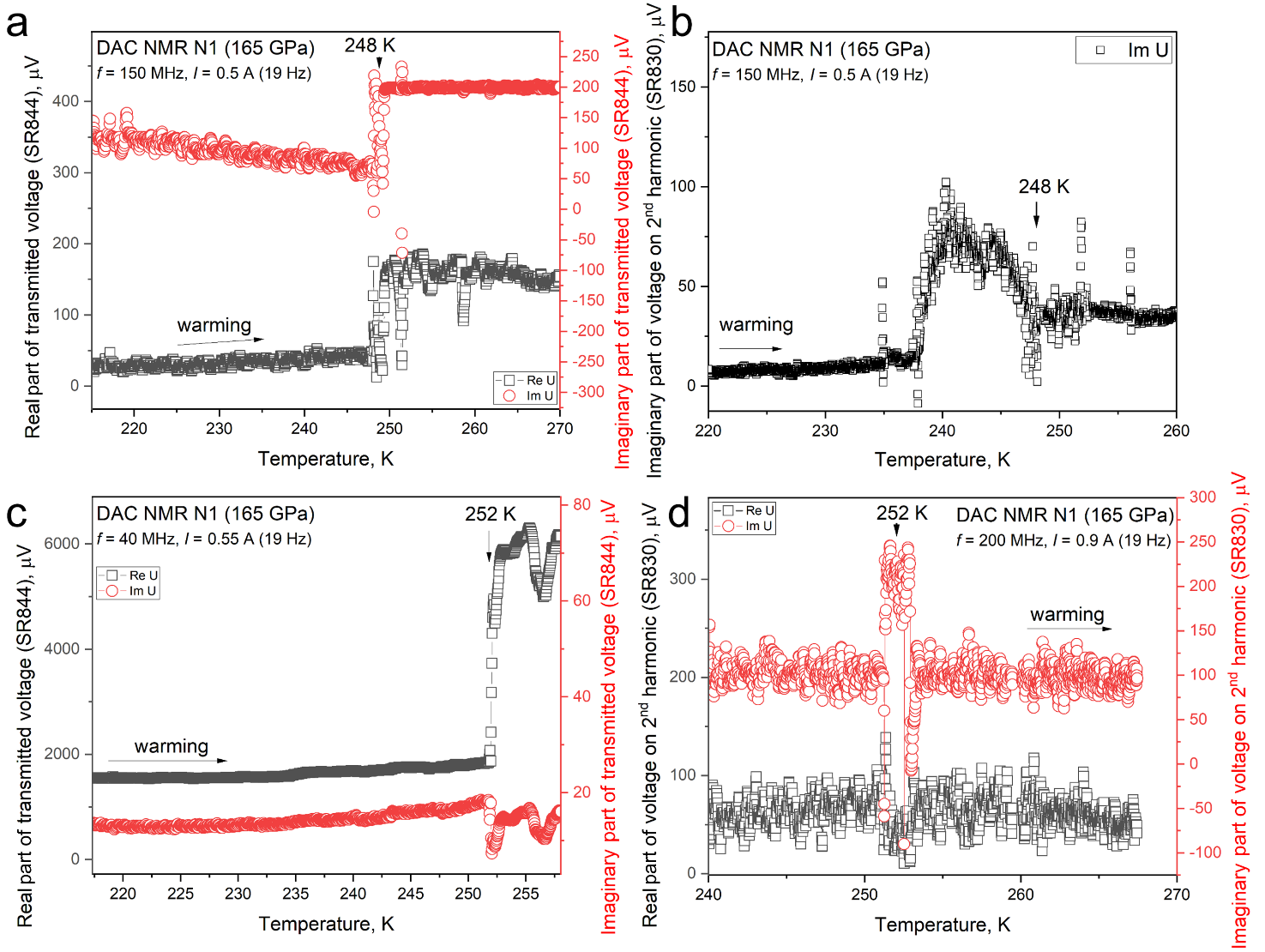
**
**Fig. S5.** Radio-frequency transmission through the sample in DAC N1 (NMR) at 165 GPa at different carrier frequencies *f* around 250 K, far from the region of the high-temperature superconducting transition (260-280 K). (a) Real and imaginary components of the transmitted signal at 150 MHz, with a transition probably corresponding to *Fm*$\bar{3}$*m*-LaH_10_. (b) Signal of the second harmonic of the low-frequency modulating field (19 Hz). The onset of the signal at 248 K corresponds to superconductivity in *Fm*$\bar{3}$*m*-LaH_10_. (c) Real and imaginary components of the transmitted signal at 40 MHz. (d) Signal of the second harmonic of the low-frequency modulating field (19 Hz) at a carrier frequency of 200 MHz. The pronounced peak probably corresponds to superconductivity in *Fm*$\bar{3}$*m*-LaH_10_.

**4. NMR experiments**

In order to validate our NMR measurements, shown in Fig. 3 and 4 of the main text, in particular to demonstrate that we can indeed detect an NMR signal from inside the DAC, we conducted test measurements of a DAC loaded with ammonia borane (AB or NH3BH3) and an aluminum (27Al) foil. In Fig. S6c, we show the integrated spectral intensity as function of the interpulse delay time (τ) of a saturation-recovery T1 measurement at 10 K and compare it to a measurement of pure ^27^Al powder, measured in a separate, conventional NMR experiment. We determine *T*_1_ and the stretching exponent *β* the same way as described in section 1 (S1). The *T*_1_ value of the conventional NMR measurement in the 3He system (182 ms) agrees well with the established Korringa value of ^27^Al (1.85 sK). Our sample in the DAC yields a *T*_1_ value that is three times larger (574 ms) by comparison. The reason for this is a non-uniform spatial distribution of the radio-frequency field across the Al foil (stemming from a non-ideal Lenz lens geometry in this experiment), which is also indicated by the low stretching exponent of 0.4.

**
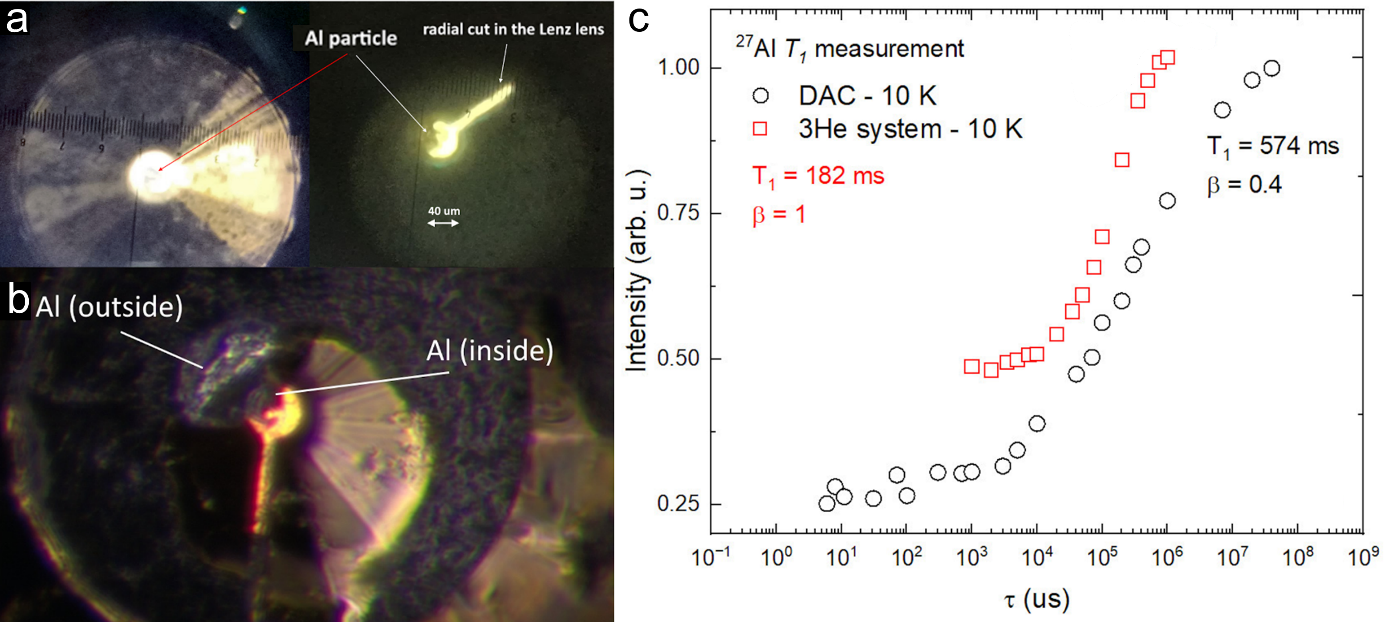
**

**Fig. S6.** Calibration ^27^Al NMR spin-echo data using a test DAC loaded with ammonia borane (AB or NH_3_BH_3_), and an aluminum (^27^Al) foil, equipped with two sputtered 0.5 µm thick gold Lenz lenses. (a) Pictures of the DACs culet in reflected and transmitted light with the space filled with AB and aluminum foil. (b) Picture of the opposite anvil of the test DAC, with the Al foil in and outside the gasket. (c) Integrated intensity of the ^27^Al NMR signal as function of the spin-echo delay time (τ) at 10 K.


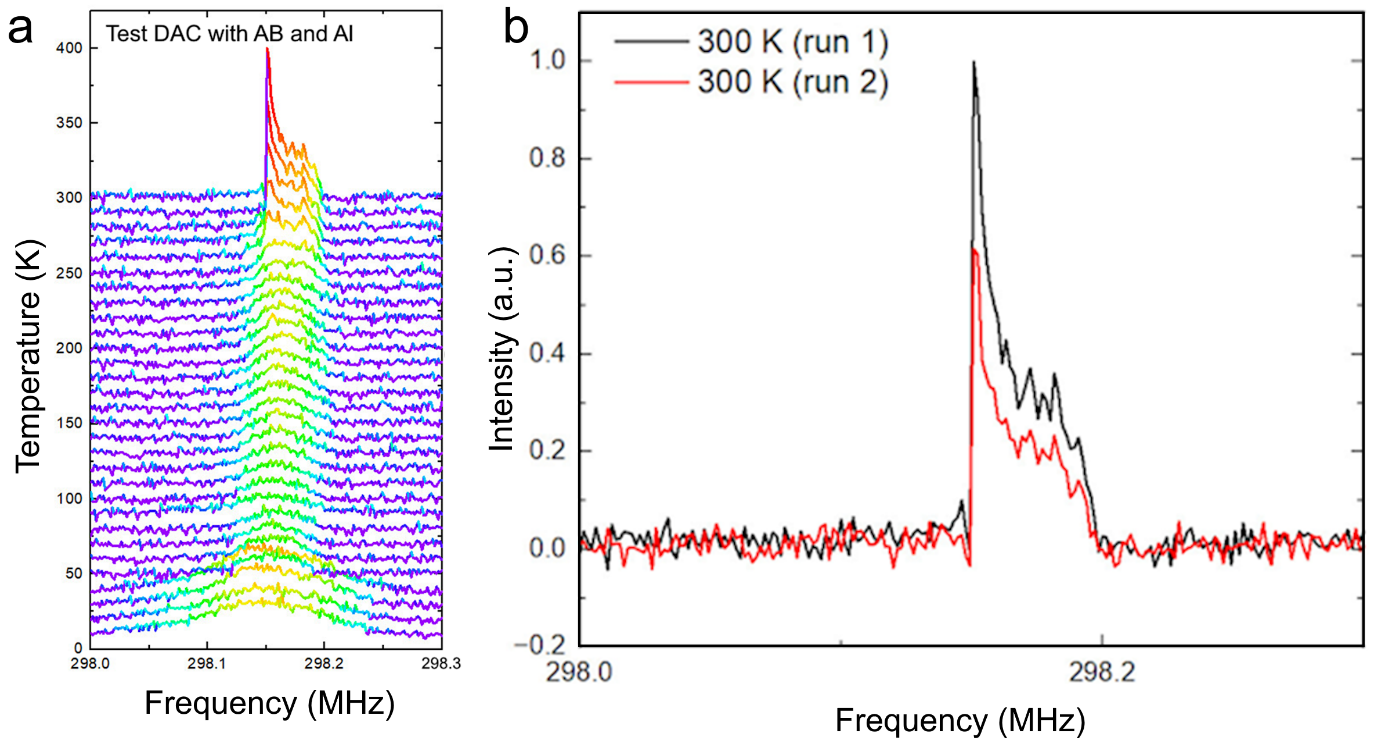


**Fig. S7.** Calibration ^1^H NMR data using a test DAC and loaded with ammonia borane (AB or NH_3_BH_3_), and an aluminum (^27^Al) foil, equipped with two sputtered 0.5 µm thick gold Lenz lenses. (a) Stack of ^1^H NMR spectra at different temperatures during cooling from 300 K to 10 K. The signal is significantly stronger than in the case of an empty DAC. (b) ^1^H NMR spectra at 300 K before (run 1) and after cooling to 10 K and heating back to 300 K (run 2). The signal intensity is only slightly reduced due to the removal of volatile H-containing compounds from the DAC.

Fig. S7 shows the ^1^H NMR measurements of the DAC, loaded with NH­_3_BH_3_ and a piece of aluminum foil. The temperature dependence of the spectra is similar to the spectra measured in a conventional setup, shown in Fig. S9. In contrast to the spectra of the empty DAC (Fig. S8), the signal does not vanish suddenly below 230 K (Fig. S7a). Importantly, after the initial cooldown to 10 K, the NMR line is almost unchanged, except for a very small loss in intensity, which we attribute to the removal of volatile ^1^H in the sample space.

**
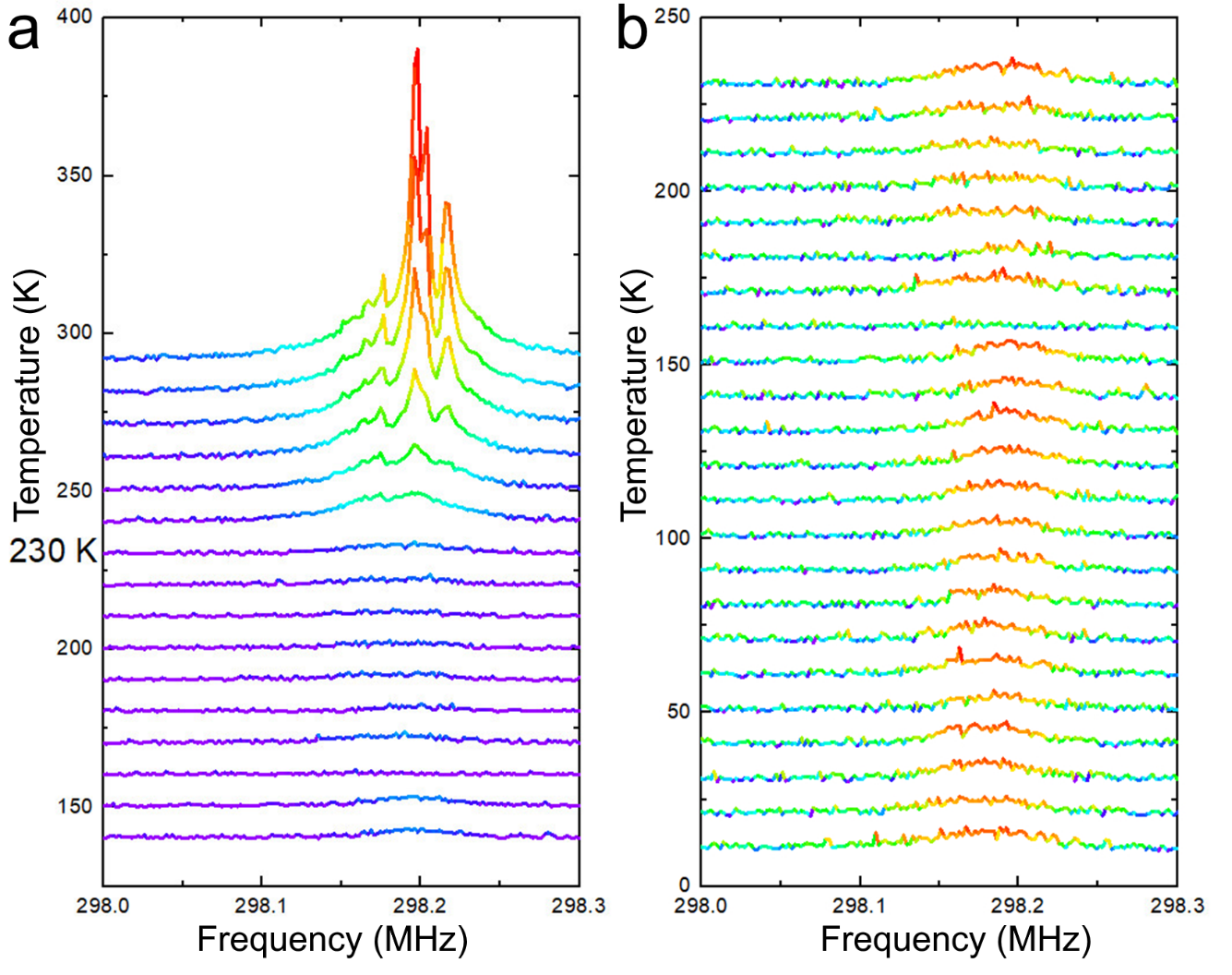
**

**Fig. S8.** Calibration ^1^H NMR data using an empty DAC equipped with two sputtered 0.5 µm thick gold Lenz lenses. The single-turn excitation coils for this DAC were printed on a ~150 µm thick teflon PCB. (a) Stack of ^1^H NMR spectra at different temperatures during cooling from 300 K to 140 K, and (b) from 230 K to 10 K.

Fig. S8 shows ^1^H NMR measurements of an empty DAC. At temperatures above 230 K, we observe a strong ^1^H signal, which quickly disappears below this temperature. In the low-temperature regime, the signal is very weak and does not show a temperature dependence. Moreover, we observed that the signal intensity did not increase again after we heated the sample back to 300 K. We interpret this behavior as contributions of volatile Hydrogen in the sample space, which disappears during the cooldown. We therefore changed our measurement protocol to first cool our sample below 200 K and then start the measurements. Importantly, this is the only DAC, which shows this behavior. All other DACs showed reproducible spectral intensities (compare Fig. S7b).


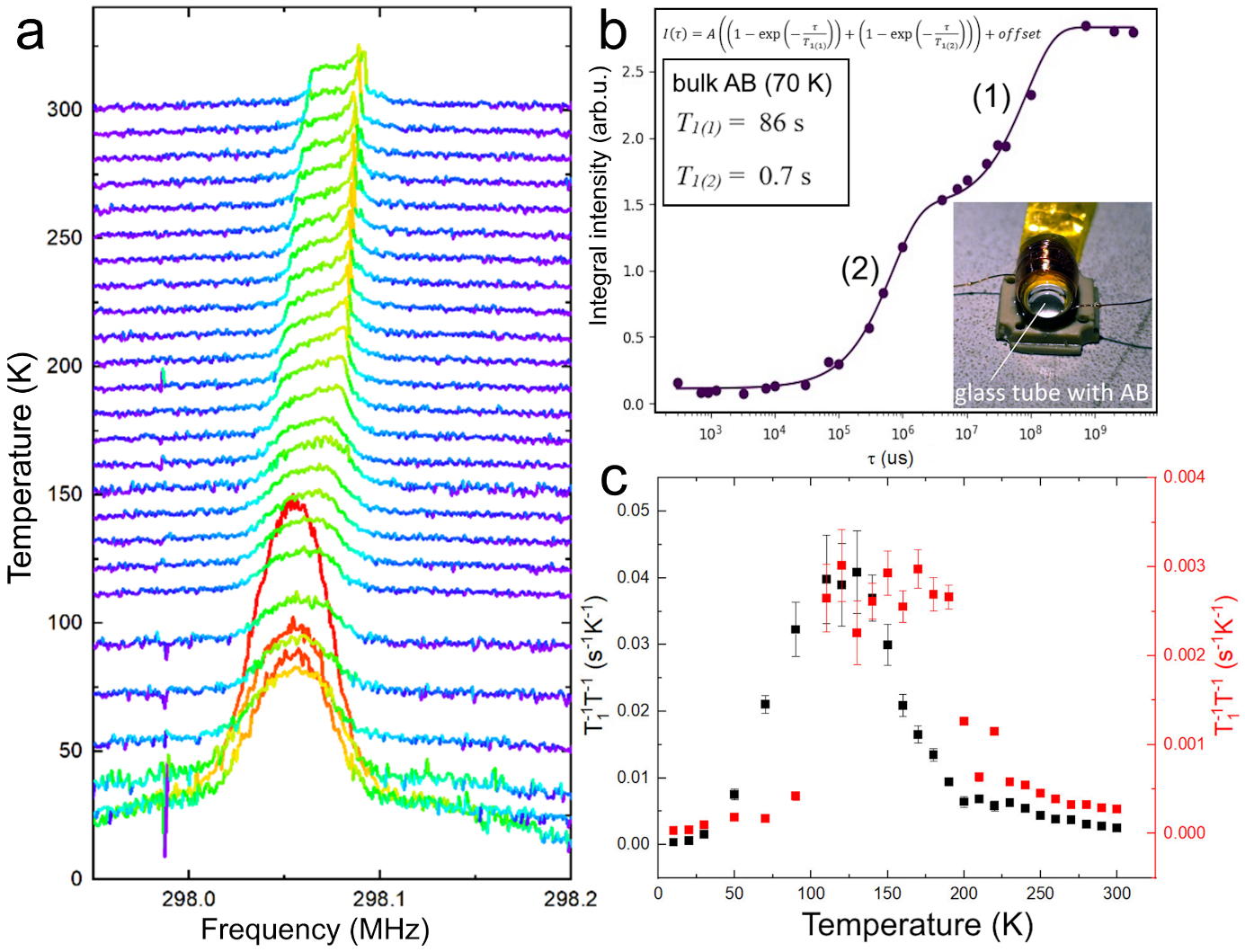


**Fig. S9.** ^1^H NMR data of a bulk NH_3_BH_3_ sample placed in a solenoid. (a) Stack of ^1^H NMR spectra at different temperatures during cooling from 300 to 10 K. (c) Spin-lattice relaxation rate 1/*TT*_1_ vs *T*, obtained by the two-component fit. There is a pronounced jump in relaxation processes below 210 K in both components. (d) Integrated intensity *I* at 70 K as a function of spin-echo delay time τ, compared to a fit describing a two-component relaxation. Inset: picture of the used solenoid with a glass ampoule (Wilmad-LabGlass, NMR sample tubes) containing AB (Sigma Aldrich).

In order to determine the temperature dependence of the ^1^H NMR *T*_1_ for NH_3_BH_3_ and contrast it to the behavior of the superhydrides, we measured a bulk sample of NH_3_BH_3_ in a conventional setup (i.e., without DAC and Lenz lenses). For this, we prepared AB powder in a glass tube and used a standard solenoid as excitation coil (inset of Fig. S9b). The resulting ^1^H NMR spectra of AB do not show a significant intensity loss at low temperatures, as is observed for the DACs. Instead, at low temperatures, the intensity increases, similarly to the behavior of the samples inside the DAC.

We measured *T*_1_ by using the same procedures as described above. We note that the relaxation curves show a clear step, which indicates that the relaxation consists of two *T*_1_ components. We can describe this by the following equation

$I\left( \tau\right)=M_{z0}\left( \left( 1-\exp\left( -\frac{\tau}{T_{1(1)}} \right) \right)+\left( 1-\exp\left( -\frac{\tau}{T_{1(2)}} \right) \right) \right)+offset$*,* (S2)

where *T*_1(1)_ = 86 s, and *T*_1(2)_ = 0.7 s at 70 K, $M_{z0}$ and $offset$ are defined as in (S1). We show the temperature dependence of both components in Fig. S10c. At around 210 K, both components yield the onset of a distinct peak. Gunaydin-Sen *et al.* have observed a similar behavior in a ^15^N NMR study of AB at 225 K and attribute it to a phase transition, accompanied by a change of molecular motions^[78,96]^. Importantly, the high-temperature regime exhibits a very different behavior from what we observe in our superhydride samples in the DACs.

~~
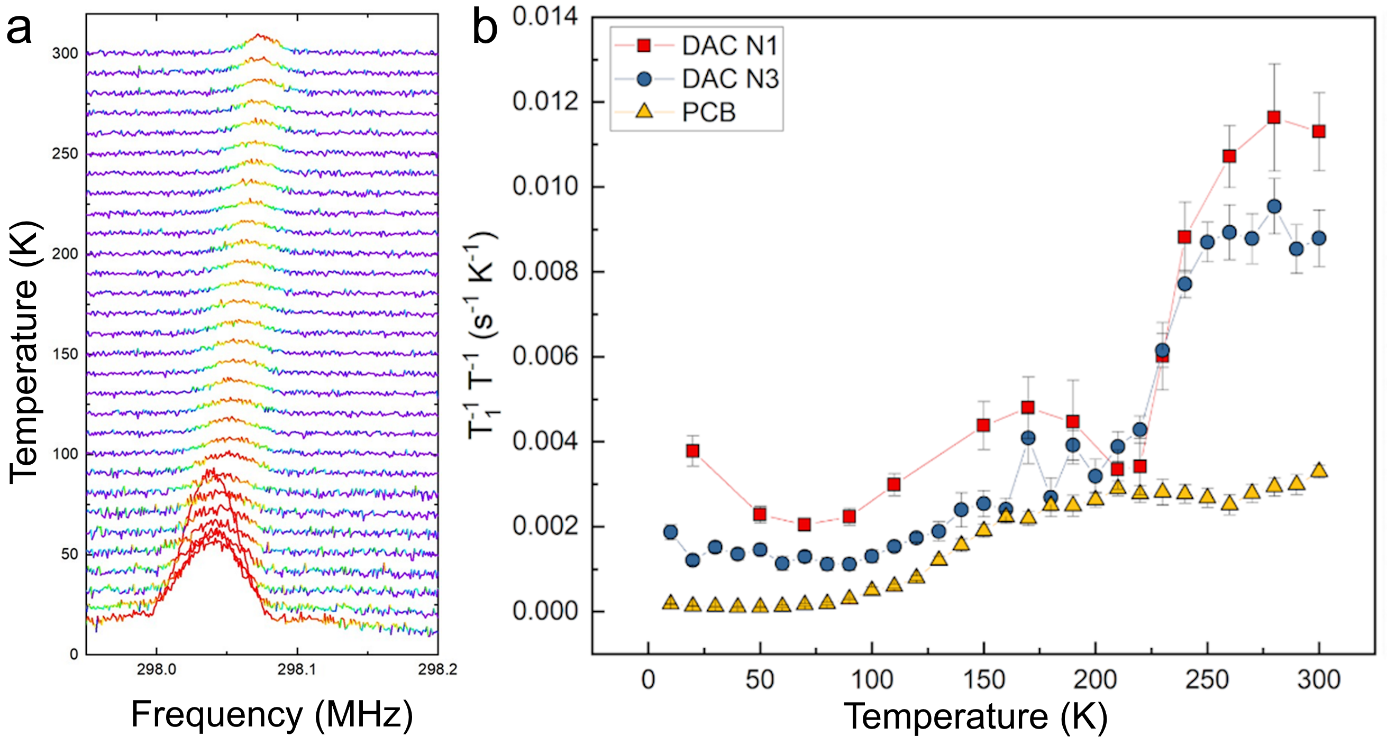
~~

**Fig. S10.** ^1^H NMR data for a piece of ~150 µm thick Teflon printed circuit board (PCB), used as a base for the single-turn excitation coil in the DAC experiments. (a) Stack of ^1^H NMR spectra at different temperatures during cooling from 300 to 10 K. (b) Dependence of the spin-lattice relaxation rate 1/*TT_1_* vs *T* of the PCB in comparison with the behavior of superhydrides in DACs N1 and N3.

To determine another contribution to the background signal, we measured the temperature dependence of the ^1^H NMR spectra of the PCB, used in the DAC preparation. We observe a shift of the spectrum towards low temperatures, which might yield a minor contribution to the overall spectral shift observed in our other measurements (Fig. S10). The intensity increases strongly at low temperatures, which is a common feature in all our ^1^H NMR measurements, even in the measurements of AB in a conventional setup. Thus, the origin of this behavior may relate to residual Hydrogen in the setup. In any case, this behavior is not related to our observations for the superhydride samples.

**5. COMSOL modeling**

The dissipated power density *P_d_* or, in other words, the surface loss density (SLD) in W/m^2^ can be calculated in COMSOL Multiphysics 6.0^[79]^ using

$P_{d}=\frac{1}{2}\left( \bar{J_{s}}\cdot\bar{E^{*}} \right)=\frac{1}{2}t\sigma\left( \bar{E}\cdot\bar{E^{*}} \right)= \frac{1}{2}t\sigma{\bar{|E}|}^{2}= \frac{1}{2\sigma}t{\bar{|J}|}^{2}$, (S2)

where *J_s_* = *J*‧*t* is the induced surface current density, *t* the thickness of the metal layer, * denotes the complex conjugate, σ is the conductivity, and $\bar{E}$ is the electric-field vector. In the COMSOL simulations, we studied how the SLD and with it the electric and magnetic fields of a radio-frequency current are distributed in a thin metal layer Lenz lens deposited on a diamond anvil. In our qualitative numerical experiment, we set the thin copper layer to a thickness of 5 or 10 μm.


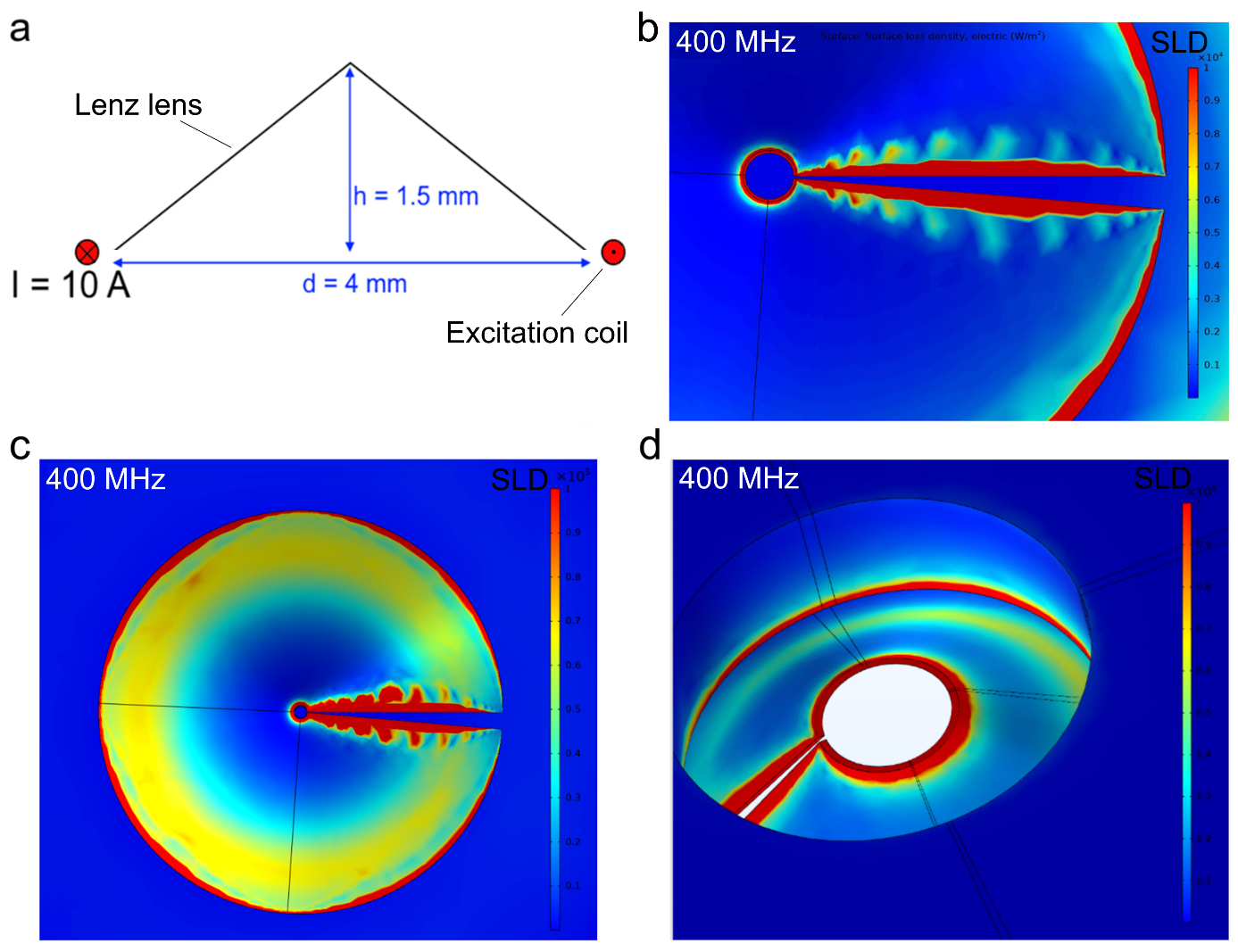


**Fig. S11.** Numerical modeling of the surface-loss density distribution in a conical Lenz lens. (a) Geometric model of the conical lens used for the numerical simulations. We used a point ring current (radius is 2.5 mm) source with a current of 10 A and frequency of 400 MHz. The diameter of the hole at the top of the conical Lenz lens is 200 μm, and the thickness of the metal layer of the Lenz lens is 5 μm. (b) Current distribution (SLD) near the longitudinal slot. Current and SLD reach high values in the vicinity of the diamond anvil culet. (c) The case of a vertical shift of the ring-inductor by 0.7 mm upward. The yellow stripe corresponds to the induction on the side surface of the lens. (d) Induction in a metal gasket (300 μm thick) placed on top of a diamond anvil with a Lenz lens.

As a result of our numerical modeling, we found that a lens without a longitudinal section (slit) has a maximum SLD near the inductor and a minimum near the diamond-anvil culet. The induced current in the lens tends to suppress the electromagnetic field created by the inductor. SLD around the culet changes dramatically in the presence of a longitudinal slot (Fig. 11b). Near the diamond anvil culet, the electromagnetic field now reaches its maximum. Moreover, a decrease in the diameter of the culet (that is, the cut diameter of the tip of the lens cone) causes a strong increase in the SLD and lens efficiency. Reducing the diameter by two times leads to an increase in SLD also by about two times as well.

As the frequency decreases, the current distribution becomes less localized, but the SLD maximum is still near the diamond anvil culet. A study of the influence of the inductor position shows that increasing the diameter of the inductor greatly reduces the electromagnetic field in the culet area. Downward movement also has a negative effect, but upward shift along the lens is not as critical (Fig. S11c). When adding a metal seat, we observe the occurrence of electromagnetic induction in the seat material as well (Fig. S11d). This phenomenon has a negative impact on the amount of energy transferred from the inductor to the Lenz lens.

**6. X-ray diffraction data**

**Table S3.** Experimental unit-cell parameters of the lanthanum polyhydrides *hP*-LaH_12_ and *C*2/*m*-LaH_12+x_ used to interpret our X-ray diffraction data of the sample in DAC N1 at 165 GPa (VASP format ^[16]^).

| ***hP*-LaH_12_ (165 GPa)** | ***C*2/*m*-LaH_12±x_ (165 GPa, x ~ 1)** |
| --- | --- |
| 1.0  4.80499 0.0 0.0  -2.40249 4.16125 0.0  0.0 0.0 3.77270  La  2  Direct  0.33333 0.66666 0.75  0.66666 0.33333 0.25 | 1.0  3.43899 0.0 0.00000  0.0 5.874 0.0  -1.55725 0.0 4.14299  La  2  Direct  0.0 0.5 0.5  0.5 0.0 0.5 |

**
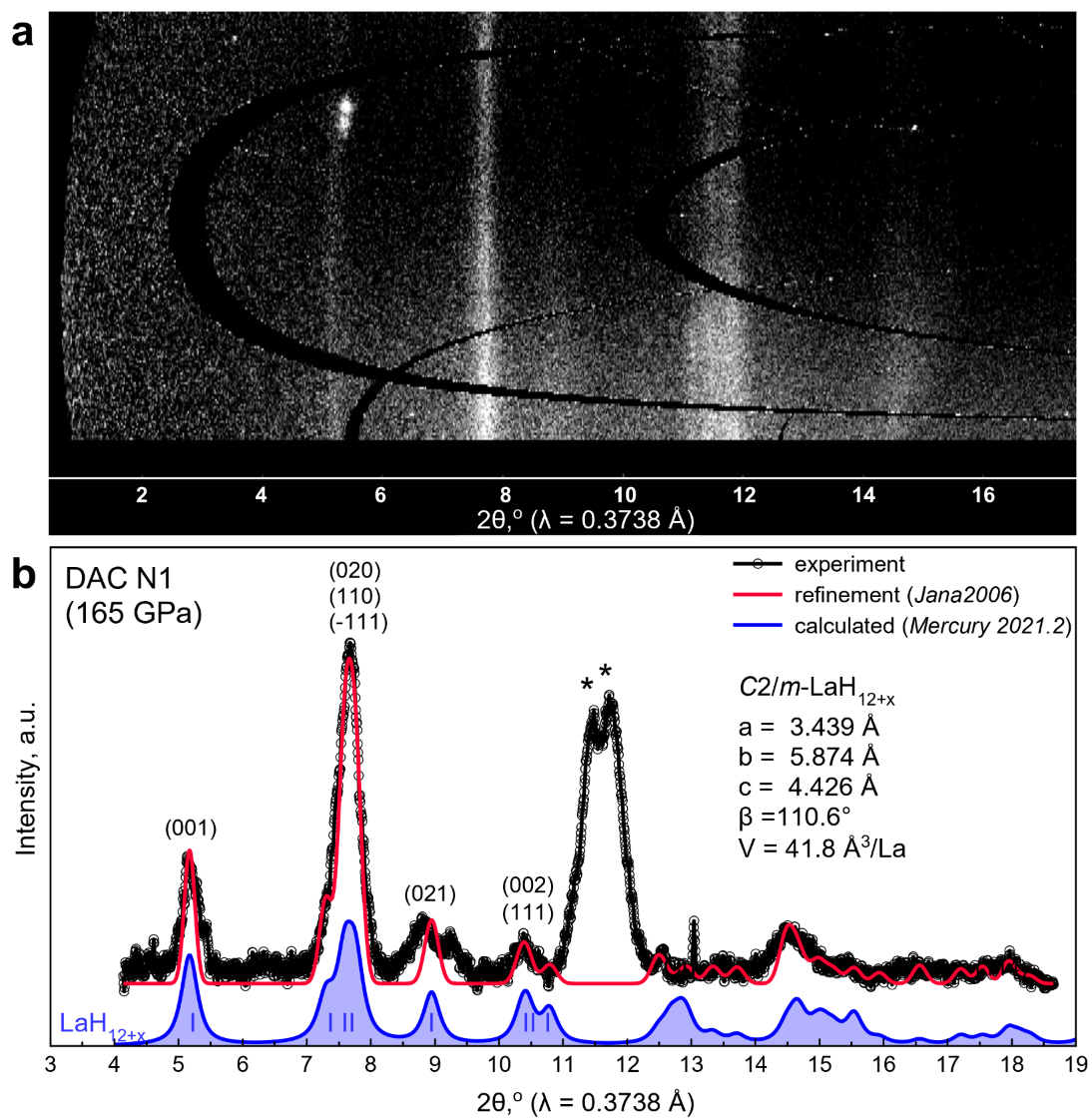
**

**Fig. S12.** X-ray-diffraction data of the sample in DAC N1 at 165 GPa. (a) X-ray diffraction image (so called “cake”) of the sample in DAC N1. Plot (b) represents the integrated intensity of the plot (a) at the corresponding diffraction angle. (b) X-ray diffraction pattern of the sample in DAC N1 and Le Bail refinement of *C*2*/m*-LaH_12±x_ (x ~ 1) unit-cell parameters. Black circles are the experimental data, the red line is the Le Bail refinement, and the blue line is the XRD pattern calculated using the Mercury 2021.2 software^[67]^. Asterisks indicate unidentified peaks. For the refinement, we used well-known prototype structure, *C*2/*m*-LaH_10_ ^[54]^, found by evolutionary structure search utilizing the USPEX code^[17,18,97,98]^. This structure does not explain the strong and broad reflections at 11-12 deg. Its refined unit-cell volume corresponds to a higher hydrogen content of about 12±1 H atoms for each La atom. Given the presence of the not interpreted reflections, we may speculate on a hexagonal structure of *hP*-LaH_12_ (Fig. 2a of the main text).

If we assume that the diffraction peak at 5.2° (Fig. S12) is associated with an impurity, a description via *Fm*$\bar{3}$*m*-LaH_10_ will also fail due to the low volume of such a cubic structure, V ≈ 28.5 Å^3^/La. Instead, structures such as *C*2/*m*-LaH_6_, *Cmme*-LaH_6_, and *R*$\bar{3}$*m*-LaH_6_ can, in general, explain the observed XRD pattern. They are expected from a theoretical point of view^[59]^ and were probably obtained experimentally^[28]^. However, all these structures lead to a unit cell volume that is too small or too large for the amount of hydrogen given in chemical formulas. Therefore, at the moment, the best description of the X-ray data is obtained using the hexagonal *hP*-LaH_12_ structure.


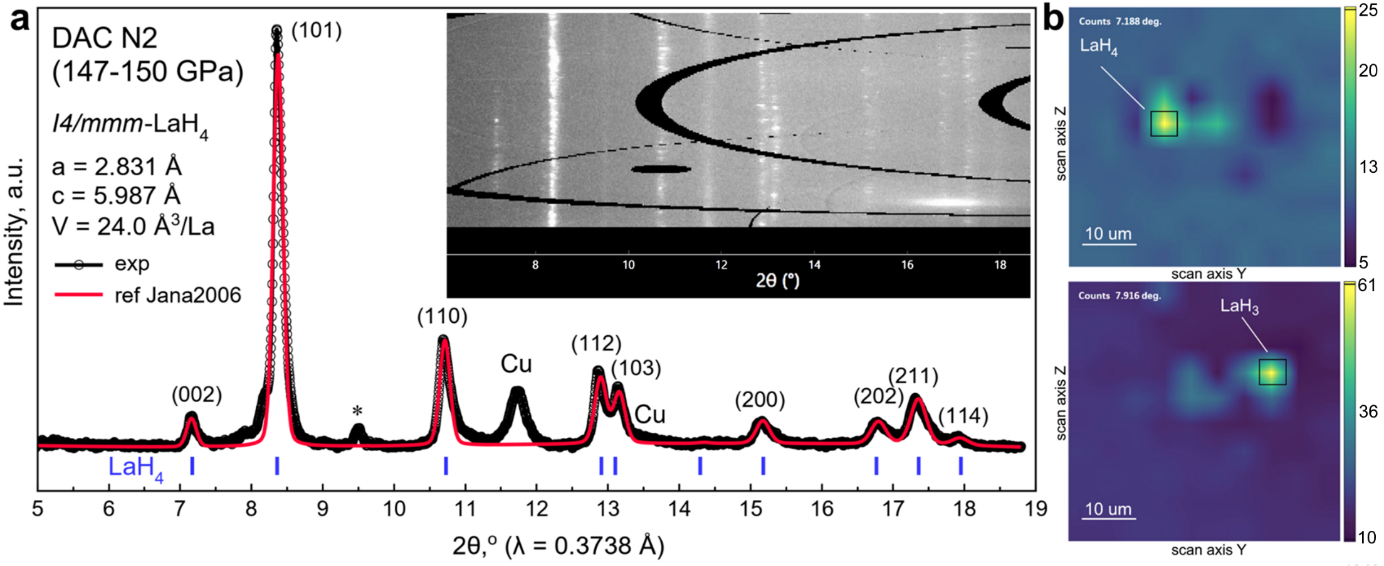


**Fig. S13.** X-ray diffraction in DAC N2. (a) XRD pattern of DAC’s N2 sample and Le Bail refinement of the tetragonal LaH_4_ unit cell parameters at 147-150 GPa. Black circles are the experimental data, red line is the refinement (Jana2006 ^[94]^), “Cu” corresponds to peaks from a copper Lenz lens. Inset: corresponding XRD image (“cake”). An asterisk (*) indicates an uninterpreted impurity signal. (b) X-ray diffraction mapping of the sample area using XDI software ^[68]^. One can see the spatial distribution of the two main hydride phases in the sample: *I*4/*mmm*-LaH_4_ and *Cmcm*-LaH_3_.

**
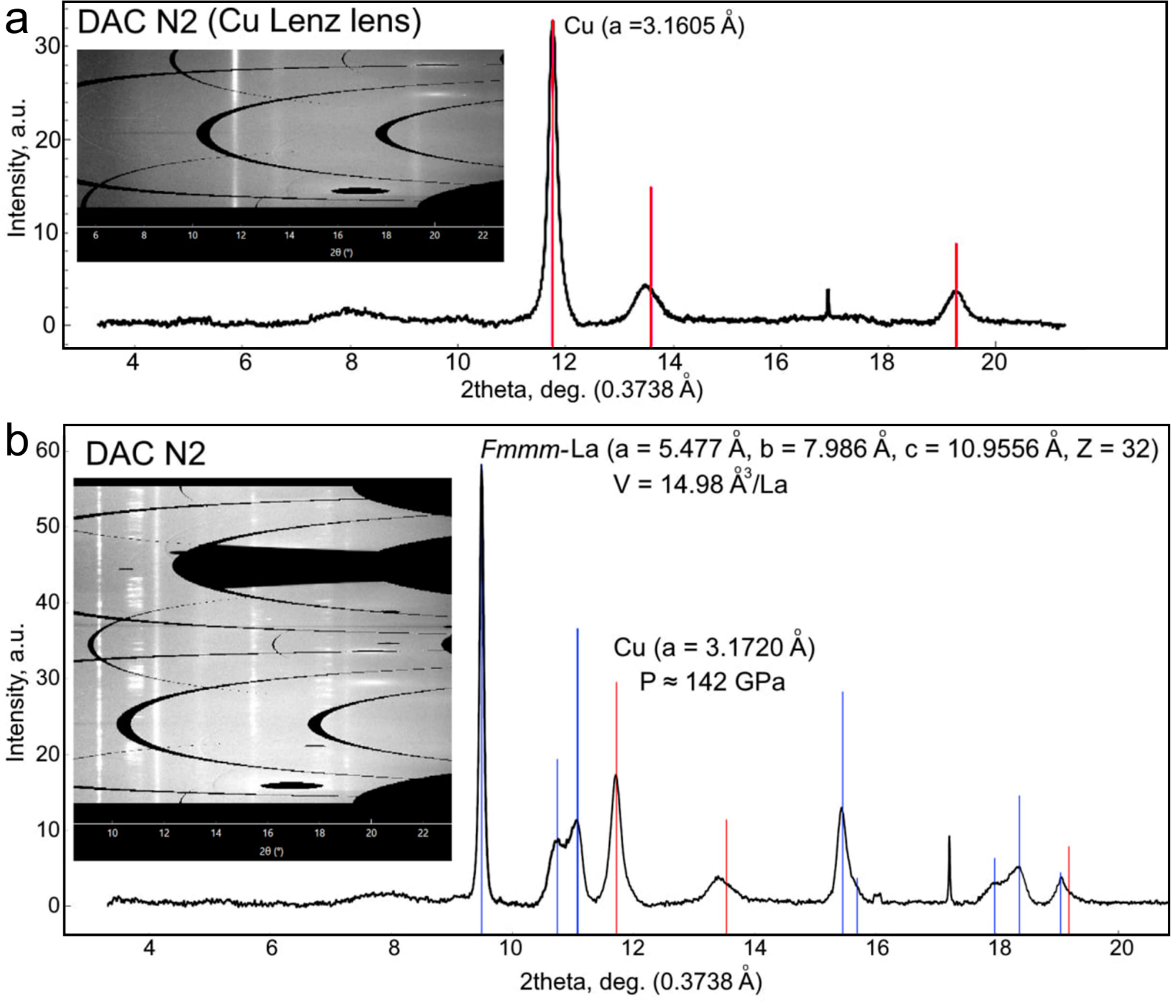
**

**Fig. S14.** X-ray diffraction studies of DAC N2 at 147-150 GPa. This study, performed before the laser heating of the sample, shows the presence of only (a) Lenz lenses material (copper) and (b) lanthanum metal (*Fmmm*-La), the unit cell volume of which is in good agreement with earlier experiments ^[99]^.


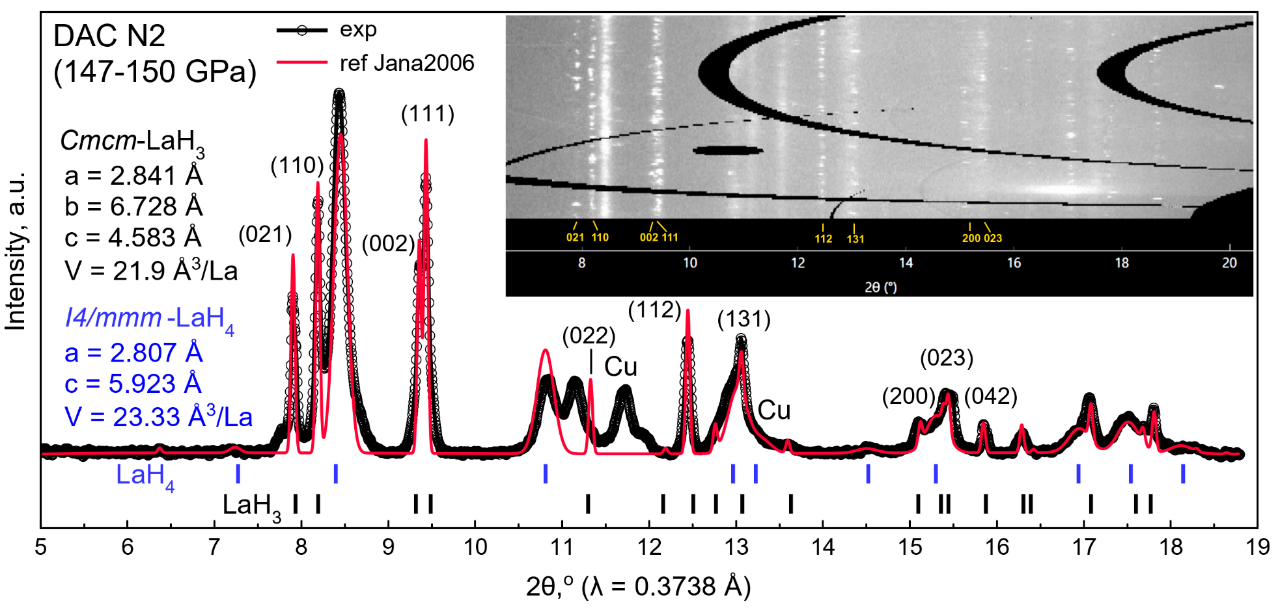


**Fig. S15.** X-ray diffraction pattern of DAC N2 and Le Bail refinement of the *Cmcm*-LaH_3_ and *I*4/*mmm*-LaH_4_ unit cell parameters. The compounds are clearly distinguishable since the X-ray diffraction pattern of LaH_3_ has a “spot-like” coarse-crystalline character. *Cmcm*-LaH_3_ can be predicted theoretically using an evolutionary search for stable crystal structures. At 100 GPa the LaH_3_ is 0.47 meV/atom above the convex hull of the La-H system at 100 GPa which is not so far ^[59]^.


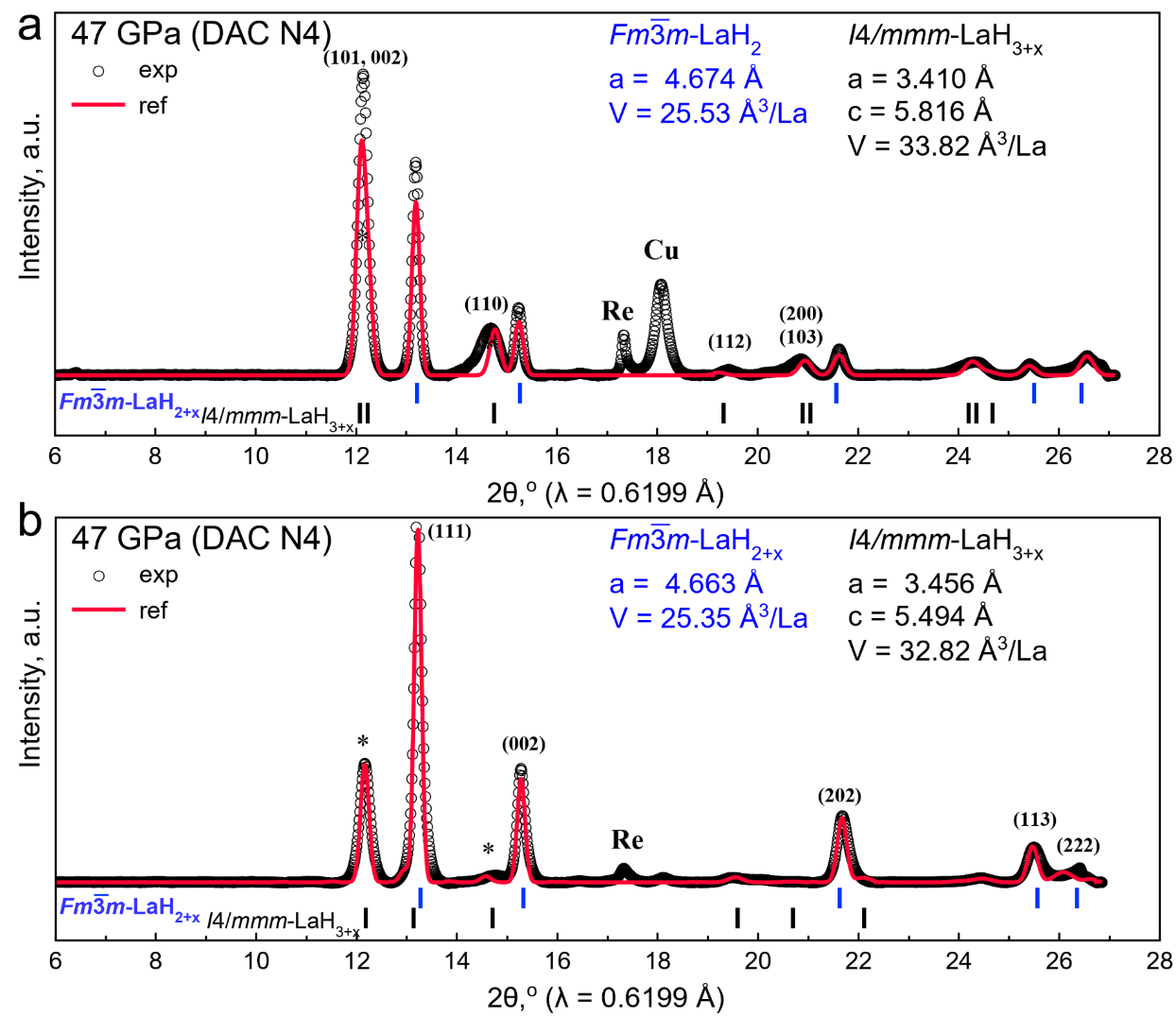


**Fig. S16.** X-ray diffraction pattern of DAC N4 and Le Bail refinement of the *Fm*$\bar{3}$*m*-LaH_2+x_ and *I*4/*mmm*-LaH_3+x_ unit cell parameters before the ^1^H NMR experiment. Black circles are the experimental data, red line is the refinement (Jana2006 ^[94]^), “Re” corresponds to peaks from a rhenium gasket. After low-temperature NMR measurements pressure in the DAC N4 dropped to 19 GPa. Panels (a) and (b) correspond to different regions in the sample.


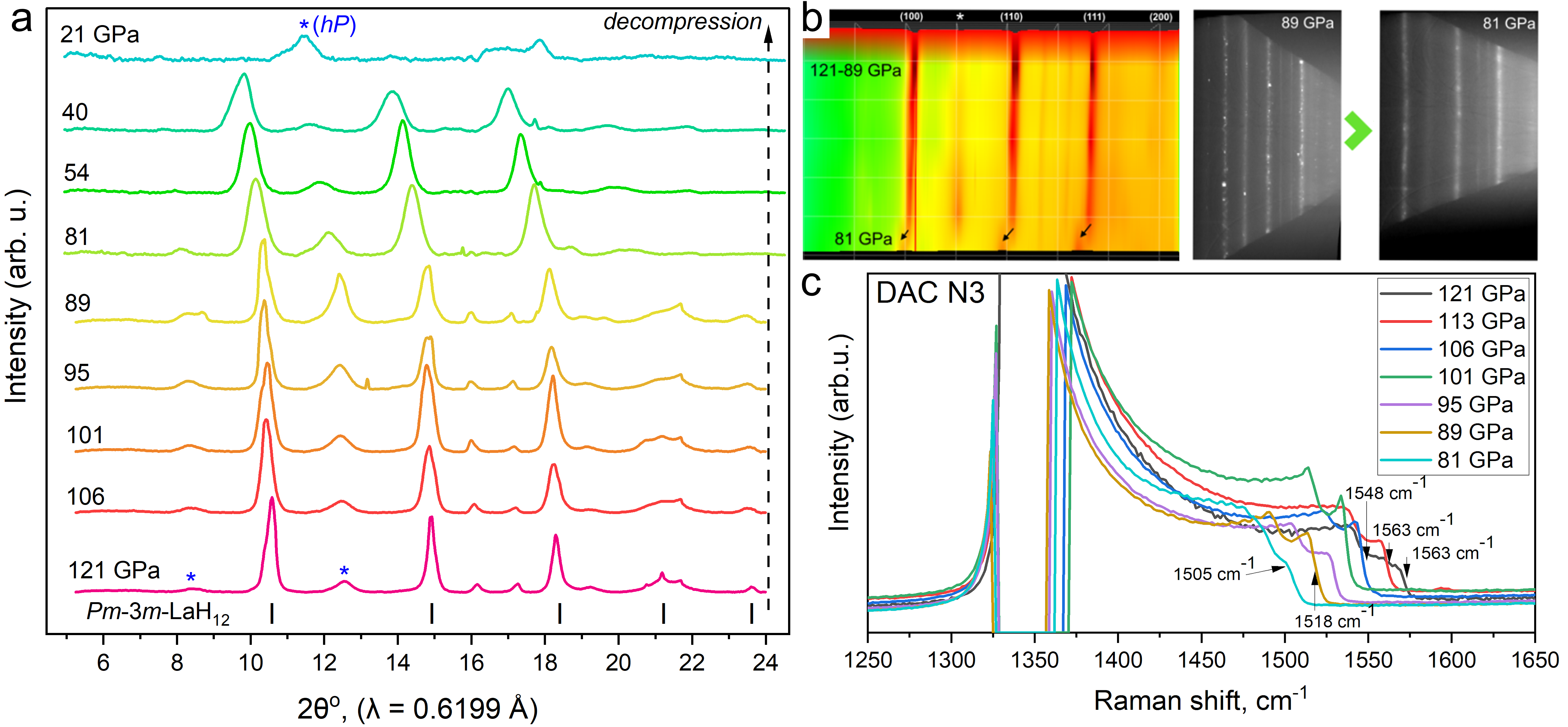


**Fig. S17.** Decompression of DAC N3. (a) Stack of integrated XRD patterns obtained during decompression of the DAC N3 with simple cubic *Pm*$\bar{3}$*m*-LaH_12_ from 121 GPa to 21 GPa at room temperature. Asterisks indicate reflections from the *hP*-LaH_12_. Decomposition point of sc-LaH12 is between 40 and 21 GPa. *hP* modification is more stable and still can be seen at 21 GPa. (b) Colormap of the XRD peaks in the diffraction pattern of sc-LaH­_12_ during decompression. A sharp change in the morphology of microcrystals can be seen between 89 and 81 GPa. This is likely due to a change in the crystal structure of the hydrogen sublattice. (c) Raman spectra for selected pressure points in the region of the diamond Raman signal measured in the center of the DAC N3 during decompression.

**7. Raman spectroscopy**

The purpose of the Raman spectroscopy was to establish the pressure before and after NMR experiments in DACs N0-N4. We found that during sample cooling to 10 K with subsequent warming up back to the room temperature, the pressure change in the DACs is within 5-10 GPa. Examples of pressure measurements before the experiment (the Akahama scale ^[100]^) are shown in Fig. S18.


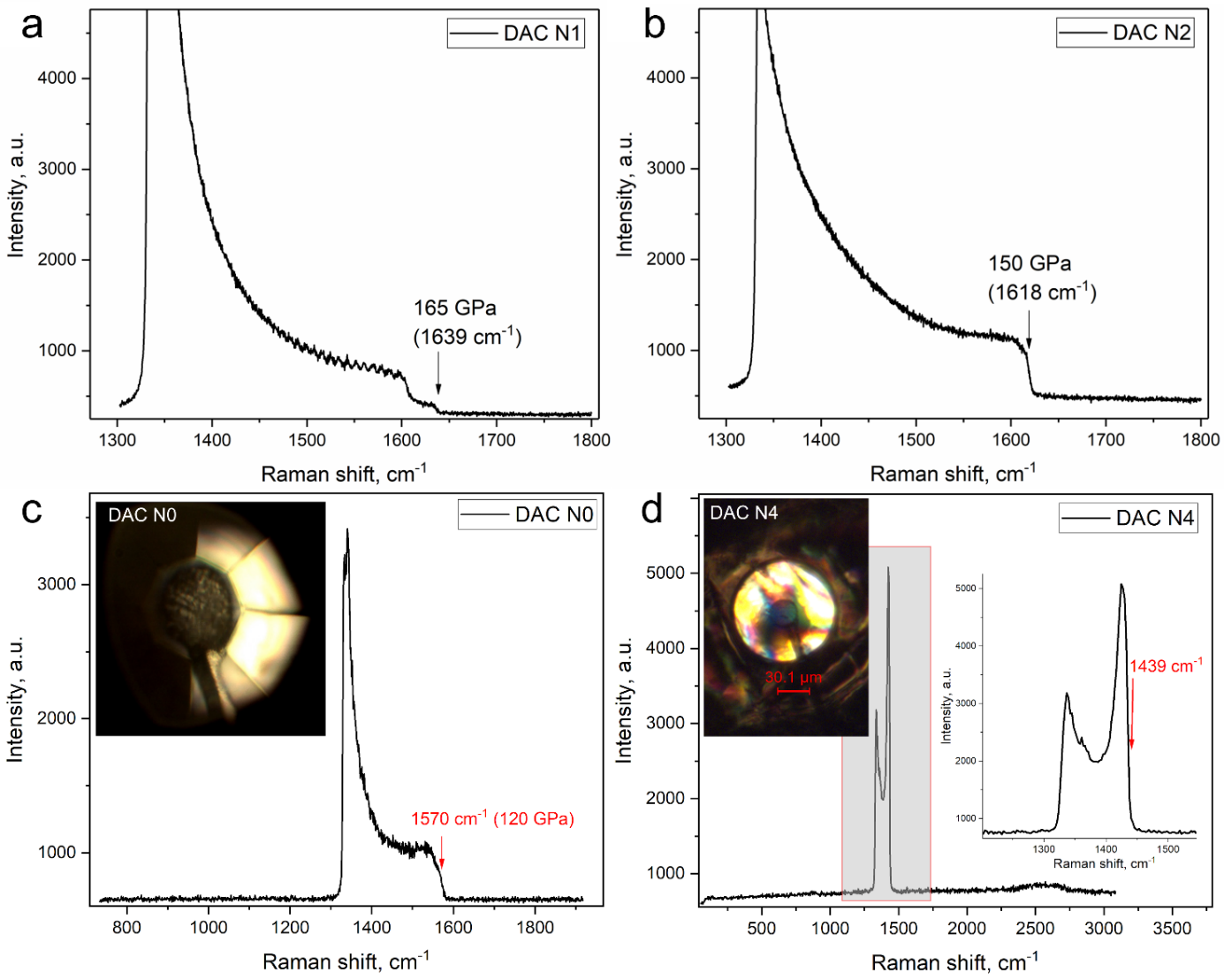


**Fig. S18.** Raman spectra of the samples in (a) DAC N1 (b), DAC N2 (c), DAC N0, and (d) DAC N4 before the NMR experiment. We used lasers with wavelengths of 633 nm and 532 nm for excitation. The Akahama scale^[100]^ was applied to determine the pressure. After the experiment at 5 K, we found that the pressure in DAC N4 dropped to 19 GPa. Insets: Photographs of the loaded high-pressure chambers of (c) DACs N0 and (d) DAC N4.

**8. Additional NMR data**

*^1^H NMR of LaH_12_ (DAC N1)*

**
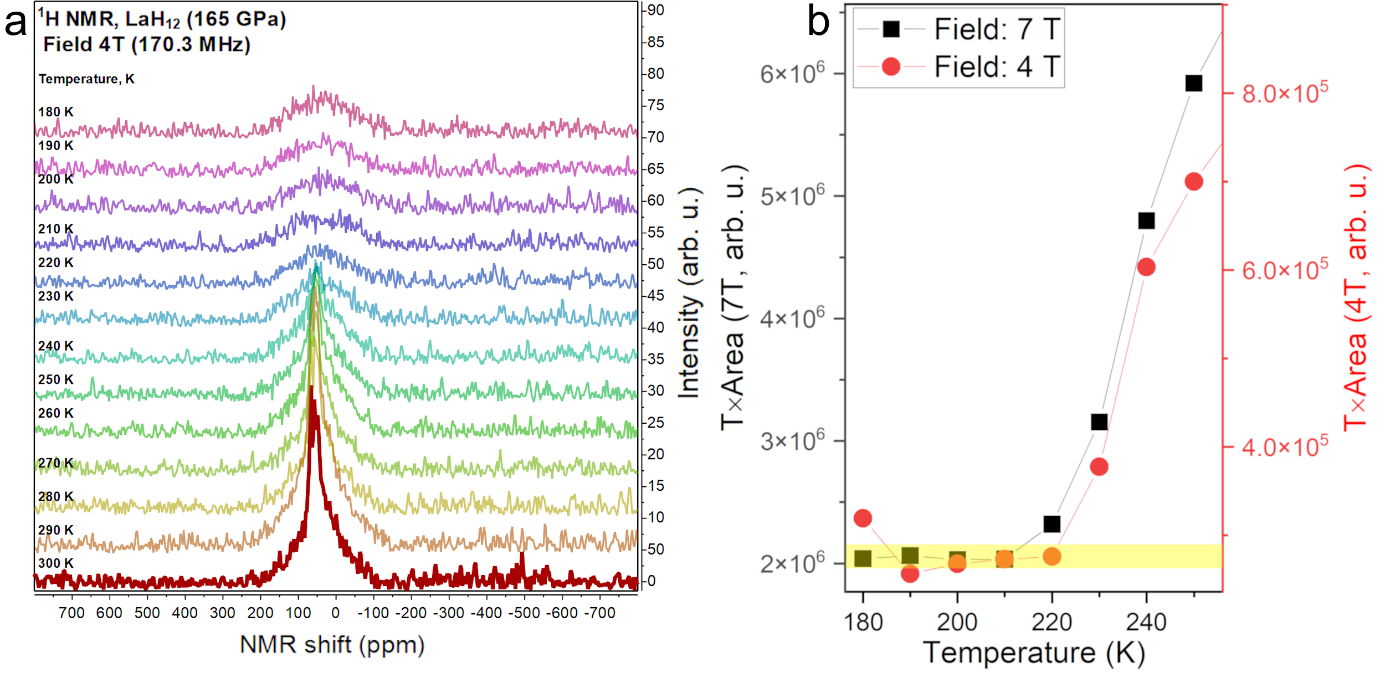
**

**Fig. S19.** NMR characteristics of LaH_12_ in DAC N1 sample in magnetic fields of 7 T and 4T at different temperatures. (a) Stack of ^1^H NMR spectra at various temperatures from 300 K to 180 K. (b) Superconducting transition in LaH_12_ at 165 GPa in 4 T and 7 T determined by the drop in the ^1^H NMR peak area times temperature. The offset transition temperature of 220 K at 4 T is noticeably higher than at 7 T (211 K).

Fig. S19 shows a comparison of the ^1^H NMR data of the sample in DAC N1 between 4 and 7 T. The temperature dependence of the ^1^H NMR spectra at 4 T (Fig. S19a) is qualitatively similar to the temperature dependence at 7 T (compare Fig. 3 in the main text). A comparison of the spectral properties at 4 and 7 T reveals a slightly higher *T*_c_ at the lower field of 4 T, as expected.

*Spin-lattice relaxation time (T*_1_*) of LaH_12_ (DAC N1)*

NMR is an effective method for determining the superconducting gap *Δ(T)*/*k*_B_, since in fully gapped superconductors at temperatures below *T*_C_, the spin-lattice relaxation rate is described by the exponential law $\frac{1}{T_{1}}\propto e^{-\frac{\Delta(0)}{k_{B}T}}$ ^[101]^. A fit or our 1/*T*_1_ data of LaH_12_ with this simple exponential function gives *Δ(0)*/*k_B_* = (307 ± 41) K, which equates to (26.5 ± 3.5) meV (Fig. S20a). This value of the superconducting gap is several times less than theoretical estimates of 60-80 meV ^[58,59]^. The probable cause of this discrepancy is the multiphase contribution to the ^1^H NMR signal of DAC N1:

$\frac{1}{T_{1}}=\frac{1}{T_{1}^{SC}}+\frac{1}{T_{1}^{N}}={Ae}^{-\frac{\Delta\left( 0 \right)}{k_{B}T}}+CT,$ (S3)

where we assumed the validity of the Heitler-Teller equation for the non-superconducting H-containing part of the system, providing the $T_{1}^{N}$ term. In this case, the fit gives *Δ(0)*/*k*_B_ = (427 ± 49) K, which equates to (37 ± 4) meV, and *R_Δ_* = 2*Δ(0)*/*k*_B_*T*_c_ = (3.76 ± 0.42) (Fig. S20c), in line with the weak-coupling BCS limit (3.54), but less than what the Migdal-Eliashberg theory predicts for the LaH_10_ (*R_Δ_* ~ 5.5^[59]^). A further improvement of the accuracy of the SC gap estimate requires hydride samples of higher quality and mass. We achieve a better agreement with the prediction when neglecting the anomaly below 227 K. This anomaly may be a Hebel-Slichter peak (Fig. S21). Using Eq. (S3) gives *Δ(0)*/*k*_B_ = (672 ± 42) K, which equates to (57.9 ± 3.6) meV, and *R_Δ_* = 2*Δ(0)*/*k*_B_*T*_c_ = (5.16 ± 0.32), with *T*_c_ = 260 K at 7 T (Fig. S20d).


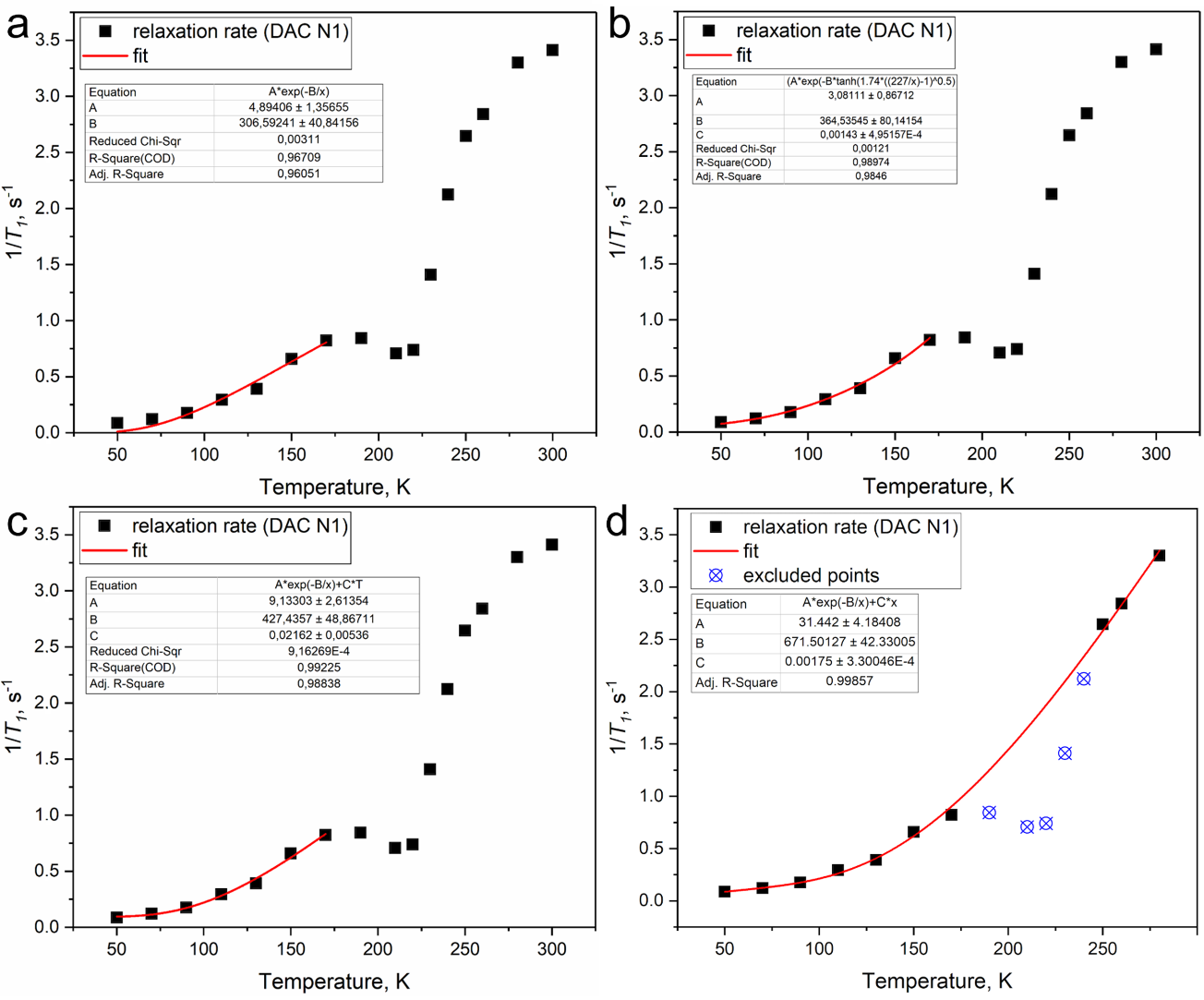


**Fig. S20.** Different fits to the temperature-dependent ^1^H NMR 1/*T*_1_ data at *T* < *T*_C_ for the sample in DAC N1 at 165 GPa and 7 T. (a) Using a simple exponential function for the data below 170 K gives *Δ(0)*/*k*_B_ = (307 ± 41) K. (b) Using an interpolation formula for *Δ(T)*/*k*_B_ proposed by Gross et al.^[102]^ for data below 170 K gives *Δ(0)*/*k*_B_ = (365 ± 80) K. (c) Considering the presence of a non-superconducting phase (1/*T_1_* $\propto CT$, Eq. S3) below 170 K results in *Δ(0)*/*k*_B_ = (427 ± 49) K. (d) Excluding data around the anomaly at 227 K and using Eq. S3 as well gives *Δ(0)*/*k*_B_ = (672 ± 42) K, which is the best agreement with the theoretical predictions.

**
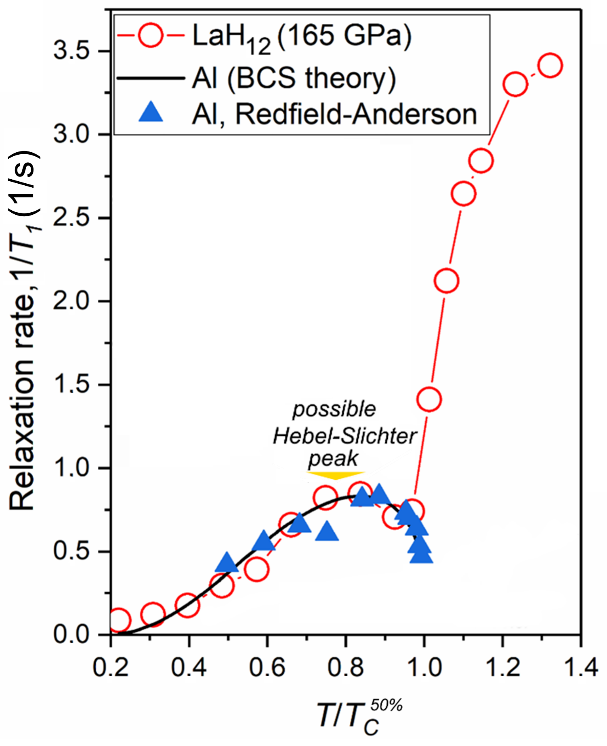
**

**Fig. S21.**  Temperature dependence of the ^1^H NMR spin-lattice relaxation rate of LaH_12_ (DAC N1) at 7 T (red circles) and aluminum (blue triangles, from Ref. ^21^) versus reduced temperature *T/T_c_^50%^*. The black line corresponds to the prediction of BCS (dirty limit)^[103]^.

Below 230 K, the temperature dependence of *1/T*_1_ of the sample in DAC N1 reveals a feature (Fig. S21), which compares well to the results measured by Anderson in aluminum^21^. Hebel and Slichter were the first to describe this behavior of *1/T*_1_ of aluminum in the superconducting state by applying the BCS theory^[38,39]^. If the feature observed in Fig. S21 is indeed a Hebel-Slichter peak, this would confirm the gapless (likely BCS-type) nature of the superconducting order parameter in LaH_12_. Although we note that, generally, the Hebel-Slichter peak is quite rarely observed experimentally and so far, this is the only sample, in which we observe this feature.

*^1^H NMR LaH_~3_ and LaH_4_ (DAC N2)*


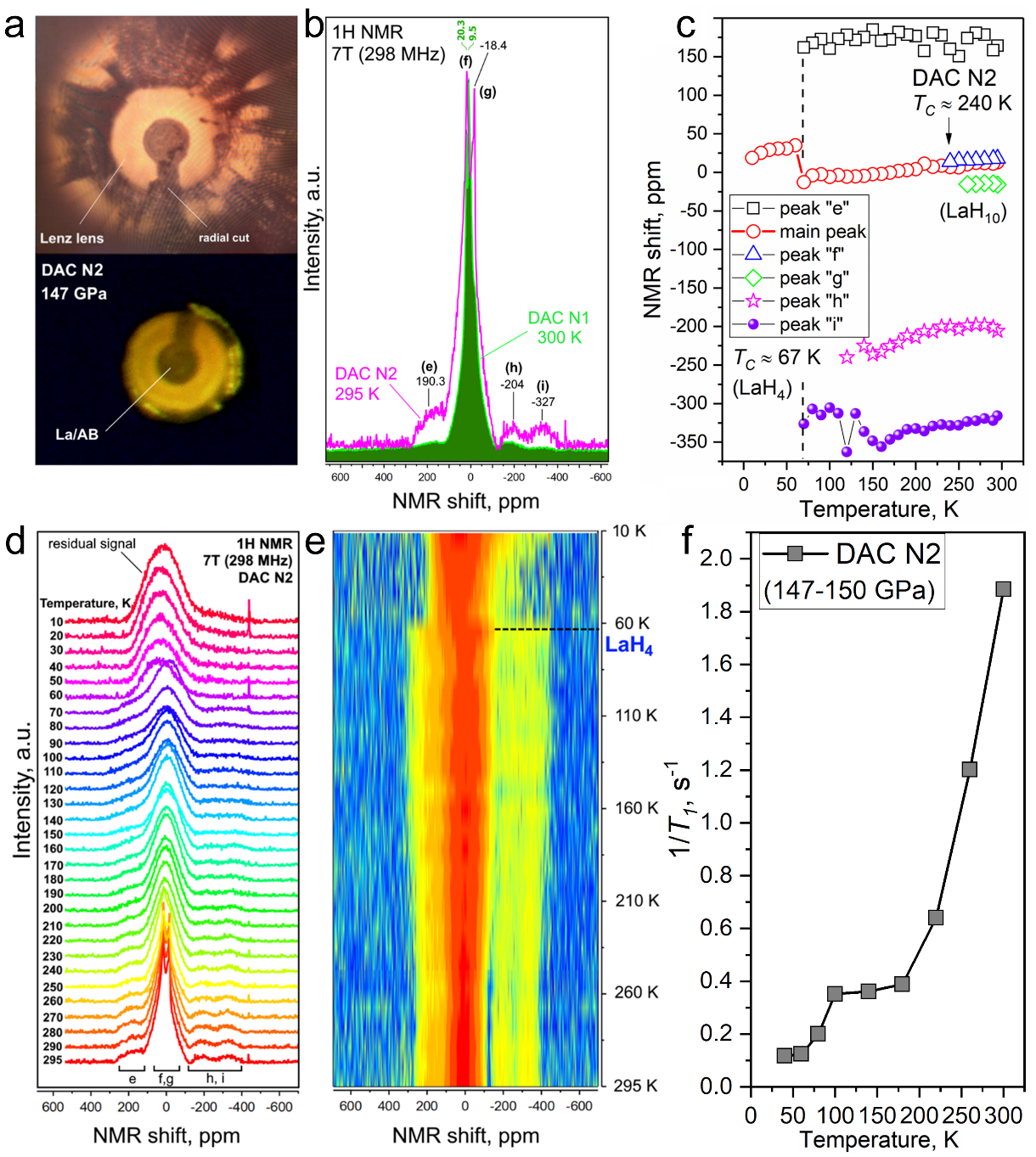


**Fig. S22.** NMR data of the sample in DAC N2. (a) Pictures of DAC N2 culet at 147 GPa. The geometry of the Lenz lens is clearly visible and remains unchanged under compression. (b) Comparison of ^1^H NMR spectra of the samples in DAC N1 (green) and DAC N2 (magenta) at a temperature of 300 and 295 K, respectively. In addition to a signal similar to that observed for the sample in DAC N1 (“f, g”), a series of broad signals “e, h, i” is observed, which may correspond to LaH_4_, since they disappear below 67 K. (c) Temperature dependence of the NMR shift of each component of the complex NMR signal. Two peaks, “f” and “g” disappear below 240 K, while the signals “e”, “h”, and “i” disappear below 60-70 K. (d) Stack of ^1^H NMR spectra of the sample in DAC N2 in 7 T at various temperatures from 295 to 10 K. (e) ^1^H NMR intensity colormap of the data presented in panel (d). Red regions have the highest intensity and blue ones the lowest intensity. The sharp change in the signal below 60-70 K may point to the SC transition in LaH_4_. (f) Temperature dependence of 1/*T*_1_ of the sample in DAC N2. Below about 100 K, 1/*T*_1_ drops. The midpoint of the transition is about 65-75 К.

Despite the small sample size, the NMR signal of the sample in DAC N2 has acceptable quality (Fig. S22). The disappearance of two narrow ^1^H NMR peaks “f” and “g” at temperatures below 250 K may be caused by the presence of small fractions of higher lanthanum hydrides (LaH_10_ or LaH_12_), missed due to the large scanning step (5 µm) used in the X-ray diffraction experiment. Around 60-70 K, all NMR signal characteristics (frequency shift, height, width, and area) undergo a pronounced change. This may be associated with the superconducting transition of LaH_4_ (Fig. S22 c,e,f).

The broad and weak NMR signals “e” and “i” (Fig. S22c) do not yield significant features in their temperature dependences, and disappear between 60 and 70 K. The same holds for the even weaker signal “h”, which disappears below 110-120 K (Fig. S22c). The relaxation time *T*_1_ of the sample in DAC N2 is about 530 ms, and *TT*_1_ ≈ 160 sK at 300 K. The relaxation rate yields a drop in the range of 60-70 K, which, however, starts much earlier, at around 100 K (Fig. S22f).

We studied the spin-spin relaxation time *T*_2_ of the sample in DAC N2 in a range from 5.5 K to 270 K. We found that *T*_2_ is 32 – 47 μs and almost independent of temperature (Supplementary Table S4). This situation of *T*_2_ << *T*_1_ is typical for solid-state NMR of metal hydrides (e.g., Ref. ^[104]^).

**Table S4.** Spin-spin relaxation time *T*_2_ of the sample in DAC N2, (147-150 GPa) in 7 T at different temperatures.

| **Temperature, K** | ***T_2_*, μs** | ***Error in T_2_, ε(%)*** |
| --- | --- | --- |
| 270 | 47.1 | 32.5 |
| 200 | 39.55 | 42 |
| 100 | 32.05 | 19 |
| 5.5 | 37.61 | 41.4 |

*^1^H NMR of LaH_~3_ (DAC N4)*

^1^H NMR studies of lower lanthanum hydrides were carried out using DAC N4 loaded with LaH_3-x_ and ammonium borane in 8 T magnetic field. The DAC was originally intended for research at high pressures, had a culet of 75 μm, and was heated using an IR laser at 150 GPa. However, a failure of one of the anvils led to a pressure drop to 47 GPa. After the low-temperature NMR experiment the pressure decreased further to 19 GPa. According to X-ray diffraction data, the sample consists of two phases: cubic (*fcc*) LaH_2+x_ and, probably, tetragonal (*I*4*/mmm*) LaH_3+x_ (Fig. S16). Both phases are not high-*T*_c_ superconductors. To simplify the further discussion, and considering the starting compound for the synthesis, we will henceforth refer to the sample in DAC N4 as LaH_3_.

The ^1^H NMR examination of this sample evidenced its non-superconducting nature (Fig. S23). During the cooling, the NMR signal broadens significantly and experiences a slight frequency shift of about 26 ppm. This emphasizes the absence of a pronounced Knight shift in many lanthanum hydrides. The width (FWHM) of the NMR signal below 100 K reaches 40-50 kHz, which corresponds to the width of the residual signal in the DACs N1-N3 at low temperatures. The spin-lattice relaxation time of LaH_3_ in DAC N4, measured at 5 K, is *T*_1_ ≈ 100 s, and *TT*_1_ ≈ 500 sK. Comparing with the low-temperature behavior of *T*_1_ in DACs N1 and N2, the differences in *TT_1_* are small: we found *TT*_1_ *=* 573 sK at 50 K in DAC N1, and 340 sK at 40 K in DAC N2. This is not surprising, given the fact that SC phases make virtually no contribution to the relaxation rate at much below *T_C_*. Therefore, the spin-lattice relaxation at low temperatures is mainly due to the residual non-SC LaH_3_ phase and other H-containing phases.

**
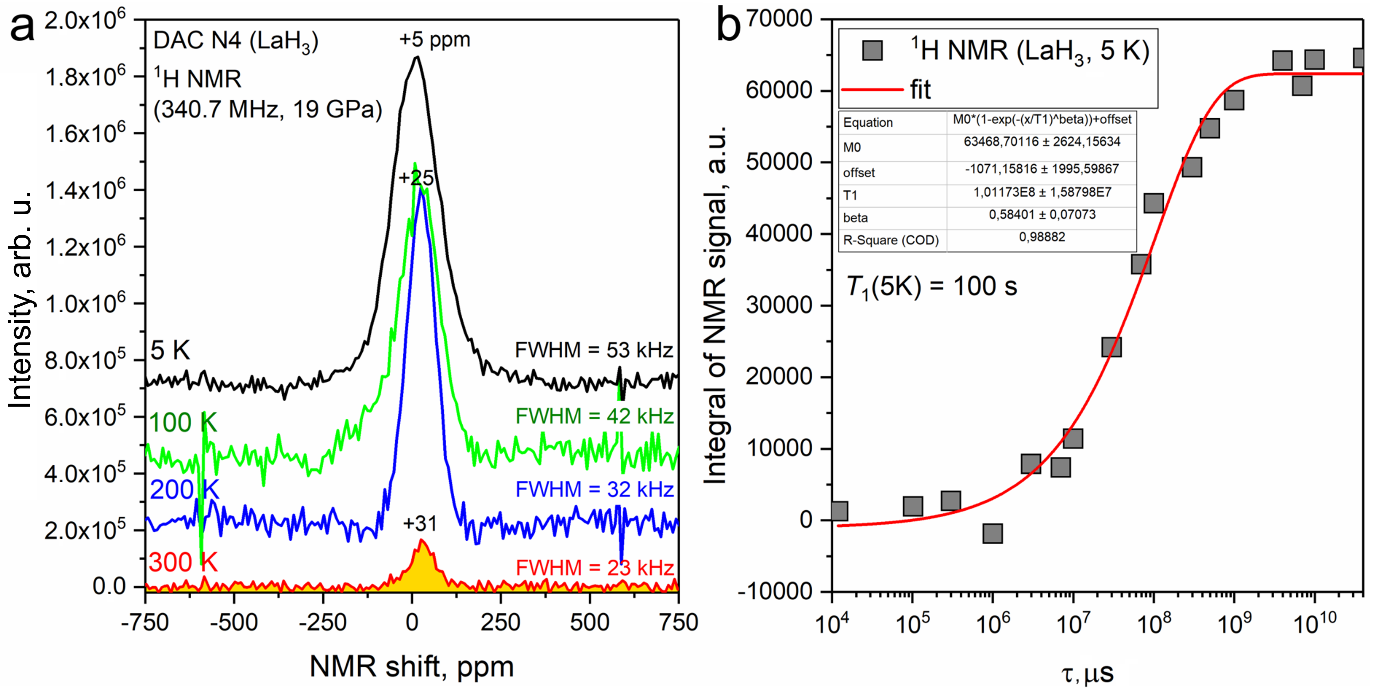
**

**Fig. S23.** ^1^H NMR of the sample in DAC N4. (a) Stack of ^1^H NMR spectra at 19 GPa in an 8 T field at 5, 100, 200, and 300 K. (b) Dependence of the integral intensity of the ^1^H NMR signal on the interpulse delay time τ within the spin-lattice relaxation experiment at 5 K. The found relaxation time *T*_1_ is about 100 s.

**References**

[92] M. R. Willcott, *J. Am. Chem. Soc.* **2009**, *131*, 13180-13180.

[93] C. Prescher, V. B. Prakapenka, *High Pressure Res.* **2015**, *35*, 223-230.

[94] V. Petříček, M. Dušek,L. Palatinus, *Z. Kristallogr.* **2014**, *229*, 345-352.

[95] A. Le Bail, *Powder Diffraction* **2005**, *20*, 316-326.

[96] N. Bloembergen, E. M. Purcell,R. V. Pound, *Physical Review* **1948**, *73*, 679-712.

[97] A. O. Lyakhov, A. R. Oganov, H. T. Stokes,Q. Zhu, *Comput. Phys. Commun.* **2013**, *184*, 1172-1182.

[98] G. M. Shutov, D. V. Semenok, I. A. Kruglov,A. R. Oganov, *Materials Today Physics* **2024**, *40*, 101300.

[99] W. Chen, D. V. Semenok, I. A. Troyan, A. G. Ivanova, X. Huang, A. R. Oganov,T. Cui, *Phys. Rev. B* **2020**, *102*, 134510.

[100] Y. Akahama, H. Kawamura, *J. Appl. Phys.* **2006**, *100*, 043516.

[101] M. Weger, *Pure and Applied Chemistry* **1972**, *32*, 325-338.

[102] F. Gross, B. S. Chandrasekhar, D. Einzel, K. Andres, P. J. Hirschfeld, H. R. Ott, J. Beuers, Z. Fisk,J. L. Smith, *Zeitschrift für Physik B Condensed Matter* **1986**, *64*, 175-188.

[103] R. Boyack, S. Mirabi,F. Marsiglio, *Commun. Phys.* **2023**, *6*, 54.

[104] C. D. Browning, T. M. Ivancic, R. C. Bowman,M. S. Conradi, *Physical Review B* **2006**, *73*, 134113.

[1] A. P. Drozdov, M. I. Eremets, I. A. Troyan, V. Ksenofontov,S. I. Shylin, *Nature* **2015**, *525*, 73-76.

[2] A. P. Drozdov, P. P. Kong, V. S. Minkov, S. P. Besedin, M. A. Kuzovnikov, S. Mozaffari, L. Balicas, F. F. Balakirev, D. E. Graf, V. B. Prakapenka, E. Greenberg, D. A. Knyazev, M. Tkacz,M. I. Eremets, *Nature* **2019**, *569*, 528-531.

[3] M. Somayazulu, M. Ahart, A. K. Mishra, Z. M. Geballe, M. Baldini, Y. Meng, V. V. Struzhkin,R. J. Hemley, *Phys. Rev. Lett.* **2019**, *122*, 027001.

[4] D. V. Semenok, A. G. Kvashnin, A. G. Ivanova, V. Svitlyk, V. Y. Fominski, A. V. Sadakov, O. A. Sobolevskiy, V. M. Pudalov, I. A. Troyan,A. R. Oganov, *Mater. Today* **2020**, *33*, 36-44.

[5] P. Kong, V. S. Minkov, M. A. Kuzovnikov, A. P. Drozdov, S. P. Besedin, S. Mozaffari, L. Balicas, F. F. Balakirev, V. B. Prakapenka, S. Chariton, D. A. Knyazev, E. Greenberg,M. I. Eremets, *Nat. Commun.* **2021**, *12*, 5075.

[6] I. A. Troyan, D. V. Semenok, A. G. Kvashnin, A. V. Sadakov, O. A. Sobolevskiy, V. M. Pudalov, A. G. Ivanova, V. B. Prakapenka, E. Greenberg, A. G. Gavriliuk, I. S. Lyubutin, V. V. Struzhkin, A. Bergara, I. Errea, R. Bianco, M. Calandra, F. Mauri, L. Monacelli, R. Akashi,A. R. Oganov, *Adv. Mater.* **2021**, *33*, 2006832.

[7] W. Chen, D. V. Semenok, X. Huang, H. Shu, X. Li, D. Duan, T. Cui,A. R. Oganov, *Phys. Rev. Lett.* **2021**, *127*, 117001.

[8] I. A. Troyan, D. V. Semenok, A. G. Ivanova, A. G. Kvashnin, D. Zhou, A. V. Sadakov, O. A. Sobolevsky, V. M. Pudalov,A. R. Oganov, *Phys. Usp.* **2022**, *65*, 748-761.

[9] D. Semenok. *Computational design of new superconducting materials and their targeted experimental synthesis* Doctoral program in matraials science and engineering thesis thesis, Skoltech, (2022).

[10] N. W. Ashcroft, *Phys. Rev. Lett.* **2004**, *92*, 187002-187001-187002-187004.

[11] F. Peng, Y. Sun, C. J. Pickard, R. J. Needs, Q. Wu,Y. Ma, *Phys. Rev. Lett.* **2017**, *119*, 107001.

[12] H. Liu, I. I. Naumov, R. Hoffmann, N. W. Ashcroft,R. J. Hemley, *PNAS* **2017**, *114*, 5.

[13] D. Duan, Y. Liu, F. Tian, D. Li, X. Huang, Z. Zhao, H. Yu, B. Liu, W. Tian,T. Cui, *Sci. Rep.* **2014**, *4*, 6968.

[14] P. Giannozzi, S. Baroni, N. Bonini, M. Calandra, R. Car, C. Cavazzoni, D. Ceresoli, G. L. Chiarotti, M. Cococcioni, I. Dabo, A. D. Corso, S. d. Gironcoli, S. Fabris, G. Fratesi, R. Gebauer, U. Gerstmann, C. Gougoussis, A. Kokalj, M. Lazzeri, L. Martin-Samos, N. Marzari, F. Mauri, R. Mazzarello, S. Paolini, A. Pasquarello, L. Paulatto, C. Sbraccia, S. Scandolo, G. Sclauzero, A. P. Seitsonen, A. Smogunov, P. Umari,R. M. Wentzcovitch, *J. Phys.: Condens. Matter* **2009**, *21*, 395502.

[15] S. Baroni, S. d. Gironcoli, A. D. Corso,P. Giannozzi, *Rev. Mod. Phys.* **2001**, *73*, 515-562.

[16] G. Kresse, J. Furthmüller, *Phys. Rev. B* **1996**, *54*, 11169.

[17] C. W. Glass, A. R. Oganov,N. Hansen, *Comput. Phys. Commun.* **2006**, *175*, 713-720.

[18] A. R. Oganov, C. W. Glass, *J. Chem. Phys.* **2006**, *124*, 244704.

[19] Y. Wang, J. Lv, L. Zhu,Y. Ma, *Phys. Rev. B* **2010**, *82*, 094116.

[20] C. J. Pickard, R. J. Needs, *J. Phys.: Condens. Matter* **2011**, *23*, 053201.

[21] M. Lüders, M. A. L. Marques, N. N. Lathiotakis, A. Floris, G. Profeta, L. Fast, A. Continenza, S. Massidda,E. K. U. Gross, *Phys. Rev. B* **2005**, *72*, 024545.

[22] M. A. L. Marques, M. Lüders, N. N. Lathiotakis, G. Profeta, A. Floris, L. Fast, A. Continenza, E. K. U. Gross,S. Massidda, *Phys. Rev. B* **2005**, *72*, 024546.

[23] I. Errea, M. Calandra,F. Mauri, *Phys. Rev. B* **2014**, *89*, 064302.

[24] D. V. Semenok, I. A. Troyan, D. Zhou, W. Chen, H.-k. Mao,V. V. Struzhkin, *The Innovation Materials* **2025**, 100115.

[25] D. V. Semenok, A. V. Sadakov, D. Zhou, O. A. Sobolevskiy, S. Luther, T. Helm, V. M. Pudalov, I. A. Troyan,V. V. Struzhkin, *Materials Today Physics* **2024**, 101595.

[26] D. V. Semenok, I. A. Troyan, D. Zhou, A. V. Sadakov, K. S. Pervakov, O. A. Sobolevskiy, A. G. Ivanova, M. Galasso, F. G. Alabarse,W. Chen, *arXiv:2408.07477* **2024**.

[27] A. Aslandukova, A. Aslandukov, D. Laniel, Y. Yin, F. I. Akbar, M. Bykov, T. Fedotenko, K. Glazyrin, A. Pakhomova, G. Garbarino, E. L. Bright, J. Wright, M. Hanfland, S. Chariton, V. Prakapenka, N. Dubrovinskaia,L. Dubrovinsky, *Sci. Adv.* **2024**, *10*, eadl5416.

[28] D. Laniel, F. Trybel, B. Winkler, F. Knoop, T. Fedotenko, S. Khandarkhaeva, A. Aslandukova, T. Meier, S. Chariton, K. Glazyrin, V. Milman, V. Prakapenka, I. A. Abrikosov, L. Dubrovinsky,N. Dubrovinskaia, *Nat. Commun.* **2022**, *13*, 6987.

[29] D. V. Semenok, B. L. Altshuler,E. A. Yuzbashyan, *arXiv:2407.12922* **2024**.

[30] T. Sakakibara, T. Goto,N. Miura, *Review of Scientific Instruments* **1989**, *60*, 444-449.

[31] Y. A. Timofeev, V. V. Struzhkin, R. J. Hemley, H.-k. Mao,E. A. Gregoryanz, *Rev. Sci. Instrum.* **2002**, *73*, 371-377.

[32] T. Meier, A. Aslandukova, F. Trybel, D. Laniel, T. Ishii, S. Khandarkhaeva, N. Dubrovinskaia,L. Dubrovinsky, *Matter and Radiation at Extremes* **2021**, *6*.

[33] H. Günther. *NMR Spectroscopy: Basic Principles, Concepts and Applications in Chemistry 3rd Edition*, John Wiley & Sons, **2013**.

[34] I. I. Rabi, J. R. Zacharias, S. Millman,P. Kusch, *Phys. Rev.* **1938**, *53*, 318-318.

[35] K. Müller, M. Geppi. *Solid State NMR: Principles, Methods, and Applications*, John Wiley & Sons, **2021**.

[36] L. N. Cooper. Microscopic Quantum Interference Effects in the Theory of Superconductivity. (1972).

[37] A. G. Anderson, A. G. Redfield, *Phys. Rev.* **1959**, *116*, 583-591.

[38] L. C. Hebel, C. P. Slichter, *Phys. Rev.* **1957**, *107*, 901.

[39] L. C. Hebel, C. P. Slichter, *Phys. Rev.* **1959**, *113*, 1504.

[40] W. D. Knight, *Phys. Rev.* **1949**, *76*, 1259-1260.

[41] D. F Smith, C. P Slichter. *The study of mechanisms of superconductivity by NMR relaxation*, **2006**.

[42] J. Korringa, *Physica* **1950**, *16*, 601-610.

[43] T. Meier, N. Wang, D. Mager, J. G. Korvink, S. Petitgirard,L. Dubrovinsky, *Sci. Adv.* **2017**, *3*, eaao5242.

[44] Y. Fu, R. Tao, L. Zhang, S. Li, Y.-N. Yang, D. Shen, Z. Wang,T. Meier, *Nat. Commun.* **2024**, *15*, 7293.

[45] T. Meier, F. Trybel, S. Khandarkhaeva, G. Steinle-Neumann, S. Chariton, T. Fedotenko, S. Petitgirard, M. Hanfland, K. Glazyrin,N. Dubrovinskaia, *Phys. Rev. X* **2019**, *9*, 031008.

[46] T. Meier, D. Laniel,F. Trybel, *Matter and Radiation at Extremes* **2023**, *8*.

[47] T. Meier, F. Trybel, G. Criniti, D. Laniel, S. Khandarkhaeva, E. Koemets, T. Fedotenko, K. Glazyrin, M. Hanfland, M. Bykov, G. Steinle-Neumann, N. Dubrovinskaia,L. Dubrovinsky, *Phys. Rev. B* **2020**, *102*, 165109.

[48] T. Meier, D. Laniel, M. Pena-Alvarez, F. Trybel, S. Khandarkhaeva, A. Krupp, J. Jacobs, N. Dubrovinskaia,L. Dubrovinsky, *Nat. Commun.* **2020**, *11*, 6334.

[49] M. Yang, Y. Zhou, R. Jana, T. Nakagawa, Y. Fu,T. Meier, *arXiv:2407.19368* **2024**.

[50] Y. L. Wu, X. H. Yu, J. Z. L. Hasaien, F. Hong, P. F. Shan, Z. Y. Tian, Y. N. Zhai, J. P. Hu, J. G. Cheng,J. Zhao, *Nature communications* **2024**, *15*, 9683.

[51] V. Struzhkin, B. Li, C. Ji, X.-J. Chen, V. Prakapenka, E. Greenberg, I. Troyan, A. Gavriliuk,H.-k. Mao, *Matter Radiat. Extremes* **2020**, *5*, 028201.

[52] T. Shitaokoshi, S. Kawachi, T. Nomura, F. F. Balakirev,Y. Kohama, *Review of Scientific Instruments* **2023**, *94*.

[53] N. Spengler, P. T. While, M. V. Meissner, U. Wallrabe,J. G. Korvink, *PLOS ONE* **2017**, *12*, e0182779.

[54] D. Sun, V. S. Minkov, S. Mozaffari, Y. Sun, Y. Ma, S. Chariton, V. B. Prakapenka, M. I. Eremets, L. Balicas,F. F. Balakirev, *Nat. Commun.* **2021**, *12*, 6863.

[55] A. P. Drozdov, V. S. Minkov, S. P. Besedin, P. P. Kong, M. A. Kuzovnikov, D. A. Knyazev,M. I. Eremets, *arXiv:1808.07039* **2018**.

[56] Y. Chen, J. Wen, Z.-X. He, J.-W. Fan, X.-Y. Pan, C. Ji, H. Gou, X. Yu, L. Chen,G.-Q. Liu. (2025).

[57] P. Dalladay-Simpson, G. Marchese, Z.-Y. Cao, P. Barone, L. Benfatto, G. Garbarino, F. Mauri,F. A. Gorelli. (2025).

[58] I. Errea, F. Belli, L. Monacelli, A. Sanna, T. Koretsune, T. Tadano, R. Bianco, M. Calandra, R. Arita, F. Mauri,J. A. Flores-Livas, *Nature* **2020**, *578*, 66-69.

[59] I. A. Kruglov, D. V. Semenok, H. Song, R. Szczęśniak, I. A. Wrona, R. Akashi, M. M. Davari Esfahani, D. Duan, T. Cui, A. G. Kvashnin,A. R. Oganov, *Phys. Rev. B* **2020**, *101*, 024508.

[60] D. V. Semenok, D. Zhou, J. Zhang, T. Helm, Y. Ding, H.-k. Mao,V. V. Struzhkin. (2025).

[61] D. Semenok, Troyan, I., Zhou, D., Sadakov, A. V., Pervakov, K. S., Sobolevskiy, O. A., Ivanova, A. G., Galasso, M., Alabarse, F. G., Chen, W., Xi, C., Helm, T., Luther, S., Pudalov, V. M., Struzhkin, V. V., *Adv. Funct. Mater.* **2025**, 2504748.

[62] D. V. Semenok, D. Zhou, W. Chen, A. G. Kvashnin, A. V. Sadakov, T. Helm, P. N. Ferreira, C. Heil, V. M. Pudalov, I. A. Troyan,V. V. Struzhkin, *Annalen der Physik n/a*, e00467.

[63] Z. Qiu, J. Chen, D. V. Semenok, Q. Zhong, D. Zhou, J. Li, P. Ma, X. Huang, M. Huo, T. Xie, X. Chen, H.-k. Mao, V. Struzhkin, H. Sun,M. Wang. (2025).

[64] I. Kantor, V. Prakapenka, A. Kantor, P. Dera, A. Kurnosov, S. Sinogeikin, N. Dubrovinskaia,L. Dubrovinsky, *Review of Scientific Instruments* **2012**, *83*, 125102.

[65] A. D. Grockowiak, M. Ahart, T. Helm, W. A. Coniglio, R. Kumar, K. Glazyrin, G. Garbarino, Y. Meng, M. Oliff, V. Williams, N. W. Ashcroft, R. J. Hemley, M. Somayazulu,S. W. Tozer, *Frontiers in Electronic Materials* **2022**, *2*.

[66] Q. Jiang, D. Duan, H. Song, Z. Zhang, Z. Huo, S. Jiang, T. Cui,Y. Yao, *Adv. Sci.* **2024**, *11*, 2405561.

[67] C. F. Macrae, I. Sovago, S. J. Cottrell, P. T. A. Galek, P. McCabe, E. Pidcock, M. Platings, G. P. Shields, J. S. Stevens, M. Towler,P. A. Wood, *J. Appl. Crystallogr.* **2020**, *53*, 226-235.

[68] R. Hrubiak, J. S. Smith,G. Shen, *Review of Scientific Instruments* **2019**, *90*, 025109.

[69] M. A. Kuzovnikov, presented at The 27th AIRAPT International Conference on High Pressure and Technology, Composition estimation of novel lanthanum superhydrides, Rio de Janeiro, Brasil (**2019**).

[70] M. A. Kuzovnikov, A. P. Drozdov, P. Kong, V. S. Minkov, S. P. Besedin, V. B. Prakapenka, E. Greenberg, D. A. Knyazev,M. I. Eremets, presented at 57th EHPRG Meeting on High Pressure Science and Technology, Crystal structures of novel lanthanum superhydrides, Prague, Czech Republic (**2019**).

[71] M. A. Kuzovnikov, presented at XXXVI Fortov International Conference on Interaction of Intense Energy Fluxes with Matter (ELBRUS 2021), V(P) equations of state of novel lanthanum and yttrium superhydrides, ELBRUS (**2021**).

[72] D. V. Semenok, I. A. Troyan, А. G. Kvashnin, A. G. Ivanova, M. Hanfland, A. V. Sadakov, O. A. Sobolevskiy, K. S. Pervakov, A. G. Gavriliuk, I. S. Lyubutin, K. Glazyrin, N. Giordano, D. Karimov, A. Vasiliev, R. Akashi, V. M. Pudalov,A. R. Oganov, *Mater. Today* **2021**, *48*, 18-28.

[73] A. H. Manayil Marathamkottil, K. Wang, N. P. Salke, M. Ahart, A. C. Mark, R. Hrubiak, S. Chariton, D. Smith, V. B. Prakapenka, M. Somayazulu, N. Velisavljevic,R. J. Hemley, *Nature communications* **2025**, *16*, 11222.

[74] D. Zhou, D. V. Semenok, D. Duan, H. Xie, X. Huang, W. Chen, X. Li, B. Liu, A. R. Oganov,T. Cui, *Sci. Adv.* **2020**, *6* eaax6849.

[75] J. Du, W. Sun, X. Li,F. Peng, *Physical Chemistry Chemical Physics* **2023**, *25*, 13320-13324.

[76] J. Bi, Y. Nakamoto, P. Zhang, Y. Wang, L. Ma, Y. Wang, B. Zou, K. Shimizu, H. Liu, M. Zhou, H. Wang, G. Liu,Y. Ma, *Mater. Today Phys.* **2022**, 100840.

[77] W. Chen, X. Huang, D. V. Semenok, S. Chen, D. Zhou, K. Zhang, A. R. Oganov,T. Cui, *Nat. Commun.* **2023**, *14*, 2660.

[78] O. Gunaydin-Sen, R. Achey, N. S. Dalal, A. Stowe,T. Autrey, *The Journal of Physical Chemistry B* **2007**, *111*, 677-681.

[79] Author. *COMSOL Multiphysics v. 6.0*, <<www.comsol.com>>

[80] A. A. Abrikosov, *J. Phys. Chem. Solids* **1957**, *2*, 199-208.

[81] A. V. Sadakov, V. A. Vlasenko, I. A. Troyan, O. A. Sobolevskiy, D. V. Semenok, D. Zhou,V. M. Pudalov, *J. Phys. Chem. Lett.* **2023**, *14*, 6666-6671.

[82] A. V. Sadakov, V. A. Vlasenko, D. V. Semenok, D. Zhou, I. A. Troyan, A. S. Usoltsev,V. M. Pudalov, *Phys. Rev. B* **2024**, *109*, 224515.

[83] V. S. Minkov, V. Ksenofontov, S. L. Bud’ko, E. F. Talantsev,M. I. Eremets, *Nat. Phys.* **2023**, *19*, 1293–1300.

[84] M. I. Eremets, V. S. Minkov, A. P. Drozdov,P. P. Kong, *Nat. Mater.* **2024**, *23*, 26-27.

[85] D. C. Cavanagh, B. J. Powell, *Physical Review Research* **2021**, *3*, 013241.

[86] R. Akis, J. P. Carbotte, *Solid State Communications* **1991**, *78*, 393-396.

[87] A. B. Migdal, *Sov. Phys. JETP* **1958**, *7*, 996.

[88] G. M. Eliashberg, *Sov. Phys. JETP* **1960**, *11*, 696-709.

[89] D. Semenok, J. Guo, D. Zhou, W. Chen, T. Helm, A. Kvashnin, A. Sadakov, O. Sobolevsky, V. Pudalov, V. Struzhkin, C. Xi, X. Huang,I. Troyan, *arXiv:2307.11742v2* **2023**.

[90] D. V. Semenok, I. A. Troyan, A. V. Sadakov, D. Zhou, M. Galasso, A. G. Kvashnin, A. G. Ivanova, I. A. Kruglov, A. A. Bykov, K. Y. Terent'ev, A. V. Cherepakhin, O. A. Sobolevskiy, K. S. Pervakov, A. Y. Seregin, T. Helm, T. Förster, A. D. Grockowiak, S. W. Tozer, Y. Nakamoto, K. Shimizu, V. M. Pudalov, I. S. Lyubutin,A. R. Oganov, *Adv. Mater.* **2022**, *34*, 2204038.

[91] Y. Song, C. Ma, H. Wang, M. Zhou, Y. Qi, W. Cao, S. Li, H. Liu, G. Liu,Y. Ma. (2025).

[92] M. R. Willcott, *J. Am. Chem. Soc.* **2009**, *131*, 13180-13180.

[93] C. Prescher, V. B. Prakapenka, *High Pressure Res.* **2015**, *35*, 223-230.

[94] V. Petříček, M. Dušek,L. Palatinus, *Z. Kristallogr.* **2014**, *229*, 345-352.

[95] A. Le Bail, *Powder Diffraction* **2005**, *20*, 316-326.

[96] N. Bloembergen, E. M. Purcell,R. V. Pound, *Physical Review* **1948**, *73*, 679-712.

[97] A. O. Lyakhov, A. R. Oganov, H. T. Stokes,Q. Zhu, *Comput. Phys. Commun.* **2013**, *184*, 1172-1182.

[98] G. M. Shutov, D. V. Semenok, I. A. Kruglov,A. R. Oganov, *Materials Today Physics* **2024**, *40*, 101300.

[99] W. Chen, D. V. Semenok, I. A. Troyan, A. G. Ivanova, X. Huang, A. R. Oganov,T. Cui, *Phys. Rev. B* **2020**, *102*, 134510.

[100] Y. Akahama, H. Kawamura, *J. Appl. Phys.* **2006**, *100*, 043516.

[101] M. Weger, *Pure and Applied Chemistry* **1972**, *32*, 325-338.

[102] F. Gross, B. S. Chandrasekhar, D. Einzel, K. Andres, P. J. Hirschfeld, H. R. Ott, J. Beuers, Z. Fisk,J. L. Smith, *Zeitschrift für Physik B Condensed Matter* **1986**, *64*, 175-188.

[103] R. Boyack, S. Mirabi,F. Marsiglio, *Commun. Phys.* **2023**, *6*, 54.

[104] C. D. Browning, T. M. Ivancic, R. C. Bowman,M. S. Conradi, *Physical Review B* **2006**, *73*, 134113.
